# *Theobroma cacao L.* pathogenesis-related gene tandem array members show diverse expression dynamics in response to pathogen colonization

**DOI:** 10.1186/s12864-016-2693-3

**Published:** 2016-05-17

**Authors:** Andrew S. Fister, Luis C. Mejia, Yufan Zhang, Edward Allen Herre, Siela N. Maximova, Mark J. Guiltinan

**Affiliations:** The Huck Institutes of the Life Sciences, The Pennsylvania State University, 422 Life Sciences Building, University Park, 16802 PA USA; Institute for Scientific Research and High Technology Services (INDICASAT-AIP), Panama City, Panama; Smithsonian Tropical Research Institute (STRI), Unit 9100, Box 0948, Balboa, Ancon, DPO AA 34002-9998 Panama; Department of Electrical Engineering, Princeton University, Princeton, NJ 08544 USA; The Department of Plant Science, The Pennsylvania State University, 422 Life Sciences Building, University Park, 16802 PA USA

**Keywords:** Pathogenesis-related, PR genes, PR proteins, Gene duplication, Tandem arrays, Disease resistance, Pathogen, *Phytophthora*, *Colletotrichum*

## Abstract

**Background:**

The pathogenesis-related (PR) group of proteins are operationally defined as polypeptides that increase in concentration in plant tissues upon contact with a pathogen. To date, 17 classes of highly divergent proteins have been described that act through multiple mechanisms of pathogen resistance. Characterizing these families in cacao, an economically important tree crop, and comparing the families to those in other species, is an important step in understanding cacao’s immune response.

**Results:**

Using publically available resources, all members of the 17 recognized pathogenesis-related gene families in the genome of *Theobroma cacao* were identified and annotated resulting in a set of ~350 members in both published cacao genomes. Approximately 50 % of these genes are organized in tandem arrays scattered throughout the genome. This feature was observed in five additional plant taxa (three dicots and two monocots), suggesting that tandem duplication has played an important role in the evolution of the PR genes in higher plants. Expression profiling captured the dynamics and complexity of PR genes expression at basal levels and after induction by two cacao pathogens (the oomycete, *Phytophthora palmivora*, and the fungus, *Colletotrichum theobromicola*), identifying specific genes within families that are more responsive to pathogen challenge. Subsequent qRT-PCR validated the induction of several PR-1, PR-3, PR-4, and PR-10 family members, with greater than 1000 fold induction detected for specific genes.

**Conclusions:**

We describe candidate genes that are likely to be involved in cacao’s defense against *Phytophthora* and *Colletotrichum* infection and could be potentially useful for marker-assisted selection for breeding of disease resistant cacao varieties. The data presented here, along with existing cacao—omics resources, will enable targeted functional genetic screening of defense genes likely to play critical functions in cacao’s defense against its pathogens.

**Electronic supplementary material:**

The online version of this article (doi:10.1186/s12864-016-2693-3) contains supplementary material, which is available to authorized users.

## Background

Plant-microbe interactions leading to pathogenesis or immunity rely on a complex series of interactions between host and microbial molecules. The process begins when plant membrane-bound pattern recognition receptors (PRRs) detect microbial- or pathogen-associated molecular patterns (MAMPs or PAMPs) [1], or intracellular R genes bind secreted microbial effector proteins [2–4]. Recognition of pathogen presence activates multiple signal transduction cascades, including several interacting phytohormone signaling systems [5], which organize local and systemic responses to the infection including the activation of genes encoding antimicrobial proteins and enzymes involved in the synthesis of secondary metabolites with antimicrobial activities [3, 6–9]. Ultimately, the plant’s survival hinges on its ability to rapidly produce peptides and chemicals with antimicrobial properties. Understanding this process is integral to breeding for or engineering more resistant plant cultivars, a dire need for improved global food security and sustainable agriculture.

Pathogenesis-related (PR) proteins, or as they have more recently been called, inducible defense-related proteins, have long been studied with regard to their importance in plant immunity [10, 11]. The 17 families of genes that fall under the broad ‘PR’ classification encode a group of proteins with various antimicrobial properties and that were originally identified because certain family members show strong induction in response to biotic stress associated with activation of systemic acquired resistance signaling [10]. Table 1 summarizes the roles of the 17 most commonly acknowledged PR families based on extensive work in a variety of species. Overall, the PR families encode a diverse array of proteins involved in pathogen defense though multiple mechanisms.

**Table 1:** Summary of PR gene families and their functions

PR gene class	Common name	Function	References
PR-1	None (CAP/SCP superfamily)	Unknown.	[10, 11, 56]
PR-2	β-1,3-glucanase	Aid in cell wall degradation.	[10, 11, 79]
PR-3	Chitinase–type I, II, IV, V, VI, VII	Aid in cell wall degradation.	[10, 11, 80, 81]
PR-4	Chitinase-Hevein-like	Aid in cell wall degradation. May have RNase and DNase activity.	[10, 11, 19, 80–83]
PR-5	Thaumatin-like	Degrade pathogen membranes.	[10, 11, 42, 84, 85]
PR-6	Proteinase-inhibitor	Inhibit proteolysis by herbivorous insects.	[10, 11, 42, 86]
PR-7	Endoproteinase	Aid in cell wall degradation.	[10, 11, 87]
PR-8	Chitinase-type III	Aid in cell wall degradation. May have lysozymal activity.	[10, 11, 80, 81, 88]
PR-9	Peroxidase	Regulate reactive oxygen species concentration, contribute to cell wall lignification.	[10, 11, 89]
PR-10	Ribonuclease-like	Degrade RNA, may degrade viruses.	[10, 11, 90, 91]
PR-11	Chitinase-type I	Aid in cell wall degradation.	[10, 11, 80, 81]
PR-12	Defensin	Degrade fungal membranes.	[10, 11, 92]
PR-13	Thionin	Directly permeabilize lipid bilayers.	[10, 11, 61]
PR-14	Lipid-transfer Protein	Degrade pathogen membranes, mechanism unclear.	[10, 11, 93]
PR-15	Germin/Oxalate Oxidase	Regulate reactive oxygen species production.	[11, 62, 94]
PR-16	Germin-like/Oxalate Oxidase-like	Regulate reactive oxygen species production, catalyze monosaccharides.	[11, 62, 94]
PR-17	Putative Zinc-metalloproteinase	Proteinase function probable, mechanism unclear.	[11, 95]

A better understanding of the defense response in crop plants is integral to increasing the sustainability of food and feed production. Cacao production around the world is severely inhibited by cacao’s susceptibility to pathogens, with roughly 40 % of the crop lost annually, accounting for a multi-billion dollar loss of cocoa trade and chocolate industry annually [12]. Two high-quality cacao genome sequences have been acquired, that of the fine-flavor Belizean Criollo genotype [13] and the widely-cultivated Matina genotype [14]. These resources enable new genome-wide strategies for characterizing the cacao defense response. To date, a handful of cacao PR genes have been studied, providing strong evidence that they play important roles in the response of cacao plants to pathogen infection. Application of glycerol to cacao leaves was recently found to promote defense and induce PR genes, likely through a fatty-acid-related signaling pathway [15]. The PR-1 s of cacao were recently identified, with at least one showing induction by *Moniliopthora perniciosa*, the causal agent of cacao’s witches broom disease [16]. Specific members of the PR-3 [17, 18],PR-4 [19], and PR-10 [20, 21] families have also been the subject of functional characterization, focusing on enzymatic properties and roles in defense. The results of a recent RNA-seq study measuring induction of genes by witches’ broom revealed that PR gene expression was elevated in infected tissues, but their induction (and induction of other known defense-related genes) was not sufficient to halt disease progression [22]. A study by our group used a microarray to measure the effect of salicylic acid treatment on two cacao genotypes [23]. Notably we found that PR gene induction levels differed between two contrasting genotypes, and surprisingly that more PR family members were induced in the more susceptible variety, ICS1, indicating that PR induction is only one piece of a successful defense response. Previously generated EST libraries [24, 25] and focused gene expression measurements [19, 23] have begun to characterize genotype specificity of the defense response in cacao, but much more work is required to characterize defense mechanisms across the described cacao populations [26]. Much more work is required to characterize the tissue specificity, induction, and function of these genes in cacao to understand and harness their potential for combating the diversity of cacao pathogens.

With the goal of better understanding the evolution, structure, and expression dynamics of the cacao PR gene families, we carried out a comprehensive annotation and analysis of all PR gene families and characterized their genomic organization and expression in response to pathogens. Using a comparative genomics approach, we found that in cacao and in five other diverse plant species (*Arabidopsis thaliana, Brachypodium distachyon, Oryza sativa, Populus trichocarpa, and Vitis vinifera*)*,* PR gene family sizes are similar and members are often physically clustered in tandem arrays, with more than half of the family members existing in these arrays. Analyzing existing EST databases, we found support for expression of 62 % of the *T. cacao* PR genes and identified many with expression limited to a specific tissues. Using a whole-genome microarray, we also identified PR gene family members induced by two major cacao pathogens, *Phytophthora palmivora* [27, 28] and *Colletotrichum theobromicola* [29], the causal agents of black pod rot and anthracnose, respectively*.* Comparing our new dataset to existing cacao transcriptomic analyses, we identified several PR genes strongly induced by multiple pathogens and treatments, suggesting potential roles as broad-spectrum defense response genes.

## Results

### Identification of cacao PR gene families

**Fig. 1:**
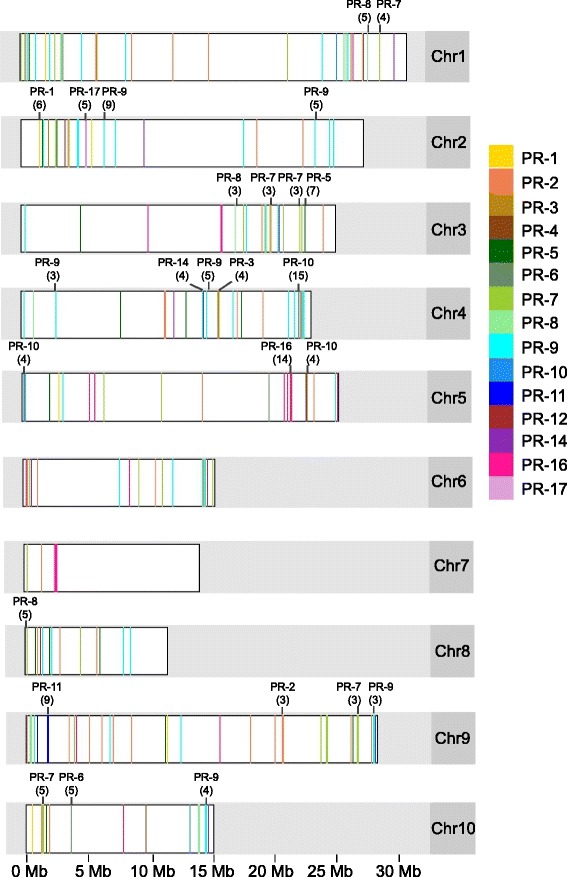
Karyogram depicting position of PR genes along the length of chromosomes based on the Criollo genome sequence. Tandem arrays are labelled above the chromosomes with gene family and number of genes in the array in parentheses. Length of chromosomes is shown in Mb. Due to resolution of the image lines representing nearby genes partially overlap

Using the Criollo cacao genome database (cocoagendb.cirad.fr/) [30], we developed a strategy for PR gene identification using the family type members described in van Loon et al. [11]*.* This bioinformatics approach resulted in a total of 359 PR genes identified in the Criollo genome (Table 2). Graphic representation of the genomic organization of these genes and the chromosomal positions of each of these loci is included in Fig. 1 and detailed information including gene IDs and chromosomal positions is provided in Additional file 1: Table S2. The process of gene identification was repeated for the Matina cacao genome [31].The Matina PR chromosomal distribution is plotted in Additional file 2: Figure S1 and Matina gene IDs and their positions are listed in Additional file 3: Table S3. Overall, the family sizes and genomic organization of the gene families in the two genomes was similar, however we observed some differences that could be the result of either chromosomal rearrangements or assembly errors. For the subsequent analysis, we focused on the genes identified in the Criollo genome assembly.

**Table 2:** Summary of PR gene families in the *Theobroma cacao* Criollo genome

Common name	Conserved domain	Number of loci in family	Best BLASTp hit (E-value)
PR-1 CAP domain protein	SCP (smart00198)	14	3.00E-53
PR-2 β-1,3-glucanase	glyco hydro 17 (pfam00332)	43	7.00E-102
PR-3 Chitinase Class I, II, IV, VII	chitinase glyco hydro 19 (cd00325)	11	3.00E-79
PR-4 Chitinase-Hevein-like	barwin (pfam00967)	8	3.00E-49
PR-5 Thaumatin-like	thaumatin (pfam00314)	30	5.00E-72
PR-6 Proteinase-inhibitor	potato inhibitor family (pfam00280)	8	5.00E-11
PR-7 Endoproteinase	PA subtilisin like (cd02120)	54	0
PR-8 Chitinase Class III	GH18 hevamine XipI class III (cd02877)	14	2.00E-91
PR-9 Peroxidase	secretory peroxidase (cd00693)	81	4.00E-113
PR-10 Ribonuclease-like	Bet v1 (pfam00407)	23	3.00E-48
PR-11 Chitinase class V	GH18 plant chitinase class v (cd02879)	11	3.00E-116
PR-12 Defensin	gamma-thionin (pfam00304)	3	7.00E-10
PR-13 Thionin	thionin (pfam00321)	0	NA
PR-14 Lipid-transfer Protein	nsLTP1 (cd01960)	16	6.00E-19
PR-15 Germin/Oxalate Oxidase	Two cupin 1 (pfam00190) domains	0	NA
PR-16 Germin-like/Oxalate Oxidase-like	Two cupin 1 (pfam00190) domains	38	2.00E-52
PR-17 Unknown	BSP (pfam04450)	5	7.00E-90
	Total	359 loci (38 unassembled)

**Fig. 2:**
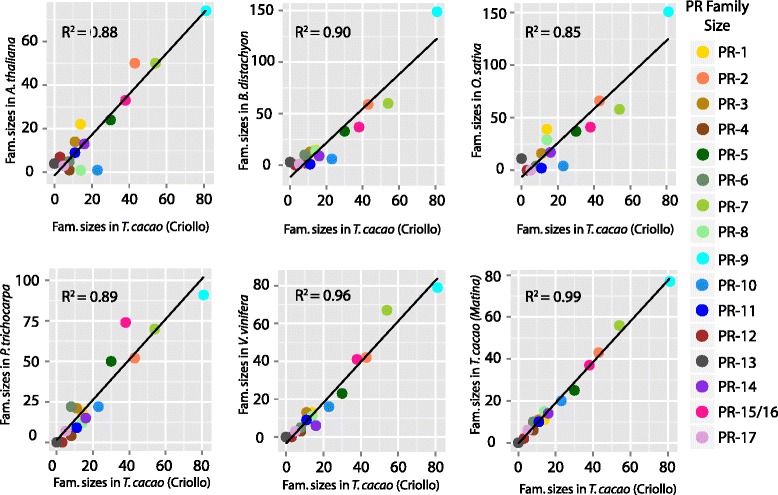
Scatterplots comparing PR gene family size in the in the Criollo *T. cacao* genome to five plant species and the Matina *T. cacao* genome

In order to determine whether PR family sizes in cacao were similar to those in other species, we next applied the PR gene identification pipeline to the *Arabidopsis thaliana* [32]*, Brachypodium distachyon* [33]*, Populus trichocarpa* [34], *Oryza sativa* [35]*,* and *Vitis vinifera* [36] genomes. PR genes identified in these species are listed in Additional file 4: Table S4, Additional file 5: Table S5, Additional file 6: Table S6, Additional file 7: Table S7, Additional file 8: Table S8. We found that in these species as in cacao, PR genes typically existed as families rather than as single genes, with a notable exception being that our strategy only identified one PR-4, PR-8, and PR-10 gene in the Arabidopsis genome. The size of gene families in cacao correlated well (R^2^ > .85, *p* < 0.001) with PR family sizes in the other species (Fig. 2). Family sizes in cacao were typical of those in the other dicots, with no major species-specific family expansions or reductions. We also noticed trends of family conservation across the plant genomes; PR-11 s were not found in the monocots (*Brachypodium distachyon* and *Oryza sativa*) surveyed, PR-12 s were only in Arabidopsis and cacao, and PR-13 s were found only in the monocots and Arabidopsis. The largest size disparity was in the PR-9 s, where the two monocots had ~150 members while the dicots had less than 100 members.

### Organization of PR gene families into tandem arrays

Criollo gene IDs indicate their order on chromosomes, where the first gene on chromosome 1 is Tc01_g000010, the second Tc02_g000020, etc. We noticed that many of the cacao PR genes were clustered with other members of the same family. To quantify this phenomenon, we defined a tandem array as any two or more genes of the same family that are located within 10 genes of one another [37, 38]. Using this parameter, we identified 46 PR tandem arrays containing a total 181 genes, distributed across all chromosomes (Fig. 1 and Additional file 1: Table S2). The number of genes within each tandem array ranged from2 to 16 across the families. The largest tandem arrays were a group of PR-10s on chromosome 4 (Chr4PR-10.6, 15 members), a group of PR-16 s on chromosome 5 (Chr5PR-16.3, 14 members), a group of PR-11 s on chromosome 9 (Chr9PR-11.1, 9 members), and a group of PR-9 s on chromosome 2 (Chr2PR-9.5, 9 members). Next, using JBrowse [39] we manually identified tandem arrays for each of the additional five species surveyed. We found that tandem arrays were very common across PR gene families in the diverse plant taxa surveyed (Additional file 9: Table S9), with more than half of the genes for most classes existing in tandem arrays. Proportions of PR family members found in tandem arrays, particularly among dicots, were also similar.

**Fig. 3:**
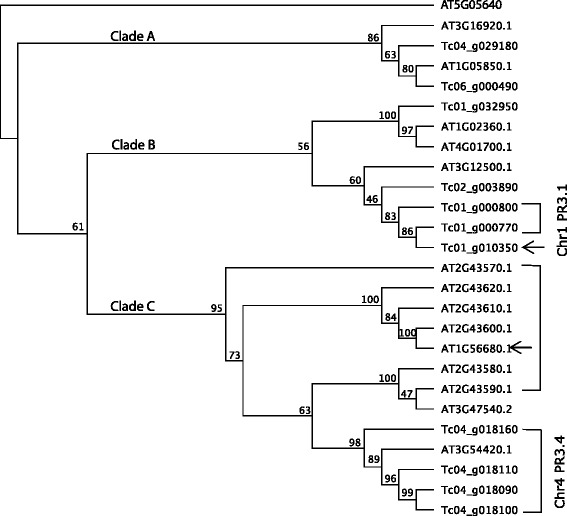
Maximum-likelihood phylogeny of Criollo and Arabidopsis PR-3 family members. Node labels represent bootstrap support from 100 replicates. Brackets denote members of tandem arrays. Arrows indicate cases where non-tandem array members group most-closely with a tandem array member. Branch lengths represent genetic distance in substitutions per site. AT5G05460, a cytosolic beta-endo-N-acetyglucosaminidase and member of the chitinase superfamily, was included as an outgroup

To investigate this phenomenon, we created maximum-likelihood trees for the PR-3 family (Fig. 3), the PR-1 family (Additional file 10: Figure S2, and the PR-4 family (Additional file 11: Figure S3), which include the gene family members from cacao and *Arabidopsis thaliana*. The phylogeny has several well-supported nodes indicating multiple PR-3 family members existed when Arabidopsis and cacao diverged. Further, the support for the tree suggests that there are three clades within the family. Cacao has tandem arrays in both clades B and C. Bootstrap support in clade B, interestingly, suggests that Tc01_g000770 is more closely related to Tc01_g010350 than it is to its tandem array members, Tc01_g000800. This suggests that in this scenario, a duplication led to the formation of an additional chitinase gene at the distal end of chromosome 1 after the tandem array had formed. Clade C contains tandem arrays of cacao and Arabidopsis genes. The branch support suggests that members of the Arabidopsis tandem array have continually expanded and diverged over evolutionary time, with strong support for array members split between three subclades. AT1G56690 presents another likely case of a recent non-local duplication, this one to a different chromosome. A fourth subclade contains the four members of the cacao tandem array on chromosome 4, none of which have been involved in recent duplications to other chromosomes. Examination of the PR-1 and PR-4 phylogenies also show evidence for expansion of gene families over evolutionary time locally, distally on chromosomes, and across chromosomes. Additional file 12: Table S10, Additional file 13: Table S11, Additional file 14: Table S12 include matrices of percentage identity for these three PR families, and further demonstrate that tandem array members are often, but not always, most closely related to one another.

### Induction of cacao PR gene expression by pathogen colonization

**Fig. 4:**
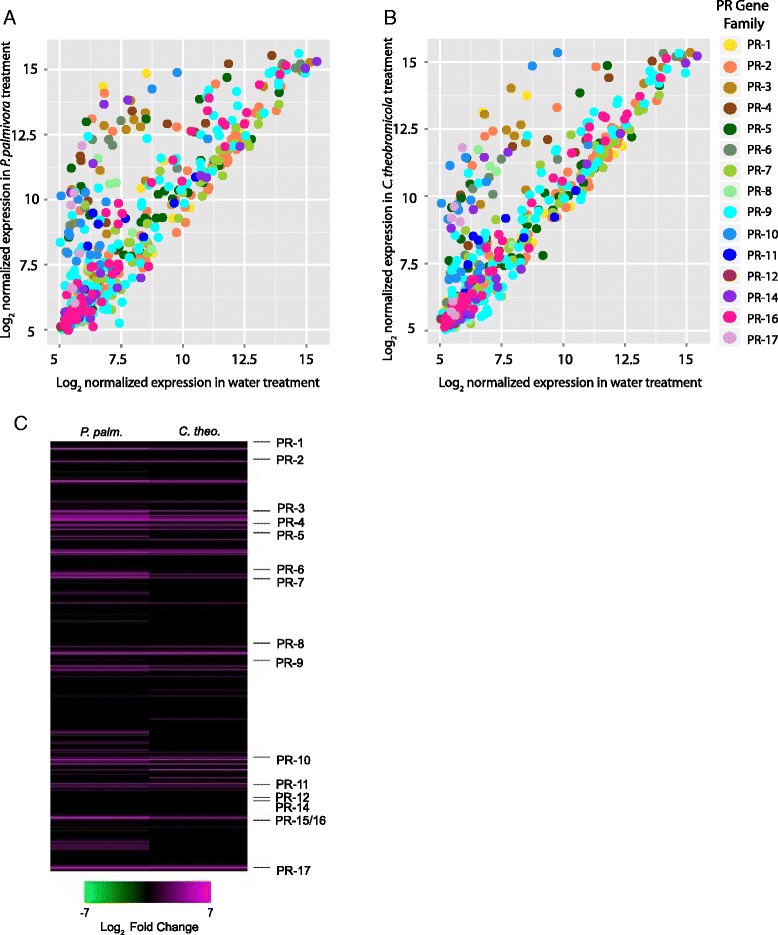
Microarray analysis of pathogen treatment on cacao PR gene expression. Scatterplots of normalized expression value for all probes for PR genes, comparing **a** *P. palmivora* treatment and water-treated control and **b** *C. theobromicola* with water-treated control. **c** Heatmap showing fold change in transcript abundance after pathogen treatments compared to water-treated control for all 359 Criollo PR genes. Black bars correspond to genes with non-significant (Benjamini-Hochberg *p* > 0.05) fold change or genes removed from analysis in background filtration

To further our understanding of PR gene expression in cacao, we measured global gene expression after treating plants with two pathogens, *P. palmivora* and *C. theobromicola*. Figure 4a and b show scatterplots of log_2_ normalized expression for *P. palmivora* and *C. theobromicola* treatment, respectively, compared to water treatment for all probes corresponding to PR genes on a whole genome microarray, revealing that normalized expression values detected by the microarray reflect transcript abundance ranging from very low to very high (Additional file 15: Table S13) in all treatments. As expected, a similar trend was noted when analyzing all probes on the microarray (Additional file 16: Figure S4). For both pathogens, the majority of PR gene probes revealed constitutive expression across treatments, a large number of genes being up-regulated in pathogen-treated samples, and only a few examples of PR gene down-regulation. A total of 67 PR genes were induced by *P. palmivora* and 45 were induced by *C. theobromicola* (Benjamini-Hochberg-corrected *p* < 0.05 [40]) (Table 3). Of the two pathogen treatments, *P. palmivora* had a stronger effect in that in generally induced more genes per family and the increase in transcript abundance relative to water-treated samples was greater (Fig. 4c, Additional file 17: Table S14). One exception was the PR-10s; while more of the PR-10 genes were induced by *P. palmivora*, those induced by both pathogens were equally or more strongly induced by *C. theobromicola.* A single PR-10 gene (Tc04_g028940) was strongly induced by *C. theobromicola* (log_2_ 3.6- fold increase) but not induced by *P. palmivora*. For both pathogens, statistically significant PR gene down-regulation was rare, as only 7 genes (2 PR-2 s, 3 PR-7 s, 1 PR-9, and 1 PR-16) were repressed by *P. palmivora* and none were by *C. theobromicola*. There was also significant overlap in genes differentially regulated by the two pathogens. Forty two PR genes were affected by both treatments, 32 were uniquely affected by *P. palmivora*, and 3 were unique to *C. theobromicola*. A large set of PR genes (159 in P. palmivora-treated samples and 188 in C. theobromicola-treated samples) were found to be expressed at similar levels in water and in pathogen treated tissues, suggesting that these genes may encode a set of proteins involved in basal defense in cacao, or they could be specifically induced in other tissues.

**Table 3:** Regulation of Criollo PR genes as detected by microarray

		*P. palmivora*		*C. theobromicola*	
	Number removed in background filtration (Average Log_2_ Normalized Expression <6)	Up-regulated	Down-regulated	Up-regulated	Down-regulated
PR-1	7/14	1/14	0/14	1/14	0/14
PR-2	11/43	5/43	2/43	4/43	0/43
PR-3	1/11	8/11	0/11	5/11	0/11
PR-4	1/8	3/8	0/8	3/8	0/8
PR-5	6/30	6/30	0/30	5/30	0/30
PR-6	2/8	5/8	0/8	2/8	0/8
PR-7	21/54	2/54	3/54	1/54	0/54
PR-8	9/14	2/14	0/14	2/14	0/14
PR-9	26/81	12/81	1/81	7/81	0/81
PR-10	13/23	8/23	0/23	6/23	0/23
PR-11	5/11	3/11	0/11	3/11	0/11
PR-12	3/3	0/3	0/3	0/3	0/3
PR-14	3/16	2/16	0/16	2/16	0/16
PR-16	16/38	7/38	1/38	1/38	0/38
PR-17	2/5	3/5	0/5	3/5	0/5
Total	126/359	67/359	7/359	45/359	0/359

Counts of up- and down-regulated genes represent the number of genes with Benjamini-Hochberg *p* < 0.05

### qRT-PCR validation of microarray results

To support the findings of our microarray analysis, we performed qRT-PCR on select genes from three families. Because family members, and tandem array members in particular, often have high similarity, with this analysis we sought to verify specificity of microarray probes, as well as to confirm induction of genes of interest. Our analysis included 30 genes: 14 PR-1 s, 6 PR-3 s, 7 PR-4 s and 3 PR-10s (Table 4). Primer sequences for qRT-PCR are listed in Additional file 18: Table S15 Generally the qRT-PCR results verified the induction of genes with statistically significant induction detected on the microarray, although the degree of induction was often underestimated by microarray measurement, as is often observed. By designing highly specific qRT-PCR primers, we were able to verify induction of multiple gene family members, and even tandem array members, in the PR-3 and PR-4 families. Members of a single array showed induction ranging from ~8 -fold to 5000-fold. Of the tested PR-10s, all verified the trend of equally strong induction by the two pathogens or greater induction by *C. theobromicola*.

**Table 4:** Validation of PR gene induction by qRT-PCR

		*P. palmivora* treatment		*C. theobromicola* treatment	
	Gene ID	Microarray fold induction	qRT-PCR fold induction	Microarray fold induction	qRT-PCR fold induction
PR-1 s	Tc01_g003940	N.S.	Transcript not detected	N.S.	Transcript not detected
	Tc01_g034430	N.S.	N.S.	N.S.	N.S.
	Tc02_g002380	N.S.	N.S.	N.S.	N.S.
	Tc02_g002390	N.S.	N.S.	N.S.	N.S.
	Tc02_g002400	N.S.	N.S.	N.S.	8.3 (*p* = .001)
	Tc02_g002410	125.4	763 (*p* < .001)	91.3	55.7 (*p* < .001)
	Tc02_g002420	N.S.	N.S.	N.S.	N.S.
	Tc02_g002430	N.S.	N.S.	N.S.	N.S.
	Tc02_g010380	N.S.	N.S.	N.S.	N.S.
	Tc05_g005530	N.S.	N.S.	N.S.	N.S.
	Tc09_g000720	N.S.	N.S.	N.S.	N.S.
	Tc09_g016580	N.S.	N.S.	N.S.	N.S.
	Tc09_g016590	N.S.	N.S.	N.S.	N.S.
	Tc10_g000980	N.S.	Transcript not detected	N.S.	Transcript not detected
PR-3 s	Tc01_g000770	33.6	70.2 (*p* < .001)	18.8	13.8 (*p* = .01)
	Tc02_g003890	27.1	Transcript not detected	22.31	Transcript not detected
	Tc04_g018100	22.5	5086.0 (*p* < .001)	8.3	36.7 (*p* = .019)
	Tc04_g018110	29.2	763.2 (*p* < .000)	11.5	13.7 (*p* = .041)
	Tc04_g018160	63.6	158.4 (*p* = .003)	73	65.6 (*p* = .001)
	Tc06_g000490	N.S.	3.4 (*p* = .016)	N.S	N.S.
PR-4 s	Tc00_g012980	N.S.	N.S.	N.S.	N.S.
	Tc05_g027210	24.9	1027.7 (*p* < .001)	14.9	22.7 (*p* = .01)
	Tc05_g027220	11.1	258.9 (*p* = .001)	6.7	N.S.
	Tc05_g027230	N.S.	164.1 (*p* = .011)	N.S.	N.S.
	Tc05_g027250	N.S.	N.S.	N.S.	N.S.
	Tc05_g027320	53.4	29.3 (*p* = .009)	17.8	8.9 (*p* = .001)
	Tc10_g011130	N.S.	61.5 (*p* < .001)	N.S.	N.S.
PR-10s	Tc01_g031100	39.7	28.0 (*p* = .019)	57.2	32.3 (*p* = .002)
	Tc04_g028780	25.5	32.9 (*p* = .027)	25.5	41.6 (*p* = .004)
	Tc04_g028860	6.02	24.3 (*p* = .038)	53.4	96.8 (*p* = .001)

Genes shown as induced by microarray had BH *p*-values < 0.05. N.S. indicates *p*-values for fold change were > 0.05. Inductions detected by qRT-PCR were calculated using REST software [96] and represent the average of five pathogen-treated samples compared to five water-treated samples relative to TcTub1 (Tc06_g000360). Transcripts were considered undetected if the average C_t_ value across all treatments was greater than 35

## Discussion

The role of PR genes in mediating resistance to disease has been well studied in a wide variety of model and crop plant species [11, 41–43]. These proteins are grouped together based on their increased accumulation in response to activation of systemic acquired resistance pathways and their roles in plant defense. Our analysis of the PR gene families of *T. cacao* resulted in the identification of multigene families for 15 families of PR proteins. These gene families include about 350 genes that are distributed throughout the genome. About 50 % of the cacao PR genes are found in arrays of tandemly duplicated genes, and many family members, even within tandem arrays, exhibited varying levels of inducibility by pathogen treatment. The structure of the PR gene families of five other plant species shared these features with cacao, suggesting that PR tandem arrays are features highly conserved within most if not all higher plants. The high degree of correlation in family sizes suggests that similar evolutionary forces have likely acted on diverse plant genera, likely indicating that PR family expansions have been beneficial to land plant survival. This body of work provides strong evidence that gene duplication and neo-functionalization, particularly with regard to expression dynamics, have played major roles in shaping the genomics of the plant defense response.

Local duplications arise through various mechanisms including polymerase slippage, unequal crossing over, and transposon movement, and local duplications are known to contribute to eukaryotic evolution by increasing genetic diversity [37, 44]. Organization of PR genes into tandem arrays has been described for several plants and PR families, including PR-7 s in tomato [45], PR-10s in grape [46], PR-12 s in Arabidopsis [47], and PR-1 s in Arabidopsis and rice [11], and PR-16 s in rice [48]. The physical clustering of PR-4 s in cacao was also previously described [19]. Tandem duplications have also been shown to play a key role in evolution of Resistance (R) gene families [49, 50] and they are particularly common in the NBS-LRR class of R genes, as well as in PR-1 s, thaumatins, germins, and major latex proteins in Arabidopsis [51]. Here we demonstrate that this clustering is common across PR families. Correlation analysis of family size indicates that sizes are similar across diverse plant taxa, indicating that expanded family sizes are common and are likely selectively beneficial in higher plants. Our phylogenetic analysis of the PR-1, PR-3, and PR-4 families suggests that the families have continually expanded both locally and inter-chromosomally over land plant evolution, although further investigation of expansions of certain sub-clades in different species is necessary to explain functional dynamics of family expansion.

Gene family expansions have a complicated interplay with expression dynamics. Employing our microarray analyses, unique expression dynamics within groups of family members with very high percent identity. The data presented here suggest that in some cases single genes within tandem arrays are induced by a given pathogen, while in other tandem arrays two or more genes can be induced by the same stimulus. Large tandem arrays for PR-10s (Chr4PR-10.6, 15 members) and PR-16 s (Chr5PR-16.3, 14 members) have members ranging from constitutive low expression to constitutive high expression, with a few showing inducibility by pathogens. Consequently, evolutionary dynamics of family members after a duplication event remain unclear, but several mechanisms are likely at play in a scenario-specific manner. First, selection could favor greater concentration of antimicrobial peptides produced in a given tissue, leading to multiple family members exhibiting similar protein structure and expression patterns. Our microarray analyses revealed several cases that could support this model; for example four PR-3 s that make up a tandem array were all induced by *P. palmivora*. Alternatively, mutations affecting nearby regulatory machinery or the coding sequence of the gene could result in new tissue specificity or binding/enzymatic activity of a protein. Our microarray dataset found that only one of six PR-1 s in a tandem array was induced by pathogen, suggesting the others have alternative functions, tissue specificities, or are in the process of becoming pseudogenes. Evolutionary studies have revealed that products of small-scale duplications diverge in expression more rapidly than they do in terms of protein structure [52] ,with age of paralogs correlating with their divergence in expression in Arabidopsis [53, 54] and rice [55]. For defense genes, divergence in expression patterns could be beneficial, decreasing metabolic burden associated with mounting a defense response in tissues distal to the site of infection. Further work, particularly RNA-seq experiments across a wide range of tissue types, would allow more comprehensive dissection of functional patterns associated with this gene organization. *In silico* promoter analysis may be a means of identifying a mechanism underlying expression dynamics of tandem arrays.

Teixeira et al. [22] previously reported the induction of more than 67 PR genes after infection of cacao plants with *Moniliophthora perniciosa*, but that the induction did not eliminate pathogen colonization. Similarly, the induction that we see here did not halt infection, but likely slow the pathogens’ progress. These transcriptomic experiments identify candidate genes that require functional characterization to better understand roles of PR proteins against the diversity of cacao’s pathogens. The infection and microarray analysis we performed with oomycete (*P. palmivora*) and fungal (*C. theobromicola*) pathogens confirms the induction of 67 and 45 PR genes by the respective pathogen treatments. However, the majority of the PR genes had stable expression across treatments under our experimental conditions. Analysis of other tissues may reveal that a subset of those genes have tissue specificity in their basal expression and inducibility. The existence of PR family members with constitutively high expression could suggest that certain family members have evolved to act as a preliminary line of defense. For example, two PR-3 s (Tc06_g000490 and Tc04_g029180) had very high expression in water treated samples. Constitutive high-level expression in leaves may allow the plant to begin degrading chitin of invading pathogens before PAMP or R-gene mediated signal transduction can elevate expression of induced defenses. Knockdown or deletion of these constitutive high-expressors followed by pathogen challenge would demonstrate the role of basal defense components. Broadly, we saw a more dramatic defense response in samples infected with *P. palmivora* than in those infected with *C. theobromicola,* with more genes being up-regulated and their degree of induction being greater. The microarray and qRT-PCR analysis indicated that the PR-10 family deviates from this trend, with members showing equal or more dramatic induction by *C. theobromicola* than by *P. palmivora.* The PR-10 member Tc04_g028860 is particularly noteworthy, showing 96-fold induction by *C. theobromicola* treatment, about four times the induction by *P. palmivora* treatment. While it is possible that these differences reflect pathogen-specific responses, we cannot rule out the possibility they result from different speeds with which the two pathogens colonize the host.

Induction of PR-1 genes is a hallmark of plant defense activation. While they belong to the well-studied Sperm Coating Protein/Tpx-1/Ag5/PR-1/Sc7 (SCP/TAPS) [56], a sub-group of the Cysteine-rich secretory protein superfamily, little is known about their biological function [57]. Our analysis indicates that TcPR1-g (Tc10_g000980) that was previously reported to be induced in tissue infected with witches’ broom [16], was not induced under our experimental conditions. This lack of induction by *P. palmivora* and *C. theobromicola* suggests that family member activation may differ for certain pathogens. Another example is the induction of the PR-1 Tc02_g002410, which was not induced by witches’ broom, by *P. palmivora* and *C. theobromicola*. Our qRT-PCR experiment validated strong induction of only this gene (>700 fold by P. palmivora and > 50 fold by *C. theobromicola*), and confirmed low expression of Tc10_g000980 across all samples. The specificity of the reaction is interesting, but even more puzzling as the function of PR-1 s in plants remains unclear.

PR-3 family member expression was also of particular interest because of our prior work with a class I chitinase (Tc02_g003890) [17]. Here we report induction of several other PR-3 s. A tandem array on chromosome four (Chr4PR-3.4) was notable in that multiple members were found to be induced by both pathogens, suggesting that, in this case, proximity may be contributing to their co-expression, and that these proteins may act in a coordinated fashion to defend the plant against both of the tested pathogens. While chitin is significantly less abundant in the cell walls of oomycetes than fungi, and its function in oomycetes is not well understood, recent evidence suggests that chitin synthase enzymes are active in hyphal tips, where chitin may play a role in cell wall structure [58]. Further, inhibition of these chitin synthases with nikkomycin Z led to bursting of hyphal tips and cell death. Accordingly, induction of chitinases in plants by oomycete treatment may reflect an important defense process, inhibition of hyphal tip growth.

Interestingly, our earlier work described that stable overexpression of Tc02_g003890, a class I chitinase, in transgenic cacao plants resulted in an increased resistance of leaves to *Colletotrichum gloeosporioides* [17]. The same gene was also upregulated in the highly disease-susceptible genotype ICS1 by treating leaves with salicylic acid [23], and we found that its transient overexpression in cacao leaves increases resistance to *P. capsici* [18]. The qRT-PCR we performed here did not verify its induction by treatment with *P. palmivora* or *C. theobromicola*, suggesting that this gene may respond to SA but not these two pathogens. This result suggests that the underlying mechanisms of these plant pathogen interactions are complex and that further research is necessary to unravel the specific mechanisms involved. One possibility is that the pathogens are able to suppress the mechanisms of SA induced gene expression via secretion of pathogen effector proteins as has been seen with other systems [59].

Cacao PR-4s were also recently identified [19] Pereira-Menezes et al.’s [19] work built upon an earlier EST database [25] by characterizing genotype specificity in the speed and level of induction of PR-4b (Tc05_g027210), which shows anti-fungal activity dependent on its RNase activity, in a resistant (TSH1188) and a susceptible (Catongo) genotype. Our microarray and qRT-PCR indicates that the gene was also induced by *P. palmivora* (more than 1000-fold and *C. theobromicola* (roughly 20-fold)*,* showing one of the strongest inductions of the genes tested with qRT-PCR. Its induction by a variety of pathogens makes it a critical candidate for further study. Analyses similar to Pereira-Menezes et al.’s work across a broader background of genotypes are required to validate the importance of genes described here. Assaying the effect of over-expression or knockout of this gene would be useful for defining roles of single genes within these families.

We observed a few differences in organization when comparing two different varieties of cacao. The two varieties compared in this study are representatives of distinct genetic clusters that developed over *T. cacao*’s evolution and are thought to have diverged because of the presence of geological barriers [31]. Consequently, it is possible that these two genotypes, having been subjected to different pathogens over their evolutionary history and having unique selective pressures applied by domestication after cultivation of cacao began, have undergone unique duplications or translocations altering gene organization. Indeed, our identification of PR genes in the two genomes may support this hypothesis, as gene counts within families differ for the two genomes, and while the positions of the genes are generally consistent, some chromosomal rearrangement appears to have occurred. It is possible however, that these are differences resulting from genome assembly strategies. Analysis of additional cacao genome sequences from other genetic groups [31] would help resolve these possibilities.

As induction of PR genes is a hallmark of the defense response in many plant species, their identification in cacao is critical to the study of cacao’s defense response. Our finding that PR gene family size and organization into tandem arrays is consistent across diverse plant species suggests that the diverse expression patterns seen within families in other species are likely similar to those we have described in cacao. Therefore, this study lays a foundational knowledge of defense gene expression upon which functional molecular genetic approaches can be based. Genes identified here, once functionally verified, will be useful in the breeding cacao cultivars with superior resistance to pathogens.

## Conclusions

In this study we identified 359 PR genes in the cacao genome, and found that approximately half of these physically cluster into tandem arrays with other members of the same PR family. Physical clustering of PR genes into tandem arrays was also identified in five diverse plant species. Using a whole genome microarray and qRT-PCR to measure the induction of genes by two cacao pathogens, we identified which PR genes are induced in leaf tissue by pathogens, and we identified differences in basal expression within PR families. This work is critical in improving the understanding of the defense response in cacao, and it provides a list of key candidate defense genes that will be the focus of future molecular characterization.

## Methods

### *Theobroma cacao* PR gene identification and filtration

Amino acid sequences for the type members of each PR gene family (Additional file 19: Table S1) were used as queries to search the Criollo genome database using BLASTp (cutoff E < 1e^−5^, BLOSUM62 matrix) [60]. Using this strategy, we identified putative genes in 15 of the 17 known plant PR protein classes. PR-13 s were not identified in the Criollo genome (they are specific to monocots and a subset of dicots [61]), and PR-15 s are also considered to be monocot specific, although the BLASTp search finds them in the Criollo genome because of their homology with PR-16 s [62]. Next, a custom Python (python.org) [63] script (PRAminoacidgetterASF) was used to extract protein IDs from the BLASTp output and use them to extract the peptide sequences available in the Criollo cacao genome database.

The list of amino acid sequences was uploaded to the NCBI Batch Web CD-Search Tool (v3.13) [64] with an e-value cutoff of 0.01. Another script (PRdomainsorterASF) was used to sort the output of the CD-Search with gene IDs and BLASTp E-values of putative PR genes. Polypeptides were manually curated for the presence of domains used in Wanderly-Nogueira et al. [43] to classify each family. For the PR-6 family, we used presence of the “potato-inhibitor family domain” (pfam00280) to screen putative cacao PR genes, as it is the only domain found in the type member sequence. Putative PR genes missing the characteristic domains were removed, and the remaining genes are listed in Additional file 1: Table S2.

This process was repeated for the Matina cacao genome [14]. In order to compare PR gene distribution in the genomes, a third python script was used to retrieve positional information from the Criollo and Matina GFF files (PRstartstopfinderASF). This data was plotted in Fig. 1 (Criollo) and Additional file 2: Figure S1 (Matina) using the R packages ggplot2 [65] and ggbio [66], and gene positional information is also included in Additional file 1: Table S2 (Criollo) and Additional file 3: Table S3 (Matina). All python scripts are available on the Guiltinan-Maximova Lab website (http://plantscience.psu.edu/research/labs/guiltinan/protocols/bioinformatic-scripts).

### PR gene identification in other plant species

Using the same type member queries, BLASTp searches were against predicted polypeptide sequences downloaded from Phytozome v10.3 (Goodstein et al., 2012) from the *Arabidopsis thaliana* (TAIR10), *Brachypodium distachyon* (v3.1), *Oryza sativa* (v7.0), *Populus trichocarpa* (v3.0), and *Vitis vinifera* (Genoscope 12×) genomes using the same parameters. The procedure described above was used to curate, use CD-Search, and organize PR genes in order to count the number of genes per class. Tandem arrays were manually identified using JBrowse [39] in Phytozome v10.3 [67]. For all species, the PR-15 and PR-16 lists were largely redundant because of homology of the families, but PR-15 s are monocot specific and should therefore only be present in *Brachypodium distachyon* and *Oryza sativa*. Therefore, for plotting gene family sizes in Fig. 2, these two families were combined. Gene IDs and BLASTp e-values for identified genes for these species are listed in Additional file 4: Table S4, Additional file 5: Table S5, Additional file 6: Table S6, Additional file 7: Table S7, Additional file 8: Table S8.

### Building PR-1, PR-3 and PR-4 phylogenies

To construct phylogenies, nucleotide sequences of family members for PR-1, PR-3, and PR-4 from the Criollo genome and primary transcripts from Arabidopsis (TAIR10) [32] were aligned using the MUSCLE [68] translational alignment function in Geneious [69] with eight iterations. Alignments were manually curated. No adjustments were made to the PR-1 or PR-3 families, but Tc05_g027340 was removed from the PR-4 alignment as it appears to have annotation errors in intron prediction. Maximum likelihood trees were generated in Geneious using a RAxML [70] plugin.

### Plant growth, infection, and RNA extraction

The seeds used for generating the plants for the experiment were collected under Panamanian Authority of the Environment (ANAM) scientific permit SE/AH-1-11. Seeds from open pollinated *T. cacao* mother trees, accession UF12, were collected from a plantation in Charagre, Bocas del Toro province, Panama. The seeds were surface sterilized by immersing them in 0.5 % sodium hypochlorite for three minutes and rinsed with sterile water before being placed for germination in plastic trays with soil (2:1 mixture of clay rich soil from Barro Colorado Island, Panama and rinsed river sand) and incubated in Percival growth chambers. One-month-old seedlings were transplanted to individual pots (600 ml volume) containing the same soil mixture and kept in the growth chambers. Germination of seeds and seedling growth was done in growth chambers (model I35LL, 115 volts, 1/4 Hp, series: 8503122.16, Percival Scientific, Inc., Perry IA) with 12/12 h light/dark photoperiod and temperatures of 30 °C and 26 °C respectively [71].

Two month old seedlings, with approximately six leaves each, were spray-inoculated with conidia of *Colletotrichum theobromicola* or zoospores of *Phytophthora palmivora*. Conidia of *C. theobromicola* were produced using the same methods as in [71] for production of other species of *Colletotrichum* and zoospores were produced as in [72]. Whole seedlings were sprayed either with pathogen inoculum (*P. palmivora* isolate PTP zoospores at 5 × 10^4^ per ml or *C. theobromicola* isolate ER08-11 conidia at 2 × 10^7^ per ml) or sterile distilled water (controls) and then placed back into the growing chamber, but only leaves in stage C [73] at the time of inoculation were considered as a target for the experiment. Pathogens *C. theobromicola* and *P. palmivora* were re-isolated from lesions developed in inoculated Samples were harvested from 72 h post-inoculation for RNA extraction, and tissue at this time point was used to re-isolate pathogen, which was considered as a measure of successful inoculation. Leaves sprayed with water remained healthy, did not develop lesions, and no pathogens were re-isolated from them. Representative photographs of infected and control leaves are shown in Additional file 20: Figure S5. Four seedlings received each treatment, and five leaf samples were collected from each group of four seedlings. Each biological replicate consisted of a single individual leaf. Target leaves were cut with scissors from the plant, immediately weighed, and placed in RNAlater solution in borosilicate vials following manufacturer’s instructions (Applied Biosystems/Ambion, Austin, TX). Vials containing samples were shipped to PSU on dry ice where RNA extractions were performed using a previously described protocol [74]. Total RNA sample concentration and purity was assessed using a NanoDrop spectrophotometer and RNA quality was determined using an Agilent Bioanalzyer.

### Microarray analysis

Transcriptomic analysis was performed using a whole-genome Roche NimbleGen custom oligo expression array (platform GPL18356), which was previously described in [75]. Probe labeling, hybridization, and detection were performed at the Penn State Genomics Core Facility, and the statistical analysis of the microarray data were performed as previously described [75].Briefly, the Bioconductor package [76] was used in R to perform quality control checks and calculate normalized expression values using the RMA procedure. Normalized expression values were plotted to ensure all replicates for a given treatment had similar expression patterns. These data are available on GEO (GSE73804). In calculating fold induction, probes with mean log_2_ expression values across all probes less than 6 were removed. The LIMMA package [77, 78] was then used to calculate fold induction on a per-probe basis and to calculate a Bayesian moderated test statistic for each comparison (pathogen-treatments relative to water-treatment). A Benjamini-Hochberg multiple testing correction [40] was then applied. Probes with Benjamini-Hochberg *p* < 0.05 were considered significant. In identifying individual PR genes with statistically significant differential regulation, any gene with multiple probes showing statistically significant change had fold change recalculated by averaging across all significant probes.

### CDNA synthesis and qRT-PCR validation of microarray

One microgram of RNA from each of the five samples from each treatment were reverse transcribed by M-MuLV Reverse Transcriptase (New England Biolabs, Ipswich, MA, USA) with oligo-(dT)_15_ primers to obtain cDNA. To create highly specific primers for PR gene family members, nucleotide sequences of for the PR-1, PR-3, PR-4, and PR-10 families were aligned using MUSCLE [72] in Geneious [73]. qRT-PCR primers were designed to target bases that differentiate family members. Primer sequences are listed in Additional file 18: Table S15. qRT-PCR was performed in a total reaction volume of 10 μL containing 4 μL of diluted cDNA (1:8), 5 μL of SYBR Green PCR Master Mix (TaKaRa, Mountain View, CA, USA), 0.2 μL of Rox and 0.4 μL of each 5 μM primer. Each reaction was performed on each of the five samples per treatment in technical duplicate using the Applied Biosystem Step One Plus Realtime PCR System (Nutley, NJ, USA) with the following program: 15 min at 94 °C, 40 cycles of 15 s at 94 °C, 20 s at 60 °C, and 40 s at 72 °C. The specificity of the primer pair was verified by dissociation curve.

Data normalization, a statistical randomization test, and relative pathogen-treated vs. water-treated expression ratios were computed using REST [64]. Fold changes with *p-values* less than 0.05 were considered significant.

## Ethics approval

As the study did not include any human or animal participants, no ethics approval was required.

## Consent to publish

As no human participants were involved in the study, no consent was required.

## Availability of data

Microarray data are available at NCBI (GEO: GSE73804). The Criollo cacao genome is available at http://cocoagendb.cirad.fr/ and the Matina cacao genome, *A. thaliana*, *B. distachyon*, *O. sativa*, *P. trichocarpa*, and *V. vinifera* genomes are accessible through Phytozome.

## Additional files

Additional file 1: Table S2. Gene IDs and positions of Criollo PR genes mapped to the ten cacao chromosomes. Those not mapped to the ten chromosomes are appended to the bottom of the list without positional information. (PDF 4169 kb) Additional file 2: Figure S1. Karyogram depicting the position of PR genes along the length of chromosomes based on the Matina genome sequence. Due to resolution of the image lines representing nearby genes partially overlap. (PDF 4169 kb) Additional file 3: Table S3. Gene IDs and positions of Matina PR genes mapped to the ten cacao chromosomes. Those not mapped to the ten chromosomes are appended to the bottom of the list without positional information. (PDF 4169 kb) Additional file 4: Table S4. Gene IDs and BLASTp E-values for *Arabidopsis thaliana* PR loci. (PDF 4169 kb) Additional file 5: Table S5. Gene IDs and BLASTp E-values for *Brachypodium distachyon* PR loci. (PDF 4169 kb) Additional file 6: Table S6. Gene IDs and BLASTp E-values for *Oryza sativa* PR loci. (PDF 4169 kb) Additional file 7: Table S7. Gene IDs and BLASTp E-values for *Populus trichocarpa* PR loci. (PDF 4169 kb) Additional file 8: Table S8. Gene IDs and BLASTp E-values for *Vitis vinifera* PR loci. (PDF 4169 kb) Additional file 9: Table S9. Percentage of PR genes in tandem arrays in the six analyzed plant species. (PDF 4169 kb) Additional file 10: Figure S2. Maximum-likelihood phylogeny of Criollo and Arabidopsis PR-1 family members. (PDF 4169 kb) Additional file 11: Figure S3. Maximum-likelihood phylogeny of Criollo and Arabidopsis PR-4 family members. (PDF 4169 kb) Additional file 12: Table S10. Percent identities for Criollo PR-1 genes, color-coded to show tandem array members. (PDF 4169 kb) Additional file 13: Table S11. Percent identities for Criollo PR-3 genes, color-coded to show tandem array members (PDF 4169 kb) Additional file 14: Table S12. Percent identities for Criollo PR-4 genes, color-coded to show tandem array members. (PDF 4169 kb) Additional file 15: Table S13. Log_2_ normalized expression values for all PR genes on microarray, with values averaged across five biological replicates. (PDF 4168 kb) Additional file 16: Figure S4. Whole genome gene expression profiles in microarray-analyzed leaves. Scatterplots of log_2_ normalized expression values for all probes on the microarray, comparing pathogen treatments with water treatment. (PDF 4169 kb) Additional file 17: Table S14. Log_2_ fold change for all significantly regulated (Benjamini-Hochberg *p* < 0.05) PR genes on microarray. (PDF 4169 kb) Additional file 18: Table S15. Sequences of qRT-PCR primers for validation of PR-1, PR-3, and PR-4 family expression. (PDF 4169 kb) Additional file 19: Table S1. PR gene gamily type members: GenBank accession numbers for PR type member amino acid sequences used as BLASTp queries (PDF 4169 kb) Additional file 20: Figure S5. Representative photographs showing leaves 72 h after A) H_2_O, B) *C. theobromicola* (with red lines indicating developing lesions), and C) *P. palmivora* treatment. Scale bars represent 1 cm. (PDF 4169 kb)

## Abbreviations

PR: pathogenesis-related
qRT-PCR: quantitative real-time polymerase chain reaction
