# Erratum to: Theobroma cacao L. pathogenesis-related gene tandem array members show diverse expression dynamics in response to pathogen colonization

**DOI:** 10.1186/s12864-016-3073-8

**Published:** 2016-09-07

**Authors:** Andrew S. Fister, Luis C. Mejia, Yufan Zhang, Edward Allen Herre, Siela N. Maximova, Mark J. Guiltinan

**Affiliations:** 1 The Huck Institutes of the Life Sciences, The Pennsylvania State University, 422 Life Sciences Building, University Park, 16802 PA USA; 2 Institute for Scientific Research and High Technology Services (INDICASAT-AIP), Panama City, Panama; 3 Smithsonian Tropical Research Institute (STRI), Unit 9100, Box 0948, Balboa, Ancon, DPO AA 34002-9998 Panama; 4 Department of Electrical Engineering, Princeton University, Princeton, NJ 08544 USA; 5 The Department of Plant Science, The Pennsylvania State University, 422 Life Sciences Building, University Park, 16802 PA USA

## Erratum

The original version of the manuscript [1] contained an incorrectly named Criollo gene ID on chromosome 1 in the first sentence, under the subheading “Organization of PR gene families into tandem arrays”. The second gene on chromosome 1, Tc##_g######, should therefore be **Tc01_g000020**.
