# Supplementary material for: Theobroma cacao L. pathogenesis-related gene tandem array members show diverse expression dynamics in response to pathogen colonization
Source: BMC Genomics. 2016 May 17;17:363. doi: 10.1186/s12864-016-2693-3 (PMC4869279; doi:10.1186/s12864-016-2693-3)
Supplement: Additional file 1: Table S2. — Gene IDs and positions of Criollo PR genes mapped to the ten cacao chromosomes. Those not mapped to the ten chromosomes are appended to the bottom of the list without positional information. (PDF 4169 kb) [file 12864_2016_2693_MOESM1_ESM.pdf]

Supplemental Table S2 - PR gene positions in Criollo genome, color-coded by PR gene family					
**Chromosome**	**Start**	**Stop**	**PR Gene Family**	**Gene ID**	**Tandem Array ID**
Chr1	46446	50611	PR-7	Tc01_g000090	
Chr1	407076	408180	PR-3	Tc01_g000770	Chr1PR-3.1
Chr1	416020	417055	PR-3	Tc01_g000800	Chr1PR-3.1
Chr1	592960	594207	PR-9	Tc01_g001190	
Chr1	756862	758055	PR-5	Tc01_g001580	Chr1PR-5.2
Chr1	764592	765968	PR-5	Tc01_g001590	Chr1PR-5.2
Chr1	1223958	1225321	PR-9	Tc01_g002490	
Chr1	2046944	2047477	PR-1	Tc01_g003940	
Chr1	2390176	2391738	PR-9	Tc01_g004590	
Chr1	2797614	2800013	PR-7	Tc01_g005210	Chr1PR-7.3
Chr1	2801937	2804204	PR-7	Tc01_g005220	Chr1PR-7.3
Chr1	3352887	3355169	PR-7	Tc01_g006160	
Chr1	3427643	3429296	PR-9	Tc01_g006280	
Chr1	3517917	3519445	PR-2	Tc01_g006420	
Chr1	5006124	5007854	PR-9	Tc01_g008530	
Chr1	6168606	6170228	PR-2	Tc01_g010310	Chr1PR-2.4
Chr1	6172632	6176254	PR-2	Tc01_g010320	Chr1PR-2.4
Chr1	6221240	6222258	PR-3	Tc01_g010350	
Chr1	8521396	8522841	PR-9	Tc01_g013330	
Chr1	9077679	9080866	PR-2	Tc01_g014030	
Chr1	12365543	12367610	PR-2	Tc01_g017650	
Chr1	15266578	15268183	PR-2	Tc01_g021070	
Chr1	21684400	21686697	PR-7	Tc01_g026170	
Chr1	24449757	24451200	PR-9	Tc01_g029350	
Chr1	25614422	25615074	PR-10	Tc01_g031100	
Chr1	26238743	26239660	PR-8	Tc01_g032120	
Chr1	26379168	26379491	PR-8	Tc01_g032260	
Chr1	26567546	26568662	PR-9	Tc01_g032570	
Chr1	26825013	26825938	PR-3	Tc01_g032950	
Chr1	26960822	26961584	PR-16	Tc01_g033170	
Chr1	27725371	27726015	PR-1	Tc01_g034430	
Chr1	27788216	27788682	PR-14	Tc01_g034520	
Chr1	28094750	28095737	PR-8	Tc01_g035050	Chr1PR-8.5
Chr1	28098396	28099445	PR-8	Tc01_g035060	Chr1PR-8.5
Chr1	28136550	28137445	PR-8	Tc01_g035140	Chr1PR-8.5

Chr1	28139089	28139985	PR-8	Tc01_g035150	Chr1PR-8.5
Chr1	28141342	28142244	PR-8	Tc01_g035160	Chr1PR-8.5
Chr1	29089378	29096068	PR-7	Tc01_g037010	Chr1PR-7.6
Chr1	29099230	29102593	PR-7	Tc01_g037020	Chr1PR-7.6
Chr1	29106890	29110006	PR-7	Tc01_g037030	Chr1PR-7.6
Chr1	29115406	29119284	PR-7	Tc01_g037040	Chr1PR-7.6
Chr1	30267844	30268167	PR-14	Tc01_g039190	
Chr2	1453223	1453702	PR-1	Tc02_g002380	Chr2PR-1.1
Chr2	1454367	1454837	PR-1	Tc02_g002390	Chr2PR-1.1
Chr2	1456734	1457219	PR-1	Tc02_g002400	Chr2PR-1.1
Chr2	1460085	1460573	PR-1	Tc02_g002410	Chr2PR-1.1
Chr2	1463710	1464189	PR-1	Tc02_g002420	Chr2PR-1.1
Chr2	1465775	1466269	PR-1	Tc02_g002430	Chr2PR-1.1
Chr2	1782306	1783525	PR-5	Tc02_g003020	
Chr2	1834936	1836914	PR-9	Tc02_g003150	
Chr2	2204825	2205983	PR-3	Tc02_g003890	
Chr2	2838778	2841102	PR-7	Tc02_g005130	
Chr2	2891313	2892972	PR-5	Tc02_g005190	Chr2PR-5.2
Chr2	2900033	2902683	PR-5	Tc02_g005200	Chr2PR-5.2
Chr2	3567043	3567463	PR-12	Tc02_g006630	Chr2PR-12.3
Chr2	3574782	3574952	PR-12	Tc02_g006660	Chr2PR-12.3
Chr2	3791974	3796328	PR-2	Tc02_g007080	
Chr2	3911517	3916523	PR-7	Tc02_g007300	
Chr2	4604542	4606761	PR-9	Tc02_g008380	
Chr2	5265134	5265811	PR-17	Tc02_g009590	Chr2PR-17.4
Chr2	5268266	5269520	PR-17	Tc02_g009600	Chr2PR-17.4
Chr2	5272315	5273019	PR-17	Tc02_g009610	Chr2PR-17.4
Chr2	5280292	5280972	PR-17	Tc02_g009630	Chr2PR-17.4
Chr2	5290110	5290810	PR-17	Tc02_g009650	Chr2PR-17.4
Chr2	5714692	5716563	PR-1	Tc02_g010380	
Chr2	6680377	6681597	PR-9	Tc02_g011920	Chr2PR-9.5
Chr2	6688821	6690092	PR-9	Tc02_g011930	Chr2PR-9.5
Chr2	6692863	6694134	PR-9	Tc02_g011940	Chr2PR-9.5
Chr2	6698094	6699389	PR-9	Tc02_g011950	Chr2PR-9.5
Chr2	6701728	6702974	PR-9	Tc02_g011960	Chr2PR-9.5
Chr2	6713925	6715033	PR-9	Tc02_g011990	Chr2PR-9.5
Chr2	6725602	6726798	PR-9	Tc02_g012000	Chr2PR-9.5
Chr2	6728402	6732943	PR-9	Tc02_g012010	Chr2PR-9.5

Chr2	6733980	6735168	PR-9	Tc02_g012020	Chr2PR-9.5
Chr2	7596477	7598446	PR-9	Tc02_g013360	
Chr2	9964462	9964902	PR-14	Tc02_g016580	
Chr2	17976635	17977630	PR-9	Tc02_g022830	
Chr2	19074797	19075795	PR-2	Tc02_g023780	
Chr2	22789048	22793374	PR-2	Tc02_g028070	
Chr2	23779373	23780685	PR-9	Tc02_g029020	Chr2PR-9.6
Chr2	23812198	23813502	PR-9	Tc02_g029090	Chr2PR-9.6
Chr2	23817409	23818719	PR-9	Tc02_g029110	Chr2PR-9.6
Chr2	23827973	23829773	PR-9	Tc02_g029120	Chr2PR-9.6
Chr2	23839783	23845668	PR-9	Tc02_g029131	Chr2PR-9.6
Chr2	24935435	24936685	PR-9	Tc02_g030420	Chr2PR-9.7
Chr2	24943784	24945334	PR-9	Tc02_g030430	Chr2PR-9.7
Chr2	25295410	25295847	PR-9	Tc02_g030970	Chr2PR-9.8
Chr2	25297462	25298357	PR-9	Tc02_g030980	Chr2PR-9.8
Chr3	320042	321034	PR-9	Tc03_g000530	
Chr3	4837264	4838273	PR-5	Tc03_g005540	
Chr3	10318529	10319194	PR-16	Tc03_g009350	
Chr3	16215818	16223170	PR-16	Tc03_g016120	
Chr3	17331874	17332776	PR-8	Tc03_g017760	Chr3PR-8.1
Chr3	17340582	17341478	PR-8	Tc03_g017780	Chr3PR-8.1
Chr3	17344432	17345325	PR-8	Tc03_g017790	Chr3PR-8.1
Chr3	18014708	18017044	PR-7	Tc03_g018930	
Chr3	18236392	18237551	PR-9	Tc03_g019180	
Chr3	19476132	19478006	PR-2	Tc03_g021200	
Chr3	19763023	19766766	PR-7	Tc03_g021760	
Chr3	19837251	19838667	PR-9	Tc03_g021880	
Chr3	20186386	20189613	PR-7	Tc03_g022570	Chr3PR-7.2
Chr3	20192438	20196672	PR-7	Tc03_g022580	Chr3PR-7.2
Chr3	20198996	20202591	PR-7	Tc03_g022590	Chr3PR-7.2
Chr3	20236328	20237838	PR-2	Tc03_g022650	
Chr3	20812784	20814495	PR-9	Tc03_g023600	
Chr3	20873633	20874124	PR-14	Tc03_g023690	
Chr3	21230293	21255588	PR-7	Tc03_g024170	
Chr3	22569364	22573346	PR-7	Tc03_g026320	Chr3PR-7.3
Chr3	22574911	22577142	PR-7	Tc03_g026330	Chr3PR-7.3
Chr3	22578287	22580454	PR-7	Tc03_g026340	Chr3PR-7.3
Chr3	22705359	22708866	PR-7	Tc03_g026560	Chr3PR-7.4

Chr3	22710814	22714408	PR-7	Tc03_g026570	Chr3PR-7.4
Chr3	22978758	22979435	PR-5	Tc03_g026960	Chr3PR-5.5
Chr3	22989396	22989923	PR-5	Tc03_g026980	Chr3PR-5.5
Chr3	22995503	22996177	PR-5	Tc03_g026990	Chr3PR-5.5
Chr3	22999218	22999882	PR-5	Tc03_g027000	Chr3PR-5.5
Chr3	23001302	23001976	PR-5	Tc03_g027010	Chr3PR-5.5
Chr3	23003689	23004303	PR-5	Tc03_g027020	Chr3PR-5.5
Chr3	23004849	23005571	PR-5	Tc03_g027030	Chr3PR-5.5
Chr3	24496301	24497326	PR-2	Tc03_g029620	
Chr4	255387	256364	PR-9	Tc04_g000340	
Chr4	1012521	1013740	PR-8	Tc04_g001620	
Chr4	2788623	2790250	PR-9	Tc04_g004350	Chr4PR-9.1
Chr4	2791630	2793436	PR-9	Tc04_g004360	Chr4PR-9.1
Chr4	2794822	2796456	PR-9	Tc04_g004370	Chr4PR-9.1
Chr4	8043355	8044235	PR-5	Tc04_g008530	
Chr4	11658052	11661012	PR-2	Tc04_g012520	
Chr4	12331457	12331935	PR-14	Tc04_g013310	
Chr4	13347034	13347476	PR-5	Tc04_g014480	
Chr4	14722179	14723300	PR-9	Tc04_g016340	
Chr4	14745374	14745865	PR-14	Tc04_g016380	Chr4PR-14.2
Chr4	14765307	14765876	PR-14	Tc04_g016400	Chr4PR-14.2
Chr4	14785146	14786072	PR-14	Tc04_g016440	Chr4PR-14.2
Chr4	14799364	14799929	PR-14	Tc04_g016450	Chr4PR-14.2
Chr4	15012802	15018041	PR-9	Tc04_g016710	Chr4PR-9.3
Chr4	15033309	15034430	PR-9	Tc04_g016740	Chr4PR-9.3
Chr4	15054772	15055892	PR-9	Tc04_g016760	Chr4PR-9.3
Chr4	15937181	15938075	PR-3	Tc04_g018090	Chr4PR-3.4
Chr4	15942222	15943027	PR-3	Tc04_g018100	Chr4PR-3.4
Chr4	15951169	15952222	PR-3	Tc04_g018110	Chr4PR-3.4
Chr4	15995814	15996879	PR-3	Tc04_g018160	Chr4PR-3.4
Chr4	17148238	17151103	PR-9	Tc04_g019840	
Chr4	17484077	17485694	PR-2	Tc04_g020310	
Chr4	17879840	17881090	PR-5	Tc04_g020870	
Chr4	19580508	19585108	PR-2	Tc04_g023620	
Chr4	21631422	21632757	PR-9	Tc04_g027220	Chr4PR-9.5
Chr4	21636062	21637717	PR-9	Tc04_g027230	Chr4PR-9.5
Chr4	22135251	22136317	PR-9	Tc04_g028060	
Chr4	22480311	22480887	PR-10	Tc04_g028740	Chr4PR-10.6

Chr4	22481675	22482230	PR-10	Tc04_g028750	Chr4PR-10.6
Chr4	22483092	22483770	PR-10	Tc04_g028760	Chr4PR-10.6
Chr4	22489740	22490297	PR-10	Tc04_g028780	Chr4PR-10.6
Chr4	22492172	22492742	PR-10	Tc04_g028790	Chr4PR-10.6
Chr4	22494552	22495144	PR-10	Tc04_g028800	Chr4PR-10.6
Chr4	22499557	22500145	PR-10	Tc04_g028830	Chr4PR-10.6
Chr4	22501085	22501677	PR-10	Tc04_g028840	Chr4PR-10.6
Chr4	22504672	22505247	PR-10	Tc04_g028860	Chr4PR-10.6
Chr4	22506913	22507501	PR-10	Tc04_g028870	Chr4PR-10.6
Chr4	22508764	22509336	PR-10	Tc04_g028880	Chr4PR-10.6
Chr4	22511232	22511804	PR-10	Tc04_g028900	Chr4PR-10.6
Chr4	22515773	22516365	PR-10	Tc04_g028920	Chr4PR-10.6
Chr4	22517301	22517886	PR-10	Tc04_g028930	Chr4PR-10.6
Chr4	22518569	22519103	PR-10	Tc04_g028940	Chr4PR-10.6
Chr4	22641991	22643524	PR-3	Tc04_g029180	
Chr4	22709023	22712083	PR-2	Tc04_g029300	
Chr4	22833512	22834743	PR-9	Tc04_g029620	
Chr4	22935290	22936567	PR-9	Tc04_g029820	
Chr5	155638	156316	PR-10	Tc05_g000310	Chr5PR-10.1
Chr5	171073	171753	PR-10	Tc05_g000340	Chr5PR-10.1
Chr5	187696	188376	PR-10	Tc05_g000350	Chr5PR-10.1
Chr5	212396	215208	PR-10	Tc05_g000380	Chr5PR-10.1
Chr5	2252411	2253450	PR-5	Tc05_g004240	
Chr5	2957852	2958562	PR-1	Tc05_g005530	
Chr5	3297559	3300161	PR-9	Tc05_g006120	
Chr5	5445578	5446622	PR-16	Tc05_g008860	
Chr5	5854961	5858791	PR-16	Tc05_g009390	
Chr5	6655743	6659172	PR-7	Tc05_g010230	
Chr5	11319064	11321364	PR-7	Tc05_g013470	
Chr5	14642635	14643881	PR-2	Tc05_g016070	
Chr5	20017774	20018430	PR-6	Tc05_g022770	Chr5PR-6.2
Chr5	20026069	20026725	PR-6	Tc05_g022780	Chr5PR-6.2
Chr5	21264780	21265618	PR-16	Tc05_g024700	
Chr5	21455283	21456757	PR-16	Tc05_g024940	
Chr5	21700051	21700875	PR-16	Tc05_g025310	Chr5PR-16.3
Chr5	21705030	21705781	PR-16	Tc05_g025330	Chr5PR-16.3
Chr5	21713402	21714258	PR-16	Tc05_g025350	Chr5PR-16.3
Chr5	21715594	21718017	PR-16	Tc05_g025360	Chr5PR-16.3

Chr5	21737964	21738819	PR-16	Tc05_g025400	Chr5PR-16.3
Chr5	21740097	21740875	PR-16	Tc05_g025410	Chr5PR-16.3
Chr5	21750659	21751516	PR-16	Tc05_g025420	Chr5PR-16.3
Chr5	21761479	21762326	PR-16	Tc05_g025430	Chr5PR-16.3
Chr5	21771629	21772485	PR-16	Tc05_g025440	Chr5PR-16.3
Chr5	21775301	21776123	PR-16	Tc05_g025450	Chr5PR-16.3
Chr5	21793844	21795080	PR-16	Tc05_g025470	Chr5PR-16.3
Chr5	21796024	21800895	PR-16	Tc05_g025480	Chr5PR-16.3
Chr5	21850086	21852832	PR-16	Tc05_g025520	Chr5PR-16.3
Chr5	21855659	21859290	PR-16	Tc05_g025530	Chr5PR-16.3
Chr5	23011025	23011535	PR-4	Tc05_g027210	Chr5PR-4.4
Chr5	23015545	23016832	PR-4	Tc05_g027220	Chr5PR-4.4
Chr5	23022482	23023859	PR-4	Tc05_g027230	Chr5PR-4.4
Chr5	23030430	23031726	PR-4	Tc05_g027250	Chr5PR-4.4
Chr5	23076382	23077121	PR-4	Tc05_g027320	Chr5PR-4.4
Chr5	23082584	23089537	PR-4	Tc05_g027340	Chr5PR-4.4
Chr5	23647781	23650823	PR-2	Tc05_g028370	
Chr5	25358388	25359598	PR-9	Tc05_g031640	
Chr5	25533413	25534642	PR-16	Tc05_g031880	
Chr6	283670	284572	PR-16	Tc06_g000370	Chr6PR-16.1
Chr6	288415	289525	PR-16	Tc06_g000400	Chr6PR-16.1
Chr6	342951	345342	PR-3	Tc06_g000490	
Chr6	512464	520849	PR-7	Tc06_g000810	
Chr6	627416	627703	PR-14	Tc06_g000990	Chr6PR-14.2
Chr6	628183	628635	PR-14	Tc06_g001000	Chr6PR-14.2
Chr6	1190282	1191669	PR-2	Tc06_g001730	
Chr6	7792149	7794494	PR-9	Tc06_g008540	
Chr6	8665491	8668445	PR-16	Tc06_g009700	
Chr6	9373572	9375890	PR-7	Tc06_g010630	
Chr6	10708214	10709837	PR-2	Tc06_g012580	
Chr6	11247628	11253176	PR-7	Tc06_g013520	
Chr6	12106897	12108599	PR-9	Tc06_g014950	
Chr6	14479691	14481234	PR-9	Tc06_g019150	
Chr6	14587458	14590282	PR-2	Tc06_g019320	
Chr6	14715208	14716459	PR-9	Tc06_g019650	
Chr6	14788011	14791633	PR-7	Tc06_g019800	
Chr6	14899115	14899570	PR-14	Tc06_g020010	Chr6PR-14.3
Chr6	14904636	14905201	PR-14	Tc06_g020030	Chr6PR-14.3

Chr6	15375477	15378303	PR-7	Tc06_g021130	
Chr7	242337	248261	PR-7	Tc07_g000490	
Chr7	1373943	1375917	PR-2	Tc07_g002650	
Chr7	2469342	2470759	PR-16	Tc07_g004510	Chr7PR-16.1
Chr7	2541372	2545366	PR-16	Tc07_g004580	Chr7PR-16.1
Chr7	2656056	2656726	PR-16	Tc07_g004710	
Chr8	136530	138827	PR-7	Tc08_g000230	Chr8PR-7.1
Chr8	140672	142931	PR-7	Tc08_g000240	Chr8PR-7.1
Chr8	144535	148772	PR-7	Tc08_g000250	Chr8PR-7.1
Chr8	157014	159803	PR-7	Tc08_g000260	Chr8PR-7.1
Chr8	162515	164797	PR-7	Tc08_g000270	Chr8PR-7.1
Chr8	861589	862531	PR-5	Tc08_g001670	
Chr8	1035418	1037669	PR-2	Tc08_g001980	
Chr8	1251906	1252272	PR-12	Tc08_g002440	
Chr8	1392341	1393706	PR-9	Tc08_g002680	
Chr8	1982231	1984015	PR-5	Tc08_g003730	Chr8PR-5.2
Chr8	1994947	1996618	PR-5	Tc08_g003740	Chr8PR-5.2
Chr8	2135796	2139162	PR-7	Tc08_g004000	
Chr8	2171236	2172745	PR-9	Tc08_g004060	
Chr8	2812158	2813696	PR-2	Tc08_g005240	
Chr8	4454124	4457212	PR-7	Tc08_g007740	
Chr8	5838275	5839415	PR-2	Tc08_g009900	
Chr8	6072846	6074589	PR-5	Tc08_g010190	
Chr8	7977834	7979127	PR-9	Tc08_g012700	
Chr8	8542455	8545412	PR-9	Tc08_g013300	
Chr9	115915	118110	PR-2	Tc09_g000150	
Chr9	349534	350866	PR-9	Tc09_g000620	
Chr9	392587	393144	PR-1	Tc09_g000720	
Chr9	644365	645956	PR-9	Tc09_g001160	
Chr9	940710	942098	PR-11	Tc09_g001640	
Chr9	1746195	1747442	PR-11	Tc09_g003110	Chr9PR-11.1
Chr9	1748948	1752317	PR-11	Tc09_g003120	Chr9PR-11.1
Chr9	1754274	1755716	PR-11	Tc09_g003130	Chr9PR-11.1
Chr9	1756032	1759736	PR-11	Tc09_g003140	Chr9PR-11.1
Chr9	1761439	1764995	PR-11	Tc09_g003150	Chr9PR-11.1
Chr9	1765831	1767491	PR-11	Tc09_g003160	Chr9PR-11.1

Chr9	1771501	1774951	PR-11	Tc09_g003180	Chr9PR-11.1
Chr9	1784184	1785419	PR-11	Tc09_g003190	Chr9PR-11.1
Chr9	1786169	1787434	PR-11	Tc09_g003200	Chr9PR-11.1
Chr9	3455242	3457297	PR-2	Tc09_g006080	
Chr9	3924740	3927797	PR-7	Tc09_g006830	
Chr9	4056208	4056831	PR-16	Tc09_g007080	
Chr9	5106593	5109921	PR-2	Tc09_g008700	
Chr9	6108778	6111167	PR-2	Tc09_g010240	
Chr9	6842502	6843844	PR-9	Tc09_g011360	
Chr9	7056410	7061303	PR-2	Tc09_g011610	
Chr9	8542813	8545250	PR-2	Tc09_g013490	
Chr9	11250595	11251258	PR-5	Tc09_g016370	
Chr9	11463491	11464078	PR-1	Tc09_g016580	Chr9PR-1.2
Chr9	11465593	11466093	PR-1	Tc09_g016590	Chr9PR-1.2
Chr9	12561121	12565191	PR-9	Tc09_g017500	
Chr9	15718258	15718875	PR-16	Tc09_g019910	Chr9PR-16.3
Chr9	15720067	15720699	PR-16	Tc09_g019920	Chr9PR-16.3
Chr9	18149426	18155830	PR-2	Tc09_g021600	
Chr9	20183483	20184809	PR-2	Tc09_g023540	
Chr9	20776268	20777977	PR-2	Tc09_g024130	Chr9PR-2.4
Chr9	20788910	20789878	PR-2	Tc09_g024140	Chr9PR-2.4
Chr9	20791335	20792780	PR-2	Tc09_g024150	Chr9PR-2.4
Chr9	23914213	23916248	PR-7	Tc09_g028050	
Chr9	24349477	24353936	PR-7	Tc09_g028480	
Chr9	26293694	26295852	PR-2	Tc09_g031660	
Chr9	26452025	26452786	PR-5	Tc09_g031980	
Chr9	26838009	26841044	PR-7	Tc09_g032690	Chr9PR-7.5
Chr9	26843341	26846664	PR-7	Tc09_g032710	Chr9PR-7.5
Chr9	26853874	26856781	PR-7	Tc09_g032720	Chr9PR-7.5
Chr9	27978971	27981575	PR-2	Tc09_g034460	
Chr9	28169930	28171435	PR-9	Tc09_g034910	Chr9PR-9.6
Chr9	28177251	28179090	PR-9	Tc09_g034930	Chr9PR-9.6
Chr9	28180582	28182204	PR-9	Tc09_g034950	Chr9PR-9.6
Chr9	28271459	28271896	PR-14	Tc09_g035150	Chr9PR-14.
Chr9	28273057	28273520	PR-14	Tc09_g035160	Chr9PR-14.
Chr10	535646	537418	PR-1	Tc10_g000980	
Chr10	1257226	1258967	PR-2	Tc10_g002190	
Chr10	1321904	1325507	PR-7	Tc10_g002300	Chr10PR-7.1

Chr10	1326811	1330184	PR-7	Tc10_g002310	Chr10PR-7.1
Chr10	1331539	1343180	PR-7	Tc10_g002320	Chr10PR-7.1
Chr10	1359081	1365372	PR-7	Tc10_g002340	Chr10PR-7.1
Chr10	1374740	1384788	PR-7	Tc10_g002360	Chr10PR-7.1
Chr10	1680770	1681627	PR-5	Tc10_g002890	
Chr10	1889638	1891207	PR-2	Tc10_g003270	
Chr10	3651755	3652072	PR-6	Tc10_g005840	Chr10PR-6.2
Chr10	3660102	3660412	PR-6	Tc10_g005870	Chr10PR-6.2
Chr10	3662441	3662758	PR-6	Tc10_g005880	Chr10PR-6.2
Chr10	3669109	3669426	PR-6	Tc10_g005890	Chr10PR-6.2
Chr10	3678425	3684937	PR-6	Tc10_g005920	Chr10PR-6.2
Chr10	7845864	7846505	PR-16	Tc10_g009710	
Chr10	9700039	9702951	PR-16	Tc10_g011101	
Chr10	9724411	9725695	PR-4	Tc10_g011130	
Chr10	13313715	13314278	PR-10	Tc10_g014440	
Chr10	13971831	13973290	PR-8	Tc10_g015260	Chr10PR-8.3
Chr10	14003058	14003975	PR-8	Tc10_g015330	Chr10PR-8.3
Chr10	14505716	14507362	PR-9	Tc10_g015970	Chr10PR-9.4
Chr10	14567534	14568895	PR-9	Tc10_g016040	Chr10PR-9.4
Chr10	14583432	14584744	PR-9	Tc10_g016070	Chr10PR-9.4
Chr10	14585210	14586406	PR-9	Tc10_g016080	Chr10PR-9.4
Chr10	14753971	14754533	PR-14	Tc10_g016320	
Unmapped			PR-10	Tc00_g031750	
Unmapped			PR-10	Tc00_g071880	
Unmapped			PR-11	Tc00_g043900	
Unmapped			PR-16	Tc00_g013300	
Unmapped			PR-16	Tc00_g037180	
Unmapped			PR-16	Tc00_g054490	
Unmapped			PR-16	Tc00_g054500	
Unmapped			PR-16	Tc00_g076490	
Unmapped			PR-2	Tc00_g034720	
Unmapped			PR-2	Tc00_g054290	
Unmapped			PR-2	Tc00_g083950	
Unmapped			PR-4	Tc00_g012980	
Unmapped			PR-5	Tc00_g056050	
Unmapped			PR-5	Tc00_g056060	
Unmapped			PR-5	Tc00_g056070	
Unmapped			PR-5	Tc00_g056080	

Unmapped			PR-5	Tc00_g056110	
Unmapped			PR-5	Tc00_g060970	
Unmapped			PR-6	Tc00_g053480	
Unmapped			PR-7	Tc00_g001690	
Unmapped			PR-7	Tc00_g007300	
Unmapped			PR-7	Tc00_g013470	
Unmapped			PR-7	Tc00_g017310	
Unmapped			PR-7	Tc00_g032610	
Unmapped			PR-7	Tc00_g038560	
Unmapped			PR-8	Tc00_g024510	
Unmapped			PR-9	Tc00_g014230	
Unmapped			PR-9	Tc00_g020190	
Unmapped			PR-9	Tc00_g040640	
Unmapped			PR-9	Tc00_g044710	
Unmapped			PR-9	Tc00_g045360	
Unmapped			PR-9	Tc00_g045400	
Unmapped			PR-9	Tc00_g045440	
Unmapped			PR-9	Tc00_g045460	
Unmapped			PR-9	Tc00_g045610	
Unmapped			PR-9	Tc00_g045630	
Unmapped			PR-9	Tc00_g045940	
Unmapped			PR-9	Tc00_g054480	
