# Supplementary material for: Theobroma cacao L. pathogenesis-related gene tandem array members show diverse expression dynamics in response to pathogen colonization
Source: BMC Genomics. 2016 May 17;17:363. doi: 10.1186/s12864-016-2693-3 (PMC4869279; doi:10.1186/s12864-016-2693-3)
Supplement: Additional file 2: Figure S1. — Karyogram depicting the position of PR genes along the length of chromosomes based on the Matina genome sequence. Due to resolution of the image lines representing nearby genes partially overlap. (PDF 4169 kb) [file 12864_2016_2693_MOESM2_ESM.pdf]

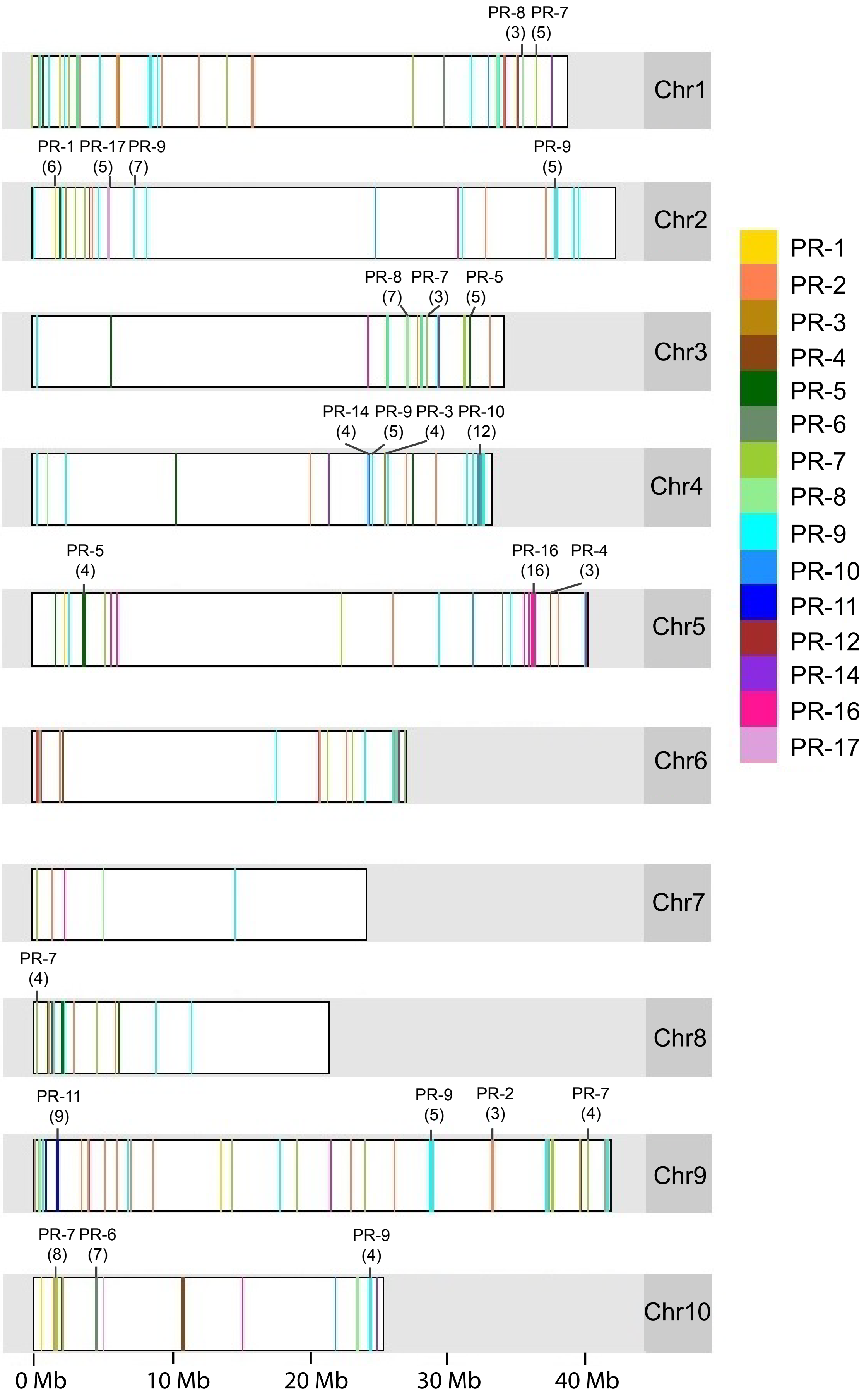

Figure S1. Karyogram depicting the position of PR genes along the length of chromosomes based on the Matina genome sequence. Tandem arrays are labelled above the chromosomes with gene family and number of genes in the array in parentheses. Length of chromosomes is shown in Mb. Due to resolution of the image, lines for physically clustered genes may overlap.
