# Supplementary material for: Theobroma cacao L. pathogenesis-related gene tandem array members show diverse expression dynamics in response to pathogen colonization
Source: BMC Genomics. 2016 May 17;17:363. doi: 10.1186/s12864-016-2693-3 (PMC4869279; doi:10.1186/s12864-016-2693-3)
Supplement: Additional file 3: Table S3. — Gene IDs and positions of Matina PR genes mapped to the ten cacao chromosomes. Those not mapped to the ten chromosomes are appended to the bottom of the list without positional information. (PDF 4169 kb) [file 12864_2016_2693_MOESM3_ESM.pdf]

Supplemental Table S3 - PR gene positions in the Matina 1-6 genome, color coded by PR gene family					
Chromosome	Start	Stop	Gene Family	Gene ID	Tandem Array ID
Chr1	38970	43636	PR-7	Thecc1EG000009	
Chr1	398604	400143	PR-3	Thecc1EG000095	Chr1PR-3.1
Chr1	407766	409127	PR-3	Thecc1EG000096	Chr1PR-3.1
Chr1	581637	583276	PR-9	Thecc1EG000145	
Chr1	747046	754136	PR-5	Thecc1EG000193	Chr1PR-5.2
Chr1	754379	756442	PR-5	Thecc1EG000197	Chr1PR-5.2
Chr1	1211500	1213275	PR-9	Thecc1EG000295	
Chr1	2026978	2027875	PR-1	Thecc1EG000491	
Chr1	2357330	2362741	PR-9	Thecc1EG000564	
Chr1	2748608	2752027	PR-7	Thecc1EG000640	Chr1PR-7.3
Chr1	2752856	2758066	PR-7	Thecc1EG000641	Chr1PR-7.3
Chr1	3307000	3310286	PR-7	Thecc1EG000749	
Chr1	3382352	3384526	PR-9	Thecc1EG000762	
Chr1	3470261	3472558	PR-2	Thecc1EG000782	
Chr1	4992850	4995297	PR-9	Thecc1EG001054	
Chr1	6194523	6196823	PR-2	Thecc1EG001274	Chr1PR-2.4
Chr1	6197493	6202783	PR-2	Thecc1EG001275	Chr1PR-2.4
Chr1	6253057	6261425	PR-3	Thecc1EG001280	
Chr1	8613801	8616925	PR-9	Thecc1EG001664	
Chr1	9131329	9134274	PR-9	Thecc1EG001737	
Chr1	9483225	9487563	PR-2	Thecc1EG001794	
Chr1	12130265	12134160	PR-2	Thecc1EG002168	
Chr1	14155420	14183715	PR-7	Thecc1EG002479	
Chr1	16047163	16060417	PR-2	Thecc1EG002746	
Chr1	27707568	27712092	PR-7	Thecc1EG003899	
Chr1	29913675	29914468	PR-6	Thecc1EG004163	
Chr1	31982011	31984949	PR-9	Thecc1EG004510	
Chr1	33192758	33193694	PR-10	Thecc1EG004731	
Chr1	33839678	33841234	PR-8	Thecc1EG004867	
Chr1	34041089	34042770	PR-9	Thecc1EG004905	
Chr1	34305919	34307096	PR-3	Thecc1EG004953	
Chr1	34442576	34443701	PR-16	Thecc1EG004977	
Chr1	35302482	35303571	PR-1	Thecc1EG005149	
Chr1	35365490	35366160	PR-14	Thecc1EG005159	
Chr1	35671159	35672532	PR-8	Thecc1EG005226	Chr1PR-8.5
Chr1	35674569	35675963	PR-8	Thecc1EG005227	Chr1PR-8.5
Chr1	35712363	35718588	PR-8	Thecc1EG005236	Chr1PR-8.5

Chr1	36672018	36676125	PR-7	Thecc1EG005449	Chr1PR-7.6
Chr1	36676173	36681516	PR-7	Thecc1EG005450	Chr1PR-7.6
Chr1	36681565	36693192	PR-7	Thecc1EG005451	Chr1PR-7.6
Chr1	36689354	36698417	PR-7	Thecc1EG005452	Chr1PR-7.6
Chr1	36697842	36703755	PR-7	Thecc1EG005453	Chr1PR-7.6
Chr1	37871369	37872238	PR-14	Thecc1EG005703	
Chr2	132261	139170	PR-9	Thecc1EG005974	
Chr2	1726070	1726919	PR-1	Thecc1EG006331	Chr2PR-1.1
Chr2	1727300	1728091	PR-1	Thecc1EG006332	Chr2PR-1.1
Chr2	1729729	1732901	PR-1	Thecc1EG006333	Chr2PR-1.1
Chr2	1736606	1737400	PR-1	Thecc1EG006335	Chr2PR-1.1
Chr2	2065004	2066485	PR-5	Thecc1EG006403	
Chr2	2117128	2118951	PR-9	Thecc1EG006416	
Chr2	2479366	2481072	PR-3	Thecc1EG006498	
Chr2	3126695	3129959	PR-7	Thecc1EG006646	
Chr2	3167231	3170972	PR-5	Thecc1EG006654	Chr2PR-5.2
Chr2	3176115	3179786	PR-5	Thecc1EG006655	Chr2PR-5.2
Chr2	3775808	3783306	PR-7	Thecc1EG006796	
Chr2	4126578	4127117	PR-12	Thecc1EG006852	
Chr2	4349987	4355168	PR-2	Thecc1EG006900	
Chr2	4794019	4796729	PR-9	Thecc1EG007001	
Chr2	5551606	5554475	PR-17	Thecc1EG007152	Chr2PR-17.3
Chr2	5554481	5555659	PR-17	Thecc1EG007153	Chr2PR-17.3
Chr2	5558506	5561482	PR-17	Thecc1EG007154	Chr2PR-17.3
Chr2	5566744	5567758	PR-17	Thecc1EG007156	Chr2PR-17.3
Chr2	5567811	5577818	PR-17	Thecc1EG007157	Chr2PR-17.3
Chr2	7394768	7402554	PR-9	Thecc1EG007510	Chr2PR-9.4
Chr2	7403090	7407224	PR-9	Thecc1EG007511	Chr2PR-9.4
Chr2	7407271	7413493	PR-9	Thecc1EG007512	Chr2PR-9.4
Chr2	7413305	7415134	PR-9	Thecc1EG007513	Chr2PR-9.4
Chr2	7417040	7418715	PR-9	Thecc1EG007514	Chr2PR-9.4
Chr2	7439241	7443374	PR-9	Thecc1EG007517	Chr2PR-9.4
Chr2	7447846	7449481	PR-9	Thecc1EG007520	Chr2PR-9.4
Chr2	8334315	8336188	PR-9	Thecc1EG007683	
Chr2	24981903	24982708	PR-10	Thecc1EG009531	Chr2PR-10.5
Chr2	25024282	25025861	PR-10	Thecc1EG009534	Chr2PR-10.5
Chr2	30987403	30988426	PR-16	Thecc1EG047006	
Chr2	31351157	31352561	PR-9	Thecc1EG010178	
Chr2	33006607	33010235	PR-2	Thecc1EG010376	
Chr2	37355734	37360729	PR-2	Thecc1EG011015	
Chr2	38066685	38068465	PR-9	Thecc1EG011110	Chr2PR-10.6
Chr2	38099456	38101161	PR-9	Thecc1EG011120	Chr2PR-10.6

Chr2	38104677	38114221	PR-9	Thecc1EG011122	Chr2PR-10.6
Chr2	38115416	38117955	PR-9	Thecc1EG011127	Chr2PR-10.6
Chr2	38125903	38132675	PR-9	Thecc1EG011131	Chr2PR-10.6
Chr2	39415824	39417517	PR-9	Thecc1EG011321	Chr2PR-10.7
Chr2	39424078	39426119	PR-9	Thecc1EG011322	Chr2PR-10.7
Chr2	39784485	39788147	PR-9	Thecc1EG011379	
Chr3	328975	330626	PR-9	Thecc1EG011986	
Chr3	5703693	5724899	PR-5	Thecc1EG012750	
Chr3	24480780	24482254	PR-16	Thecc1EG014789	
Chr3	25746880	25750071	PR-7	Thecc1EG015025	
Chr3	25948395	25950349	PR-9	Thecc1EG015053	
Chr3	27251393	27261927	PR-8	Thecc1EG015301	Chr3PR-8.1
Chr3	27261974	27270447	PR-8	Thecc1EG015302	Chr3PR-8.1
Chr3	27295158	27296498	PR-8	Thecc1EG015309	Chr3PR-8.1
Chr3	27296548	27300338	PR-8	Thecc1EG015310	Chr3PR-8.1
Chr3	27302986	27307234	PR-8	Thecc1EG015311	Chr3PR-8.1
Chr3	27307314	27308817	PR-8	Thecc1EG015312	Chr3PR-8.1
Chr3	27316543	27317507	PR-8	Thecc1EG015314	Chr3PR-8.1
Chr3	28004792	28007761	PR-2	Thecc1EG015448	
Chr3	28291446	28296197	PR-7	Thecc1EG047016	
Chr3	28361776	28363762	PR-9	Thecc1EG015526	
Chr3	28708331	28713572	PR-7	Thecc1EG015601	Chr3PR-7.2
Chr3	28714480	28717151	PR-7	Thecc1EG015602	Chr3PR-7.2
Chr3	28716513	28725233	PR-7	Thecc1EG015603	Chr3PR-7.2
Chr3	28757267	28760786	PR-2	Thecc1EG015614	
Chr3	29543107	29545374	PR-9	Thecc1EG015775	
Chr3	29603566	29604758	PR-14	Thecc1EG015788	
Chr3	31421523	31428659	PR-7	Thecc1EG016128	Chr3PR-7.3
Chr3	31428746	31431000	PR-7	Thecc1EG016129	Chr3PR-7.3
Chr3	31565837	31569764	PR-7	Thecc1EG016158	Chr3PR-7.4
Chr3	31570739	31575604	PR-7	Thecc1EG016159	Chr3PR-7.4
Chr3	31839269	31840236	PR-5	Thecc1EG016204	Chr3PR-5.5
Chr3	31845292	31846336	PR-5	Thecc1EG016206	Chr3PR-5.5
Chr3	31847013	31849966	PR-5	Thecc1EG016207	Chr3PR-5.5
Chr3	31851312	31852270	PR-5	Thecc1EG016208	Chr3PR-5.5
Chr3	31854759	31856092	PR-5	Thecc1EG016209	Chr3PR-5.5
Chr3	33366769	33369207	PR-2	Thecc1EG016546	
Chr4	324124	325508	PR-9	Thecc1EG016814	
Chr4	1082546	1084164	PR-8	Thecc1EG016958	
Chr4	2432036	2434089	PR-9	Thecc1EG017229	Chr4PR-9.1
Chr4	2435085	2444110	PR-9	Thecc1EG017230	Chr4PR-9.1

Chr4	10482455	10484484	PR-5	Thecc1EG018063	
Chr4	20312218	20317357	PR-2	Thecc1EG019120	
Chr4	21640200	21640902	PR-14	Thecc1EG019299	
Chr4	24438737	24442171	PR-9	Thecc1EG019706	
Chr4	24461787	24462534	PR-14	Thecc1EG019710	Chr4PR-14.2
Chr4	24480968	24481990	PR-14	Thecc1EG019712	Chr4PR-14.2
Chr4	24502230	24505993	PR-14	Thecc1EG019716	Chr4PR-14.2
Chr4	24518923	24519747	PR-14	Thecc1EG019718	Chr4PR-14.2
Chr4	24737036	24781021	PR-9	Thecc1EG019752	Chr4PR-9.3
Chr4	24737755	24740565	PR-9	Thecc1EG019753	Chr4PR-9.3
Chr4	24758134	24759674	PR-9	Thecc1EG019756	Chr4PR-9.3
Chr4	24785471	24788494	PR-9	Thecc1EG019760	Chr4PR-9.3
Chr4	24791739	24794455	PR-9	Thecc1EG019761	Chr4PR-9.3
Chr4	25647468	25651791	PR-3	Thecc1EG019909	Chr4PR-3.4
Chr4	25694796	25696329	PR-3	Thecc1EG019912	Chr4PR-3.4
Chr4	25703822	25704973	PR-3	Thecc1EG019913	Chr4PR-3.4
Chr4	25705022	25709845	PR-3	Thecc1EG019914	Chr4PR-3.4
Chr4	25896908	25899603	PR-9	Thecc1EG019959	
Chr4	27276438	27279822	PR-2	Thecc1EG020208	
Chr4	27670729	27672383	PR-5	Thecc1EG020283	
Chr4	29413526	29415586	PR-2	Thecc1EG020611	
Chr4	31669364	31671441	PR-9	Thecc1EG021109	Chr4PR-9.5
Chr4	31671546	31678459	PR-9	Thecc1EG021110	Chr4PR-9.5
Chr4	32119104	32121715	PR-9	Thecc1EG021202	
Chr4	32460298	32461096	PR-10	Thecc1EG021277	Chr4PR-10.6
Chr4	32461731	32462956	PR-10	Thecc1EG021278	Chr4PR-10.6
Chr4	32463079	32464245	PR-10	Thecc1EG021279	Chr4PR-10.6
Chr4	32469859	32470869	PR-10	Thecc1EG021281	Chr4PR-10.6
Chr4	32472553	32473339	PR-10	Thecc1EG021282	Chr4PR-10.6
Chr4	32477626	32478429	PR-10	Thecc1EG021284	Chr4PR-10.6
Chr4	32482792	32483593	PR-10	Thecc1EG021287	Chr4PR-10.6
Chr4	32485017	32485797	PR-10	Thecc1EG021288	Chr4PR-10.6
Chr4	32486846	32487676	PR-10	Thecc1EG021289	Chr4PR-10.6
Chr4	32489180	32490197	PR-10	Thecc1EG021291	Chr4PR-10.6
Chr4	32495448	32496548	PR-10	Thecc1EG021294	Chr4PR-10.6
Chr4	32496618	32497398	PR-10	Thecc1EG021295	Chr4PR-10.6
Chr4	32620132	32622079	PR-3	Thecc1EG021326	
Chr4	32685444	32691798	PR-2	Thecc1EG021342	
Chr4	32813328	32814949	PR-9	Thecc1EG021378	
Chr4	32911639	32915967	PR-9	Thecc1EG021402	
Chr5	1670681	1671852	PR-5	Thecc1EG021897	

Chr5	2364984	2366039	PR-1	Thecc1EG022043	
Chr5	2695588	2698762	PR-9	Thecc1EG022117	
Chr5	3755038	3756269	PR-5	Thecc1EG022309	Chr5PR-5.1
Chr5	3758335	3760031	PR-5	Thecc1EG022310	Chr5PR-5.1
Chr5	3791716	3792976	PR-5	Thecc1EG022314	Chr5PR-5.1
Chr5	3840968	3842301	PR-5	Thecc1EG022319	Chr5PR-5.1
Chr5	5340366	5344406	PR-7	Thecc1EG022517	
Chr5	5797021	5798314	PR-16	Thecc1EG022573	
Chr5	6189727	6194196	PR-16	Thecc1EG022636	
Chr5	22512938	22520820	PR-7	Thecc1EG024130	
Chr5	26226678	26234169	PR-2	Thecc1EG024509	Chr5PR-2.2
Chr5	26267724	26269539	PR-2	Thecc1EG024514	Chr5PR-2.2
Chr5	29642537	29644495	PR-9	Thecc1EG024936	
Chr5	32085632	32088661	PR-10	Thecc1EG025354	Chr5PR-10.3
Chr5	32103102	32104002	PR-10	Thecc1EG025358	Chr5PR-10.3
Chr5	34273894	34274779	PR-6	Thecc1EG025715	Chr5PR-6.4
Chr5	34279871	34281266	PR-6	Thecc1EG025717	Chr5PR-6.4
Chr5	34805481	34844123	PR-9	Thecc1EG025806	
Chr5	35819221	35823224	PR-16	Thecc1EG025978	
Chr5	36102473	36104098	PR-16	Thecc1EG026019	
Chr5	36336863	36348253	PR-16	Thecc1EG026063	Chr5PR-16.5
Chr5	36352033	36353232	PR-16	Thecc1EG026065	Chr5PR-16.5
Chr5	36359172	36362528	PR-16	Thecc1EG026068	Chr5PR-16.5
Chr5	36362595	36365298	PR-16	Thecc1EG026069	Chr5PR-16.5
Chr5	36380524	36386728	PR-16	Thecc1EG026071	Chr5PR-16.5
Chr5	36386562	36388564	PR-16	Thecc1EG026073	Chr5PR-16.5
Chr5	36397149	36398236	PR-16	Thecc1EG026075	Chr5PR-16.5
Chr5	36398233	36425560	PR-16	Thecc1EG026076	Chr5PR-16.5
Chr5	36436073	36443005	PR-16	Thecc1EG026082	Chr5PR-16.5
Chr5	36443053	36450882	PR-16	Thecc1EG026084	Chr5PR-16.5
Chr5	36454203	36478357	PR-16	Thecc1EG026086	Chr5PR-16.5
Chr5	36474279	36524416	PR-16	Thecc1EG026091	Chr5PR-16.5
Chr5	36499184	36500352	PR-16	Thecc1EG026092	Chr5PR-16.5
Chr5	36519844	36520792	PR-16	Thecc1EG026096	Chr5PR-16.5
Chr5	36523344	36524570	PR-16	Thecc1EG026097	Chr5PR-16.5
Chr5	36543803	36544957	PR-16	Thecc1EG026105	Chr5PR-16.5
Chr5	37696481	37697365	PR-4	Thecc1EG026324	Chr5PR-4.6
Chr5	37701027	37709138	PR-4	Thecc1EG026326	Chr5PR-4.6
Chr5	37714713	37716374	PR-4	Thecc1EG026330	Chr5PR-4.6
Chr5	37736830	37738060	PR-16	Thecc1EG026333	
Chr5	37761863	37762800	PR-4	Thecc1EG026340	
Chr5	38325876	38329574	PR-2	Thecc1EG026450	
Chr5	40176700	40178520	PR-9	Thecc1EG026820	

Chr5	40355024	40356650	PR-16	Thecc1EG026861	
Chr6	370465	371631	PR-16	Thecc1EG026931	Chr6PR-16.1
Chr6	372939	378215	PR-16	Thecc1EG026932	Chr6PR-16.1
Chr6	425768	428602	PR-3	Thecc1EG026942	
Chr6	565963	575546	PR-7	Thecc1EG026974	
Chr6	687608	688111	PR-14	Thecc1EG026996	Chr6PR-14.2
Chr6	688555	689089	PR-14	Thecc1EG026997	Chr6PR-14.2
Chr6	2009028	2026798	PR-2	Thecc1EG027171	
Chr6	2244627	2245484	PR-4	Thecc1EG027204	
Chr6	17845152	17848170	PR-9	Thecc1EG028741	
Chr6	20842639	20844719	PR-16	Thecc1EG029131	Chr6PR-16.3
Chr6	20862180	20863075	PR-16	Thecc1EG029135	Chr6PR-16.3
Chr6	20993519	20997653	PR-7	Thecc1EG029155	
Chr6	21455725	21458909	PR-7	Thecc1EG029233	
Chr6	22820859	22823230	PR-2	Thecc1EG029478	
Chr6	23349015	23355024	PR-7	Thecc1EG029591	
Chr6	24237351	24242736	PR-9	Thecc1EG029767	
Chr6	26264259	26266610	PR-9	Thecc1EG030222	
Chr6	26374015	26377587	PR-2	Thecc1EG030245	
Chr6	26496525	26502924	PR-9	Thecc1EG030283	
Chr6	26573080	26576918	PR-7	Thecc1EG030299	
Chr6	26689344	26690019	PR-14	Thecc1EG030328	
Chr6	27171220	27177050	PR-7	Thecc1EG030468	
Chr7	294277	301433	PR-7	Thecc1EG030557	
Chr7	1460699	1467312	PR-2	Thecc1EG030805	
Chr7	2417342	2418208	PR-16	Thecc1EG030994	
Chr7	5183291	5185283	PR-8	Thecc1EG031497	
Chr7	14781355	14784469	PR-9	Thecc1EG032808	
Chr8	246545	249638	PR-7	Thecc1EG033805	Chr8PR-7.1
Chr8	250441	269283	PR-7	Thecc1EG033806	Chr8PR-7.1
Chr8	268993	272502	PR-7	Thecc1EG033809	Chr8PR-7.1
Chr8	274222	277466	PR-7	Thecc1EG033810	Chr8PR-7.1
Chr8	962742	964086	PR-5	Thecc1EG033984	
Chr8	1137539	1140373	PR-2	Thecc1EG034024	
Chr8	1353624	1354652	PR-12	Thecc1EG034086	
Chr8	1493708	1495530	PR-9	Thecc1EG034114	
Chr8	2075283	2077743	PR-5	Thecc1EG034243	Chr8PR-5.2
Chr8	2087537	2090040	PR-5	Thecc1EG034245	Chr8PR-5.2
Chr8	2224839	2229219	PR-7	Thecc1EG034269	
Chr8	2260133	2262131	PR-9	Thecc1EG034278	

Chr8	2897102	2899238	PR-2	Thecc1EG034422	
Chr8	4575941	4579741	PR-7	Thecc1EG034736	
Chr8	5988115	5995353	PR-2	Thecc1EG035012	
Chr8	6226812	6229286	PR-5	Thecc1EG035053	
Chr8	8855786	8857516	PR-9	Thecc1EG035423	
Chr8	11449195	11452325	PR-9	Thecc1EG035691	
Chr9	134564	137947	PR-2	Thecc1EG036758	
Chr9	367926	369673	PR-9	Thecc1EG036816	
Chr9	410586	411381	PR-1	Thecc1EG036825	
Chr9	645825	649597	PR-9	Thecc1EG036873	
Chr9	938748	944094	PR-11	Thecc1EG036927	
Chr9	1738958	1741618	PR-11	Thecc1EG037102	Chr9PR-11.1
Chr9	1742221	1746075	PR-11	Thecc1EG037103	Chr9PR-11.1
Chr9	1747571	1752188	PR-11	Thecc1EG037104	Chr9PR-11.1
Chr9	1752241	1753840	PR-11	Thecc1EG037105	Chr9PR-11.1
Chr9	1754711	1759539	PR-11	Thecc1EG037107	Chr9PR-11.1
Chr9	1759531	1764203	PR-11	Thecc1EG037108	Chr9PR-11.1
Chr9	1764238	1769028	PR-11	Thecc1EG037109	Chr9PR-11.1
Chr9	1777647	1779310	PR-11	Thecc1EG037111	Chr9PR-11.1
Chr9	1779359	1781333	PR-11	Thecc1EG037112	Chr9PR-11.1
Chr9	3436551	3439194	PR-2	Thecc1EG037436	
Chr9	3902544	3906668	PR-7	Thecc1EG037518	
Chr9	4052575	4055968	PR-16	Thecc1EG037546	
Chr9	5158780	5163277	PR-2	Thecc1EG037759	
Chr9	6109289	6112057	PR-2	Thecc1EG037934	
Chr9	6851364	6854612	PR-9	Thecc1EG038079	
Chr9	7063382	7068716	PR-2	Thecc1EG038109	
Chr9	8686397	8690185	PR-2	Thecc1EG038357	
Chr9	13637086	13637886	PR-1	Thecc1EG038872	Chr9PR-1.2
Chr9	13639036	13639762	PR-1	Thecc1EG038873	Chr9PR-1.2
Chr9	14374415	14377545	PR-7	Thecc1EG038960	
Chr9	17902573	17905378	PR-9	Thecc1EG039289	
Chr9	19150129	19161776	PR-7	Thecc1EG039425	
Chr9	21602646	21603582	PR-16	Thecc1EG039645	Chr9PR-16.3
Chr9	21604434	21605321	PR-16	Thecc1EG039646	Chr9PR-16.3
Chr9	23054779	23063810	PR-2	Thecc1EG039796	
Chr9	24055817	24067197	PR-7	Thecc1EG039861	
Chr9	26281573	26290591	PR-2	Thecc1EG040040	
Chr9	28867512	28878972	PR-9	Thecc1EG040312	Chr9PR-9.4
Chr9	28900323	28913650	PR-9	Thecc1EG040315	Chr9PR-9.4
Chr9	28942562	28944397	PR-9	Thecc1EG040320	Chr9PR-9.4
Chr9	28981897	28983607	PR-9	Thecc1EG040327	Chr9PR-9.4

Chr9	29003771	29005794	PR-9	Thecc1EG040331	Chr9PR-9.4
Chr9	33307006	33308832	PR-2	Thecc1EG040768	
Chr9	33390249	33392278	PR-2	Thecc1EG040785	Chr9PR-2.5
Chr9	33393078	33395318	PR-2	Thecc1EG040786	Chr9PR-2.5
Chr9	33416912	33419009	PR-2	Thecc1EG040791	Chr9PR-2.5
Chr9	37324293	37326613	PR-16	Thecc1EG041376	Chr9PR-16.6
Chr9	37328816	37330133	PR-16	Thecc1EG041377	Chr9PR-16.6
Chr9	37330732	37332369	PR-9	Thecc1EG041378	
Chr9	37442073	37446700	PR-2	Thecc1EG041403	
Chr9	37711045	37715553	PR-7	Thecc1EG041449	
Chr9	37801525	37807811	PR-7	Thecc1EG041462	Chr9PR-7.7
Chr9	37811034	37827825	PR-7	Thecc1EG041463	Chr9PR-7.7
Chr9	39742134	39745187	PR-2	Thecc1EG041833	
Chr9	39900122	39901490	PR-5	Thecc1EG041871	
Chr9	40283527	40292405	PR-7	Thecc1EG041950	Chr9PR-7.8
Chr9	40287674	40292567	PR-7	Thecc1EG041951	Chr9PR-7.8
Chr9	40292614	40309871	PR-7	Thecc1EG041952	Chr9PR-7.8
Chr9	40299619	40329996	PR-7	Thecc1EG041957	Chr9PR-7.8
Chr9	41528294	41531544	PR-2	Thecc1EG042208	
Chr9	41718285	41727412	PR-9	Thecc1EG042259	Chr9PR-9.9
Chr9	41732924	41738678	PR-9	Thecc1EG042261	Chr9PR-9.9
Chr9	41826527	41827275	PR-14	Thecc1EG042284	Chr9PR-14.10
Chr9	41828203	41828948	PR-14	Thecc1EG042285	Chr9PR-14.10
Chr10	568906	571643	PR-1	Thecc1EG042446	
Chr10	1425026	1428017	PR-2	Thecc1EG042611	
Chr10	1611248	1619758	PR-7	Thecc1EG042652	Chr10PR-7.1
Chr10	1620365	1624378	PR-7	Thecc1EG042653	Chr10PR-7.1
Chr10	1625325	1628768	PR-7	Thecc1EG046952	Chr10PR-7.1
Chr10	1629514	1632741	PR-7	Thecc1EG046953	Chr10PR-7.1
Chr10	1644748	1652068	PR-7	Thecc1EG042656	Chr10PR-7.1
Chr10	1645533	1660966	PR-7	Thecc1EG042657	Chr10PR-7.1
Chr10	1660065	1667653	PR-7	Thecc1EG042659	Chr10PR-7.1
Chr10	1663592	1667693	PR-7	Thecc1EG042660	Chr10PR-7.1
Chr10	1982315	1983905	PR-5	Thecc1EG042726	
Chr10	2192233	2194119	PR-2	Thecc1EG042768	
Chr10	4550730	4551474	PR-6	Thecc1EG043194	Chr10PR-6.2
Chr10	4559293	4559803	PR-6	Thecc1EG043196	Chr10PR-6.2
Chr10	4561388	4562015	PR-6	Thecc1EG046959	Chr10PR-6.2
Chr10	4565525	4580115	PR-6	Thecc1EG043198	Chr10PR-6.2
Chr10	4565536	4566082	PR-6	Thecc1EG046960	Chr10PR-6.2
Chr10	4576505	4577038	PR-6	Thecc1EG043201	Chr10PR-6.2

Chr10	4582260	4582744	PR-6	Thecc1EG043202	Chr10PR-6.2
Chr10	5101255	5110883	PR-17	Thecc1EG043303	
Chr10	10866300	10867951	PR-4	Thecc1EG043895	
Chr10	15211018	15211919	PR-16	Thecc1EG044263	
Chr10	21995785	21996731	PR-10	Thecc1EG045046	
Chr10	23553698	23580519	PR-8	Thecc1EG045276	Chr10PR-8.3
Chr10	23602812	23603970	PR-8	Thecc1EG045283	Chr10PR-8.3
Chr10	24429996	24431594	PR-9	Thecc1EG045424	Chr10PR-9.4
Chr10	24431808	24447232	PR-9	Thecc1EG045425	Chr10PR-9.4
Chr10	24447255	24449409	PR-9	Thecc1EG045429	Chr10PR-9.4
Chr10	24507368	24509439	PR-9	Thecc1EG045437	Chr10PR-9.4
Chr10	25002097	25003389	PR-14	Thecc1EG045525	
Unmapped			PR-10	Thecc1EG046315	
Unmapped			PR-10	Thecc1EG046316	
Unmapped			PR-16	Thecc1EG045749	
Unmapped			PR-5	Thecc1EG046303	
