# Supplementary material for: Theobroma cacao L. pathogenesis-related gene tandem array members show diverse expression dynamics in response to pathogen colonization
Source: BMC Genomics. 2016 May 17;17:363. doi: 10.1186/s12864-016-2693-3 (PMC4869279; doi:10.1186/s12864-016-2693-3)
Supplement: Additional file 4: Table S4. — Gene IDs and BLASTp E-values for Arabidopsis thaliana PR loci. (PDF 4169 kb) [file 12864_2016_2693_MOESM4_ESM.pdf]

Supplemental Table S4 - Gene IDs and BLASTp E-value for Arabidopsis PR genes		
PR Gene Family	Gene ID	E-value
PR-1	AT2G14580	1.00E-62
PR-1	AT3G19690	5.00E-61
PR-1	AT4G33720	3.00E-60
PR-1	AT2G14610	3.00E-58
PR-1	AT1G50060	1.00E-55
PR-1	AT5G26130	9.00E-52
PR-1	AT4G33710	9.00E-51
PR-1	AT4G33730	3.00E-50
PR-1	AT4G25790	7.00E-50
PR-1	AT2G19990	4.00E-48
PR-1	AT5G57625	3.00E-47
PR-1	AT1G01310	1.00E-44
PR-1	AT1G50050	1.00E-43
PR-1	AT4G30320	6.00E-43
PR-1	AT4G25780	2.00E-42
PR-1	AT3G09590	2.00E-42
PR-1	AT4G31470	3.00E-40
PR-1	AT5G02730	2.00E-39
PR-1	AT4G07820	4.00E-37
PR-1	AT5G66590	1.00E-29
PR-1	AT2G19970	2.00E-25
PR-1	AT2G19980	2.00E-20
PR-2	AT4G16260	5.00E-116
PR-2	AT3G57260	3.00E-103
PR-2	AT3G57270	6.00E-99
PR-2	AT3G57240	7.00E-97
PR-2	AT5G56590	7.00E-74
PR-2	AT2G26600	8.00E-70
PR-2	AT5G42720	5.00E-69
PR-2	AT4G29360	1.00E-68
PR-2	AT5G20330	5.00E-67
PR-2	AT5G20390	7.00E-67
PR-2	AT1G32860	4.00E-65
PR-2	AT5G20340	4.00E-65
PR-2	AT1G33220	9.00E-65
PR-2	AT3G15800	2.00E-63
PR-2	AT2G05790	3.00E-63
PR-2	AT2G01630	1.00E-62
PR-2	AT2G16230	2.00E-62
PR-2	AT4G26830	3.00E-62
PR-2	AT2G27500	6.00E-62
PR-2	AT5G24318	1.00E-61
PR-2	AT5G55180	2.00E-61
PR-2	AT4G18340	4.00E-61
PR-2	AT1G66250	2.00E-60
PR-2	AT1G30080	1.00E-59
PR-2	AT5G42100	1.00E-59
PR-2	AT4G34480	3.00E-58
PR-2	AT1G77790	5.00E-58
PR-2	AT5G20560	4.00E-57
PR-2	AT1G11820	3.00E-55
PR-2	AT1G77780	2.00E-54
PR-2	AT3G07320	2.00E-54
PR-2	AT4G14080	3.00E-53
PR-2	AT5G58090	2.00E-52
PR-2	AT3G13560	4.00E-52
PR-2	AT3G61810	3.00E-51
PR-2	AT5G18220	4.00E-50
PR-2	AT3G46570	5.00E-50
PR-2	AT3G23770	1.00E-49
PR-2	AT4G31140	9.00E-49
PR-2	AT3G24330	9.00E-49
PR-2	AT3G04010	4.00E-48
PR-2	AT5G58480	1.00E-46
PR-2	AT3G55430	1.00E-44
PR-2	AT1G64760	2.00E-44
PR-2	AT2G19440	2.00E-43
PR-2	AT5G64790	3.00E-43
PR-2	AT2G39640	3.00E-42

PR-2	AT4G17180	3.00E-42
PR-2	AT5G20870	8.00E-39
PR-2	AT3G55780	2.00E-34
PR-3	AT3G12500	3.00E-86
PR-3	AT1G02360	2.00E-64
PR-3	AT4G01700	1.00E-61
PR-3	AT2G43570	2.00E-33
PR-3	AT1G05850	3.00E-32
PR-3	AT2G43590	2.00E-31
PR-3	AT3G54420	1.00E-30
PR-3	AT3G16920	2.00E-30
PR-3	AT2G43610	2.00E-29
PR-3	AT2G43620	1.00E-27
PR-3	AT2G43580	2.00E-27
PR-3	AT1G56680	2.00E-22
PR-3	AT2G43600	4.00E-22
PR-3	AT3G47540	1.00E-19
PR-4	AT3G04720	1.00E-60
PR-5	AT1G75030	7.00E-87
PR-5	AT1G18250	1.00E-86
PR-5	AT1G73620	3.00E-86
PR-5	AT1G75050	6.00E-85
PR-5	AT1G19320	2.00E-83
PR-5	AT4G38660	3.00E-83
PR-5	AT1G75040	5.00E-81
PR-5	AT1G77700	3.00E-78
PR-5	AT1G20030	6.00E-78
PR-5	AT4G36010	3.00E-77
PR-5	AT4G24180	5.00E-77
PR-5	AT1G75800	5.00E-77
PR-5	AT2G17860	5.00E-75
PR-5	AT5G24620	4.00E-71
PR-5	AT5G02140	1.00E-65
PR-5	AT4G38670	9.00E-65
PR-5	AT5G40020	2.00E-63
PR-5	AT5G38280	6.00E-59
PR-5	AT2G28790	1.00E-54
PR-5	AT4G36000	1.00E-51
PR-5	AT4G11650	6.00E-51
PR-5	AT4G18250	1.00E-43
PR-5	AT1G70250	6.00E-42

PR-5	AT2G24810	6.00E-27
PR-6	AT2G38870	5.00E-17
PR-6	AT2G38900	2.00E-14
PR-6	AT5G43580	8.00E-14
PR-6	AT5G43570	6.00E-07
PR-6	AT3G46860	1.00E-06
PR-7	AT5G67360	7.00E-160
PR-7	AT2G05920	3.00E-158
PR-7	AT1G04110	6.00E-158
PR-7	AT4G34980	1.00E-156
PR-7	AT3G14067	2.00E-154
PR-7	AT1G01900	8.00E-149
PR-7	AT3G14240	5.00E-148
PR-7	AT5G51750	1.00E-142
PR-7	AT2G04160	8.00E-140
PR-7	AT5G59810	1.00E-130
PR-7	AT4G00230	3.00E-129
PR-7	AT1G20160	2.00E-124
PR-7	AT5G59120	4.00E-123
PR-7	AT5G45650	8.00E-119
PR-7	AT5G59100	3.00E-116
PR-7	AT5G67090	6.00E-116
PR-7	AT5G59090	2.00E-115
PR-7	AT5G03620	4.00E-114
PR-7	AT5G59190	4.00E-113
PR-7	AT3G46850	3.00E-110
PR-7	AT3G46840	1.00E-108
PR-7	AT4G10520	1.00E-106
PR-7	AT4G10550	2.00E-105
PR-7	AT1G20150	5.00E-105
PR-7	AT4G10540	2.00E-104
PR-7	AT1G32950	5.00E-103
PR-7	AT1G66210	5.00E-103
PR-7	AT5G58830	1.00E-102
PR-7	AT5G59130	2.00E-102
PR-7	AT4G10510	6.00E-102
PR-7	AT1G32960	5.00E-100
PR-7	AT1G32940	8.00E-98
PR-7	AT1G66220	1.00E-97
PR-7	AT5G58820	2.00E-97
PR-7	AT4G26330	2.00E-95

PR-7	AT4G21650	4.00E-95
PR-7	AT4G21630	6.00E-95
PR-7	AT5G45640	1.00E-92
PR-7	AT4G10530	2.00E-92
PR-7	AT5G11940	2.00E-91
PR-7	AT4G21323	1.00E-89
PR-7	AT4G15040	3.00E-89
PR-7	AT4G21326	1.00E-83
PR-7	AT1G32970	5.00E-79
PR-7	AT4G30020	1.00E-77
PR-7	AT2G19170	3.00E-77
PR-7	AT4G20430	7.00E-71
PR-7	AT5G44530	7.00E-71
PR-7	AT1G30600	5.00E-70
PR-7	AT1G62340	2.00E-63
PR-8	AT5G24090	3.00E-100
PR-9	AT5G06720	8.00E-147
PR-9	AT5G06730	4.00E-145
PR-9	AT5G19880	2.00E-123
PR-9	AT3G32980	8.00E-106
PR-9	AT2G38380	8.00E-105
PR-9	AT3G49120	2.00E-104
PR-9	AT3G49110	2.00E-104
PR-9	AT2G18150	7.00E-104
PR-9	AT2G18140	2.00E-103
PR-9	AT5G19890	5.00E-103
PR-9	AT4G08770	6.00E-103
PR-9	AT4G08780	1.00E-101
PR-9	AT3G50990	1.00E-101
PR-9	AT4G36430	5.00E-101
PR-9	AT2G38390	9.00E-101
PR-9	AT5G66390	8.00E-98
PR-9	AT5G05340	2.00E-93
PR-9	AT1G44970	2.00E-88
PR-9	AT1G14550	4.00E-87
PR-9	AT1G49570	1.00E-86
PR-9	AT5G58400	2.00E-85
PR-9	AT1G14540	1.00E-84
PR-9	AT5G58390	2.00E-83
PR-9	AT4G16270	1.00E-82
PR-9	AT4G11290	3.00E-81

PR-9	AT2G35380	2.00E-80
PR-9	AT2G41480	5.00E-80
PR-9	AT1G68850	3.00E-79
PR-9	AT5G64120	3.00E-78
PR-9	AT3G01190	8.00E-78
PR-9	AT3G03670	1.00E-77
PR-9	AT5G39580	1.00E-77
PR-9	AT2G22420	5.00E-77
PR-9	AT5G17820	5.00E-77
PR-9	AT1G05260	1.00E-76
PR-9	AT5G15180	1.00E-76
PR-9	AT5G42180	2.00E-75
PR-9	AT3G21770	5.00E-75
PR-9	AT5G51890	1.00E-73
PR-9	AT4G33420	4.00E-73
PR-9	AT5G14130	2.00E-72
PR-9	AT2G18980	3.00E-70
PR-9	AT4G25980	5.00E-70
PR-9	AT1G30870	7.00E-70
PR-9	AT1G05250	3.00E-69
PR-9	AT1G05240	3.00E-69
PR-9	AT4G30170	2.00E-68
PR-9	AT1G71695	9.00E-68
PR-9	AT4G37530	2.00E-67
PR-9	AT5G67400	2.00E-67
PR-9	AT3G49960	3.00E-67
PR-9	AT5G24070	3.00E-67
PR-9	AT2G43480	6.00E-67
PR-9	AT1G77100	6.00E-66
PR-9	AT5G64110	6.00E-65
PR-9	AT2G34060	1.00E-64
PR-9	AT5G64100	3.00E-64
PR-9	AT2G39040	4.00E-64
PR-9	AT4G37520	4.00E-64
PR-9	AT4G17690	2.00E-63
PR-9	AT5G40150	4.00E-63
PR-9	AT2G24800	1.00E-61
PR-9	AT3G28200	1.00E-61
PR-9	AT4G33870	4.00E-61
PR-9	AT5G47000	2.00E-59
PR-9	AT4G26010	6.00E-59

PR-9	AT4G31760	3.00E-56
PR-9	AT1G24110	3.00E-56
PR-9	AT2G37130	5.00E-54
PR-9	AT1G34510	5.00E-53
PR-9	AT4G21960	4.00E-52
PR-9	AT5G22410	8.00E-52
PR-9	AT3G17070	3.00E-51
PR-9	AT3G42570	2.00E-14
PR-10	AT1G24020	2.00E-07
PR-11	AT4G19810	7.00E-154
PR-11	AT4G19800	5.00E-120
PR-11	AT4G19820	1.00E-118
PR-11	AT4G19760	1.00E-108
PR-11	AT4G19720	9.00E-107
PR-11	AT4G19750	2.00E-105
PR-11	AT4G19730	2.00E-85
PR-11	AT4G19770	3.00E-76
PR-11	AT4G19740	6.00E-45
PR-12	AT2G26020	3.00E-34
PR-12	AT5G44420	7.00E-34
PR-12	AT2G26010	2.00E-33
PR-12	AT5G44430	3.00E-33
PR-12	AT1G75830	1.00E-32
PR-12	AT1G19610	2.00E-15
PR-12	AT1G55010	5.00E-13
PR-13	AT1G72260	2.00E-85
PR-13	AT1G66100	4.00E-35
PR-13	AT5G36910	5.00E-35
PR-13	AT2G15010	9.00E-22
PR-14	AT5G59310	5.00E-24
PR-14	AT5G59320	3.00E-19
PR-14	AT2G38540	3.00E-18
PR-14	AT2G18370	2.00E-16
PR-14	AT3G51590	4.00E-15
PR-14	AT3G08770	6.00E-15
PR-14	AT5G01870	8.00E-15
PR-14	AT3G51600	3.00E-14
PR-14	AT2G38530	4.00E-14
PR-14	AT2G15050	4.00E-13
PR-14	AT4G33355	8.00E-12
PR-14	AT5G59330	8.00E-08
PR-14	AT2G15325	1.00E-06
PR-16	AT1G18980	5.00E-69
PR-16	AT1G18970	2.00E-68
PR-16	AT3G05950	7.00E-61
PR-16	AT3G04200	1.00E-60
PR-16	AT4G14630	7.00E-59
PR-16	AT5G39160	1.00E-57
PR-16	AT5G39130	2.00E-57
PR-16	AT5G39150	2.00E-57
PR-16	AT5G39190	2.00E-57
PR-16	AT5G39120	3.00E-57
PR-16	AT5G39110	4.00E-57
PR-16	AT5G39180	1.00E-56
PR-16	AT3G62020	4.00E-56
PR-16	AT5G38960	4.00E-56
PR-16	AT1G02335	2.00E-54
PR-16	AT1G09560	1.00E-53
PR-16	AT3G04190	3.00E-52
PR-16	AT5G38940	6.00E-52
PR-16	AT3G04170	8.00E-52
PR-16	AT3G05930	4.00E-51
PR-16	AT5G38930	7.00E-51
PR-16	AT3G04180	8.00E-50
PR-16	AT3G04150	6.00E-49
PR-16	AT5G26700	6.00E-46
PR-16	AT5G38910	1.00E-45
PR-16	AT1G74820	1.00E-43
PR-16	AT3G10080	4.00E-42
PR-16	AT1G10460	1.00E-39
PR-16	AT5G39100	1.00E-36
PR-16	AT1G72610	3.00E-36
PR-16	AT5G20630	2.00E-33
PR-16	AT5G61750	3.00E-30
PR-16	AT5G38950	2.00E-13
PR-17	AT2G15220	2.00E-101
PR-17	AT2G15130	9.00E-89
PR-17	AT2G15170	7.00E-12
