# Supplementary material for: Theobroma cacao L. pathogenesis-related gene tandem array members show diverse expression dynamics in response to pathogen colonization
Source: BMC Genomics. 2016 May 17;17:363. doi: 10.1186/s12864-016-2693-3 (PMC4869279; doi:10.1186/s12864-016-2693-3)
Supplement: Additional file 5: Table S5. — Gene IDs and BLASTp E-values for Brachypodium distachyon PR loci. (PDF 4169 kb) [file 12864_2016_2693_MOESM5_ESM.pdf]

Supplemental Table S5 - Gene IDs and BLASTp E-value for *Brachypodium distachyon* PR genes		
**PR Gene Family**	**Gene ID**	**E-value**
PR-1	Bradi1g57580	6.00E-43
PR-1	Bradi1g57540	1.00E-39
PR-1	Bradi3g53630	2.00E-36
PR-1	Bradi1g12360	1.00E-35
PR-1	Bradi4g00865	5.00E-34
PR-1	Bradi1g57590	9.00E-34
PR-1	Bradi2g14240	7.00E-32
PR-1	Bradi3g53637	2.00E-27
PR-1	Bradi4g38910	4.00E-25
PR-1	Bradi3g60230	2.00E-23
PR-1	Bradi3g60260	4.00E-23
PR-1	Bradi1g09637	4.00E-22
PR-1	Bradi3g53681	7.00E-20
PR-1	Bradi1g57575	4.00E-19
PR-1	Bradi2g14255	3.00E-09
PR-2	Bradi2g43056	3.00E-77
PR-2	Bradi2g60536	1.00E-74
PR-2	Bradi2g60490	4.00E-73
PR-2	Bradi2g60441	1.00E-71
PR-2	Bradi2g27140	2.00E-69
PR-2	Bradi2g22222	1.00E-66
PR-2	Bradi2g60497	1.00E-66
PR-2	Bradi1g15295	1.00E-66
PR-2	Bradi2g60542	2.00E-66
PR-2	Bradi2g52566	2.00E-65
PR-2	Bradi2g60557	7.00E-65
PR-2	Bradi2g22226	1.00E-64
PR-2	Bradi2g21142	1.00E-63
PR-2	Bradi2g49330	2.00E-61
PR-2	Bradi2g22224	5.00E-60
PR-2	Bradi1g38755	6.00E-55
PR-2	Bradi1g68450	6.00E-55
PR-2	Bradi5g09327	1.00E-54
PR-2	Bradi2g60534	3.00E-54
PR-2	Bradi3g44910	5.00E-54
PR-2	Bradi1g69610	9.00E-54
PR-2	Bradi4g36190	1.00E-53
PR-2	Bradi3g20770	4.00E-53
PR-2	Bradi1g12810	6.00E-52
PR-2	Bradi3g57610	8.00E-52
PR-2	Bradi2g60541	5.00E-51
PR-2	Bradi2g55690	6.00E-51
PR-2	Bradi3g07385	1.00E-50
PR-2	Bradi1g50080	2.00E-50
PR-2	Bradi2g23940	7.00E-50
PR-2	Bradi1g69020	9.00E-49
PR-2	Bradi1g25530	2.00E-48
PR-2	Bradi3g18220	3.00E-47
PR-2	Bradi1g10347	8.00E-47
PR-2	Bradi2g22228	1.00E-46
PR-2	Bradi1g36460	2.00E-46
PR-2	Bradi1g23640	2.00E-44
PR-2	Bradi1g53590	3.00E-44
PR-2	Bradi1g13232	7.00E-44
PR-2	Bradi1g56270	1.00E-42
PR-2	Bradi1g06050	1.00E-42
PR-2	Bradi4g09230	3.00E-42
PR-2	Bradi5g12137	5.00E-42
PR-2	Bradi4g34390	9.00E-42
PR-2	Bradi3g03520	1.00E-41
PR-2	Bradi1g25517	2.00E-41
PR-2	Bradi2g18420	4.00E-41
PR-2	Bradi4g15460	4.00E-41
PR-2	Bradi1g37160	2.00E-40
PR-2	Bradi3g40907	8.00E-39
PR-2	Bradi1g60410	2.00E-38
PR-2	Bradi2g18700	2.00E-37
PR-2	Bradi1g65197	2.00E-37

PR-2	Bradi1g26510	2.00E-34
PR-2	Bradi3g33254	3.00E-34
PR-2	Bradi3g33277	3.00E-34
PR-2	Bradi5g26467	7.00E-33
PR-2	Bradi1g55203	1.00E-32
PR-2	Bradi1g61320	8.00E-10
PR-3	Bradi3g32340	4.00E-78
PR-3	Bradi2g47210	4.00E-70
PR-3	Bradi1g29887	3.00E-69
PR-3	Bradi1g29880	6.00E-67
PR-3	Bradi2g47191	7.00E-63
PR-3	Bradi2g26000	5.00E-57
PR-3	Bradi1g76217	8.00E-50
PR-3	Bradi2g11140	3.00E-48
PR-3	Bradi2g36780	6.00E-40
PR-3	Bradi3g40320	6.00E-32
PR-3	Bradi5g14430	2.00E-27
PR-3	Bradi4g34040	1.00E-26
PR-3	Bradi3g48230	2.00E-26
PR-3	Bradi2g26017	9.00E-23
PR-4	Bradi4g14930	2.00E-42
PR-4	Bradi4g14920	1.00E-41
PR-5	Bradi1g33540	2.00E-67
PR-5	Bradi4g34180	4.00E-62
PR-5	Bradi3g04330	2.00E-60
PR-5	Bradi3g42380	1.00E-59
PR-5	Bradi3g40596	4.00E-57
PR-5	Bradi1g68330	1.00E-56
PR-5	Bradi4g36410	3.00E-56
PR-5	Bradi1g69277	3.00E-55
PR-5	Bradi1g68340	5.00E-55
PR-5	Bradi4g36400	2.00E-54
PR-5	Bradi4g09130	3.00E-53
PR-5	Bradi4g03290	7.00E-52
PR-5	Bradi4g05430	1.00E-51
PR-5	Bradi1g30117	3.00E-51
PR-5	Bradi3g21100	2.00E-50
PR-5	Bradi5g27280	4.00E-49
PR-5	Bradi5g00550	1.00E-47
PR-5	Bradi4g05440	2.00E-44
PR-5	Bradi1g13060	8.00E-43

PR-5	Bradi2g54560	2.00E-41
PR-5	Bradi1g13070	6.00E-41
PR-5	Bradi2g01200	4.00E-40
PR-5	Bradi3g26630	2.00E-38
PR-5	Bradi2g01217	2.00E-37
PR-5	Bradi2g01227	1.00E-27
PR-5	Bradi3g07960	2.00E-22
PR-5	Bradi4g09220	3.00E-22
PR-5	Bradi4g04160	2.00E-15
PR-5	Bradi4g04150	4.00E-15
PR-5	Bradi4g03280	6.00E-15
PR-5	Bradi4g04180	2.00E-13
PR-5	Bradi4g03285	3.00E-09
PR-5	Bradi4g09370	1.00E-08
PR-6	Bradi2g35500	3.00E-09
PR-6	Bradi1g09487	5.00E-09
PR-6	Bradi1g46410	6.00E-09
PR-6	Bradi2g39290	5.00E-08
PR-6	Bradi3g36050	7.00E-08
PR-6	Bradi2g35540	1.00E-07
PR-6	Bradi4g40616	4.00E-07
PR-6	Bradi2g39271	5.00E-07
PR-6	Bradi2g39260	2.00E-06
PR-6	Bradi2g39280	2.00E-06
PR-7	Bradi5g18110	4.00E-163
PR-7	Bradi5g18117	6.00E-158
PR-7	Bradi5g24780	8.00E-157
PR-7	Bradi3g31690	1.00E-154
PR-7	Bradi1g77260	2.00E-154
PR-7	Bradi5g18130	1.00E-151
PR-7	Bradi5g24500	4.00E-151
PR-7	Bradi3g51070	4.00E-149
PR-7	Bradi3g19300	8.00E-146
PR-7	Bradi2g27640	2.00E-139
PR-7	Bradi1g75550	3.00E-138
PR-7	Bradi2g07730	4.00E-137
PR-7	Bradi3g19320	6.00E-137
PR-7	Bradi5g18100	1.00E-134
PR-7	Bradi4g11640	5.00E-132
PR-7	Bradi1g17320	1.00E-130
PR-7	Bradi1g07840	4.00E-130

PR-7	Bradi1g68270	5.00E-128
PR-7	Bradi5g18910	1.00E-126
PR-7	Bradi3g37457	7.00E-125
PR-7	Bradi1g14860	2.00E-124
PR-7	Bradi1g17330	6.00E-122
PR-7	Bradi4g31360	2.00E-120
PR-7	Bradi2g33990	2.00E-119
PR-7	Bradi5g00840	3.00E-119
PR-7	Bradi1g17350	2.00E-118
PR-7	Bradi4g33237	2.00E-117
PR-7	Bradi3g57140	9.00E-117
PR-7	Bradi4g41420	1.00E-116
PR-7	Bradi3g57130	2.00E-115
PR-7	Bradi2g24220	6.00E-115
PR-7	Bradi5g10210	8.00E-114
PR-7	Bradi2g48740	2.00E-113
PR-7	Bradi3g20580	7.00E-113
PR-7	Bradi4g24790	5.00E-112
PR-7	Bradi1g36242	8.00E-112
PR-7	Bradi3g07280	4.00E-109
PR-7	Bradi1g07700	7.00E-109
PR-7	Bradi2g56180	4.00E-107
PR-7	Bradi2g51440	1.00E-106
PR-7	Bradi4g36000	5.00E-104
PR-7	Bradi1g53630	2.00E-101
PR-7	Bradi1g54830	9.00E-97
PR-7	Bradi1g08451	1.00E-95
PR-7	Bradi3g04690	2.00E-91
PR-7	Bradi2g10727	1.00E-88
PR-7	Bradi3g10037	8.00E-87
PR-7	Bradi1g08670	1.00E-86
PR-7	Bradi1g74547	7.00E-85
PR-7	Bradi5g03190	1.00E-84
PR-7	Bradi5g03790	3.00E-82
PR-7	Bradi3g10058	1.00E-80
PR-7	Bradi5g23480	9.00E-79
PR-7	Bradi3g10030	1.00E-75
PR-7	Bradi2g51130	4.00E-73
PR-7	Bradi1g34087	7.00E-71
PR-7	Bradi5g03780	7.00E-67
PR-7	Bradi5g17320	1.00E-63

PR-7	Bradi3g04674	1.00E-41
PR-7	Bradi3g10044	4.00E-35
PR-8	Bradi1g52625	1.00E-83
PR-8	Bradi2g55630	7.00E-83
PR-8	Bradi2g55620	6.00E-81
PR-8	Bradi2g55610	9.00E-81
PR-8	Bradi2g45610	1.00E-71
PR-8	Bradi2g47067	7.00E-70
PR-8	Bradi2g47171	4.00E-67
PR-8	Bradi2g43755	7.00E-66
PR-8	Bradi4g40120	4.00E-38
PR-8	Bradi4g40110	5.00E-37
PR-8	Bradi2g47196	4.00E-34
PR-8	Bradi4g09417	7.00E-32
PR-8	Bradi4g09430	9.00E-30
PR-8	Bradi4g07560	8.00E-23
PR-9	Bradi1g68900	7.00E-87
PR-9	Bradi1g27920	1.00E-81
PR-9	Bradi2g40590	4.00E-81
PR-9	Bradi5g10070	8.00E-81
PR-9	Bradi1g68927	2.00E-80
PR-9	Bradi4g25660	5.00E-80
PR-9	Bradi1g68887	1.00E-79
PR-9	Bradi2g09660	9.00E-77
PR-9	Bradi2g09690	1.00E-76
PR-9	Bradi4g05230	1.00E-76
PR-9	Bradi4g44530	2.00E-76
PR-9	Bradi3g09080	2.00E-75
PR-9	Bradi1g38297	4.00E-75
PR-9	Bradi1g57247	6.00E-75
PR-9	Bradi1g38290	7.00E-75
PR-9	Bradi2g09680	1.00E-74
PR-9	Bradi1g38310	4.00E-74
PR-9	Bradi3g09087	8.00E-74
PR-9	Bradi2g09650	3.00E-73
PR-9	Bradi1g17860	3.00E-73
PR-9	Bradi1g63060	5.00E-73
PR-9	Bradi1g27910	1.00E-72
PR-9	Bradi1g17877	6.00E-72
PR-9	Bradi4g05190	1.00E-71
PR-9	Bradi3g09140	3.00E-71

PR-9	Bradi1g17870	4.00E-71
PR-9	Bradi5g24200	7.00E-71
PR-9	Bradi1g43680	5.00E-70
PR-9	Bradi3g09120	6.00E-70
PR-9	Bradi3g09100	1.00E-69
PR-9	Bradi4g44510	2.00E-69
PR-9	Bradi1g41115	5.00E-69
PR-9	Bradi1g38350	6.00E-68
PR-9	Bradi4g32800	6.00E-68
PR-9	Bradi3g09130	6.00E-68
PR-9	Bradi3g20130	8.00E-68
PR-9	Bradi1g39190	6.00E-67
PR-9	Bradi1g17840	5.00E-66
PR-9	Bradi1g17790	2.00E-65
PR-9	Bradi4g32810	2.00E-65
PR-9	Bradi2g04490	6.00E-65
PR-9	Bradi5g19857	1.00E-64
PR-9	Bradi1g63067	2.00E-64
PR-9	Bradi1g42900	2.00E-64
PR-9	Bradi2g20830	3.00E-64
PR-9	Bradi2g20850	7.00E-64
PR-9	Bradi2g06497	2.00E-63
PR-9	Bradi2g20840	4.00E-63
PR-9	Bradi2g12228	1.00E-62
PR-9	Bradi2g34717	5.00E-62
PR-9	Bradi1g17850	9.00E-62
PR-9	Bradi3g33940	7.00E-61
PR-9	Bradi1g77140	2.00E-59
PR-9	Bradi3g41340	2.00E-59
PR-9	Bradi2g37010	3.00E-59
PR-9	Bradi2g37020	5.00E-59
PR-9	Bradi4g27680	5.00E-59
PR-9	Bradi2g13190	6.00E-59
PR-9	Bradi1g15600	6.00E-59
PR-9	Bradi3g13590	7.00E-59
PR-9	Bradi2g46050	2.00E-58
PR-9	Bradi2g09600	2.00E-58
PR-9	Bradi3g60880	2.00E-58
PR-9	Bradi3g59660	3.00E-58
PR-9	Bradi1g59537	2.00E-57
PR-9	Bradi1g44800	5.00E-57

PR-9	Bradi3g32130	1.00E-56
PR-9	Bradi2g11307	5.00E-56
PR-9	Bradi5g27687	5.00E-56
PR-9	Bradi1g44790	6.00E-56
PR-9	Bradi1g07790	1.00E-54
PR-9	Bradi1g59550	2.00E-54
PR-9	Bradi2g52077	3.00E-54
PR-9	Bradi3g04717	6.00E-54
PR-9	Bradi2g12180	8.00E-54
PR-9	Bradi2g10150	8.00E-54
PR-9	Bradi2g12192	1.00E-53
PR-9	Bradi2g48050	1.00E-53
PR-9	Bradi2g12204	1.00E-53
PR-9	Bradi1g59520	1.00E-53
PR-9	Bradi5g27130	1.00E-53
PR-9	Bradi2g12170	2.00E-53
PR-9	Bradi5g14650	2.00E-53
PR-9	Bradi5g22650	1.00E-52
PR-9	Bradi3g33780	4.00E-52
PR-9	Bradi2g37000	5.00E-52
PR-9	Bradi5g12710	5.00E-52
PR-9	Bradi5g24650	7.00E-52
PR-9	Bradi1g77130	9.00E-52
PR-9	Bradi2g12216	5.00E-51
PR-9	Bradi1g58997	9.00E-51
PR-9	Bradi3g32110	1.00E-50
PR-9	Bradi2g20820	2.00E-50
PR-9	Bradi1g32870	2.00E-50
PR-9	Bradi1g33740	7.00E-50
PR-9	Bradi2g11300	1.00E-49
PR-9	Bradi5g27170	4.00E-49
PR-9	Bradi1g07780	1.00E-48
PR-9	Bradi1g74882	5.00E-48
PR-9	Bradi3g55850	7.00E-48
PR-9	Bradi1g26870	1.00E-47
PR-9	Bradi1g61550	1.00E-47
PR-9	Bradi5g27200	2.00E-47
PR-9	Bradi4g22660	3.00E-47
PR-9	Bradi4g40680	6.00E-47
PR-9	Bradi2g37040	9.00E-47
PR-9	Bradi2g11295	2.00E-46

PR-9	Bradi1g61540	4.00E-46
PR-9	Bradi5g27150	3.00E-45
PR-9	Bradi4g05980	1.00E-44
PR-9	Bradi1g61530	2.00E-44
PR-9	Bradi2g17120	3.00E-44
PR-9	Bradi5g27160	3.00E-44
PR-9	Bradi1g20000	5.00E-44
PR-9	Bradi3g10470	5.00E-44
PR-9	Bradi1g41900	8.00E-44
PR-9	Bradi4g40190	3.00E-43
PR-9	Bradi1g20005	6.00E-43
PR-9	Bradi5g00690	8.00E-43
PR-9	Bradi3g10460	9.00E-43
PR-9	Bradi1g33730	1.00E-42
PR-9	Bradi3g29500	2.00E-42
PR-9	Bradi5g27220	3.00E-42
PR-9	Bradi5g27210	7.00E-42
PR-9	Bradi1g20020	2.00E-41
PR-9	Bradi2g38660	4.00E-41
PR-9	Bradi2g11320	6.00E-41
PR-9	Bradi1g19980	3.00E-40
PR-9	Bradi1g20026	9.00E-38
PR-9	Bradi1g20010	9.00E-37
PR-9	Bradi1g20032	3.00E-35
PR-9	Bradi1g19990	3.00E-34
PR-9	Bradi2g37060	3.00E-32
PR-9	Bradi2g37067	2.00E-30
PR-9	Bradi2g37047	2.00E-29
PR-9	Bradi2g38690	1.00E-24
PR-9	Bradi2g38670	1.00E-24
PR-9	Bradi2g37080	1.00E-21
PR-9	Bradi2g38680	2.00E-21
PR-9	Bradi1g26215	2.00E-21
PR-9	Bradi2g38685	3.00E-20
PR-9	Bradi2g38720	1.00E-19
PR-9	Bradi2g37090	1.00E-19
PR-9	Bradi2g38700	1.00E-18
PR-9	Bradi3g45700	7.00E-14
PR-9	Bradi4g41180	2.00E-12
PR-9	Bradi5g10490	4.00E-12
PR-9	Bradi3g40330	1.00E-11

PR-9	Bradi4g25650	2.00E-10
PR-10	Bradi1g64920	5.00E-16
PR-10	Bradi1g64910	1.00E-15
PR-10	Bradi1g64890	2.00E-09
PR-10	Bradi3g48400	1.00E-08
PR-10	Bradi1g64880	2.00E-07
PR-10	Bradi4g05040	4.00E-06
PR-11	Bradi5g07230	2.00E-31
PR-13	Bradi1g57337	2.00E-08
PR-13	Bradi1g57302	1.00E-06
PR-13	Bradi1g57400	2.00E-06
PR-14	Bradi4g44410	5.00E-20
PR-14	Bradi1g21870	1.00E-17
PR-14	Bradi4g25750	2.00E-17
PR-14	Bradi4g44400	1.00E-15
PR-14	Bradi1g39120	4.00E-14
PR-14	Bradi2g53570	1.00E-11
PR-14	Bradi2g22284	8.00E-11
PR-14	Bradi2g07140	5.00E-10
PR-14	Bradi2g22278	8.00E-08
PR-15/16	Bradi1g11920	1.00E-113
PR-15/16	Bradi1g11930	1.00E-111
PR-15/16	Bradi2g47680	2.00E-51
PR-15/16	Bradi2g47690	1.00E-50
PR-15/16	Bradi2g11050	7.00E-50
PR-15/16	Bradi2g08790	1.00E-47
PR-15/16	Bradi1g04907	2.00E-47
PR-15/16	Bradi3g43650	2.00E-47
PR-15/16	Bradi2g21010	4.00E-46
PR-15/16	Bradi4g06080	4.00E-46
PR-15/16	Bradi2g60870	1.00E-45
PR-15/16	Bradi3g43660	7.00E-45
PR-15/16	Bradi3g17330	2.00E-44
PR-15/16	Bradi3g43490	5.00E-44
PR-15/16	Bradi3g15220	1.00E-43
PR-15/16	Bradi3g15210	4.00E-43
PR-15/16	Bradi3g15250	4.00E-43
PR-15/16	Bradi3g15200	5.00E-43
PR-15/16	Bradi3g15190	8.00E-43
PR-15/16	Bradi3g17316	1.00E-42
PR-15/16	Bradi5g21910	2.00E-42

PR-15/16	Bradi3g15240	2.00E-42
PR-15/16	Bradi3g15230	9.00E-42
PR-15/16	Bradi2g60860	4.00E-40
PR-15/16	Bradi3g15260	1.00E-39
PR-15/16	Bradi3g17308	8.00E-39
PR-15/16	Bradi2g33160	7.00E-35
PR-15/16	Bradi3g44880	3.00E-34
PR-15/16	Bradi3g37670	1.00E-28
PR-15/16	Bradi3g37680	5.00E-28

PR-15/16	Bradi4g38550	5.00E-24
PR-15/16	Bradi4g38570	2.00E-23
PR-15/16	Bradi3g44300	3.00E-23
PR-15/16	Bradi4g38560	2.00E-20
PR-15/16	Bradi4g38575	7.00E-20
PR-15/16	Bradi2g00792	9.00E-20
PR-15/16	Bradi4g17030	1.00E-08
PR-17	Bradi3g29710	6.00E-74
