# Supplementary material for: Theobroma cacao L. pathogenesis-related gene tandem array members show diverse expression dynamics in response to pathogen colonization
Source: BMC Genomics. 2016 May 17;17:363. doi: 10.1186/s12864-016-2693-3 (PMC4869279; doi:10.1186/s12864-016-2693-3)
Supplement: Additional file 6: Table S6. — Gene IDs and BLASTp E-values for Oryza sativa PR loci. (PDF 4169 kb) [file 12864_2016_2693_MOESM6_ESM.pdf]

Supplemental Table S6 - Gene IDs and BLASTp E-value for *Oryza sativa* PR genes

PR Gene Class	Gene ID	E-value
PR-1	LOC_Os07g03730	9.00E-45
PR-1	LOC_Os07g03710	1.00E-43
PR-1	LOC_Os10g11500	3.00E-43
PR-1	LOC_Os01g28450	3.00E-42
PR-1	LOC_Os01g28500	2.00E-40
PR-1	LOC_Os06g24290	1.00E-39
PR-1	LOC_Os02g27310	8.00E-37
PR-1	LOC_Os02g54540	1.00E-34
PR-1	LOC_Os07g03740	2.00E-34
PR-1	LOC_Os12g43700	1.00E-33
PR-1	LOC_Os07g03279	2.00E-33
PR-1	LOC_Os07g03319	2.00E-33
PR-1	LOC_Os07g03368	2.00E-33
PR-1	LOC_Os07g03409	2.00E-33
PR-1	LOC_Os07g03458	2.00E-33
PR-1	LOC_Os07g03499	2.00E-33
PR-1	LOC_Os07g03288	7.00E-32
PR-1	LOC_Os07g03377	7.00E-32
PR-1	LOC_Os07g03467	7.00E-32
PR-1	LOC_Os07g03590	7.00E-32
PR-1	LOC_Os05g51680	1.00E-31
PR-1	LOC_Os07g03690	2.00E-31
PR-1	LOC_Os07g03750	1.00E-30
PR-1	LOC_Os07g03580	2.00E-29
PR-1	LOC_Os07g03600	4.00E-28
PR-1	LOC_Os02g54530	2.00E-27
PR-1	LOC_Os07g03680	4.00E-27
PR-1	LOC_Os02g54560	1.00E-26
PR-1	LOC_Os07g14030	1.00E-26
PR-1	LOC_Os07g14070	1.00E-26
PR-1	LOC_Os02g27300	4.00E-26
PR-1	LOC_Os07g03610	2.00E-25
PR-1	LOC_Os04g22230	5.00E-25
PR-1	LOC_Os07g03620	1.00E-24
PR-1	LOC_Os04g22220	2.00E-24
PR-1	LOC_Os04g22210	3.00E-24
PR-1	LOC_Os02g54570	2.00E-22
PR-1	LOC_Os05g51660	2.00E-21
PR-1	LOC_Os04g22330	5.00E-10
PR-2	LOC_Os01g71340	2.00E-82
PR-2	LOC_Os01g71810	2.00E-81
PR-2	LOC_Os01g71820	2.00E-80
PR-2	LOC_Os01g71830	5.00E-80
PR-2	LOC_Os01g71400	4.00E-78
PR-2	LOC_Os01g71930	6.00E-77
PR-2	LOC_Os01g71670	7.00E-75
PR-2	LOC_Os01g71680	2.00E-74
PR-2	LOC_Os01g71860	2.00E-73
PR-2	LOC_Os01g71690	1.00E-72
PR-2	LOC_Os01g71410	1.00E-71
PR-2	LOC_Os01g71380	3.00E-71
PR-2	LOC_Os05g31140	6.00E-71
PR-2	LOC_Os05g41610	1.00E-69
PR-2	LOC_Os01g58730	2.00E-69
PR-2	LOC_Os01g71350	2.00E-69
PR-2	LOC_Os01g51570	1.00E-64
PR-2	LOC_Os01g53750	5.00E-61
PR-2	LOC_Os01g71474	5.00E-59
PR-2	LOC_Os03g14210	3.00E-58
PR-2	LOC_Os04g33640	3.00E-58
PR-2	LOC_Os09g36280	5.00E-58
PR-2	LOC_Os03g12140	6.00E-54
PR-2	LOC_Os06g34020	4.00E-53
PR-2	LOC_Os02g33000	5.00E-53
PR-2	LOC_Os03g12620	3.00E-52
PR-2	LOC_Os01g64170	4.00E-52
PR-2	LOC_Os02g10660	4.00E-52
PR-2	LOC_Os02g53200	5.00E-52
PR-2	LOC_Os03g46660	1.00E-51

PR-2	LOC_Os10g07290	1.00E-51
PR-2	LOC_Os06g40490	2.00E-51
PR-2	LOC_Os06g04080	3.00E-50
PR-2	LOC_Os07g35350	5.00E-48
PR-2	LOC_Os03g57880	2.00E-46
PR-2	LOC_Os02g04670	1.00E-45
PR-2	LOC_Os06g39060	1.00E-45
PR-2	LOC_Os08g41410	1.00E-45
PR-2	LOC_Os07g38930	2.00E-44
PR-2	LOC_Os01g71650	3.00E-44
PR-2	LOC_Os08g12800	4.00E-44
PR-2	LOC_Os07g07340	6.00E-44
PR-2	LOC_Os11g36940	1.00E-43
PR-2	LOC_Os03g62860	2.00E-42
PR-2	LOC_Os11g47820	2.00E-42
PR-2	LOC_Os07g13580	3.00E-42
PR-2	LOC_Os03g51240	1.00E-41
PR-2	LOC_Os07g35480	1.00E-41
PR-2	LOC_Os07g35560	3.00E-41
PR-2	LOC_Os03g40330	5.00E-41
PR-2	LOC_Os07g35510	2.00E-40
PR-2	LOC_Os03g18520	4.00E-40
PR-2	LOC_Os03g45390	4.00E-40
PR-2	LOC_Os08g14700	1.00E-39
PR-2	LOC_Os07g35520	3.00E-39
PR-2	LOC_Os09g09980	1.00E-38
PR-2	LOC_Os09g32550	4.00E-38
PR-2	LOC_Os08g23720	5.00E-38
PR-2	LOC_Os07g32600	2.00E-36
PR-2	LOC_Os05g37130	5.00E-35
PR-2	LOC_Os05g45860	3.00E-33
PR-2	LOC_Os03g27980	5.00E-33
PR-2	LOC_Os03g56130	5.00E-17
PR-2	LOC_Os07g41014	3.00E-14
PR-2	LOC_Os03g22530	2.00E-13
PR-2	LOC_Os03g25790	3.00E-09
PR-3	LOC_Os10g39680	7.00E-77
PR-3	LOC_Os06g51060	2.00E-71
PR-3	LOC_Os06g51050	5.00E-71
PR-3	LOC_Os05g33130	5.00E-68
PR-3	LOC_Os03g30470	3.00E-66
PR-3	LOC_Os03g04060	2.00E-65
PR-3	LOC_Os05g33140	5.00E-65
PR-3	LOC_Os01g18400	4.00E-49
PR-3	LOC_Os05g33150	7.00E-49
PR-3	LOC_Os05g04690	3.00E-44
PR-3	LOC_Os10g39700	3.00E-32
PR-3	LOC_Os08g41100	1.00E-31
PR-3	LOC_Os02g39330	2.00E-27
PR-3	LOC_Os09g32080	2.00E-27
PR-3	LOC_Os04g41620	1.00E-26
PR-3	LOC_Os04g41680	7.00E-26
PR-4	LOC_Os11g37970	1.00E-43
PR-4	LOC_Os11g37950	4.00E-42
PR-4	LOC_Os11g37960	1.00E-40
PR-4	LOC_Os11g37940	6.00E-39
PR-5	LOC_Os06g47600	3.00E-68
PR-5	LOC_Os09g32280	8.00E-65
PR-5	LOC_Os08g43510	2.00E-64
PR-5	LOC_Os10g05660	2.00E-62
PR-5	LOC_Os09g36580	3.00E-61
PR-5	LOC_Os11g47944	5.00E-59
PR-5	LOC_Os04g59370	6.00E-59
PR-5	LOC_Os03g14030	1.00E-57
PR-5	LOC_Os09g36560	3.00E-57
PR-5	LOC_Os03g14050	2.00E-56
PR-5	LOC_Os10g05600	7.00E-55
PR-5	LOC_Os03g13070	9.00E-55
PR-5	LOC_Os06g50240	2.00E-53
PR-5	LOC_Os08g40600	3.00E-52
PR-5	LOC_Os07g23470	1.00E-50
PR-5	LOC_Os01g62260	1.00E-44
PR-5	LOC_Os03g45960	2.00E-44
PR-5	LOC_Os07g23730	2.00E-44
PR-5	LOC_Os03g46070	4.00E-44
PR-5	LOC_Os10g27280	1.00E-43
PR-5	LOC_Os12g43490	3.00E-43
PR-5	LOC_Os12g43450	2.00E-39
PR-5	LOC_Os01g02310	5.00E-34
PR-5	LOC_Os03g46060	3.00E-31
PR-5	LOC_Os12g43380	3.00E-25
PR-5	LOC_Os12g43430	3.00E-25

PR-5	LOC_Os12g43440	5.00E-24
PR-5	LOC_Os12g43390	3.00E-21
PR-5	LOC_Os02g02200	9.00E-19
PR-5	LOC_Os11g47680	1.00E-17
PR-5	LOC_Os12g38120	6.00E-15
PR-5	LOC_Os12g43410	1.00E-12
PR-5	LOC_Os10g38100	8.00E-11
PR-5	LOC_Os07g04730	2.00E-08
PR-5	LOC_Os12g38150	2.00E-08
PR-5	LOC_Os12g38170	3.00E-08
PR-5	LOC_Os11g47670	3.00E-07
PR-6	LOC_Os12g36210	3.00E-08
PR-6	LOC_Os05g01920	5.00E-07
PR-6	LOC_Os11g17790	9.00E-07
PR-6	LOC_Os05g25630	8.00E-06
PR-7	LOC_Os10g38080	6.00E-158
PR-7	LOC_Os04g47160	2.00E-156
PR-7	LOC_Os03g02750	4.00E-153
PR-7	LOC_Os12g23980	2.00E-145
PR-7	LOC_Os02g44590	6.00E-144
PR-7	LOC_Os03g04950	3.00E-142
PR-7	LOC_Os07g48650	1.00E-141
PR-7	LOC_Os03g55350	4.00E-138
PR-7	LOC_Os04g47150	5.00E-135
PR-7	LOC_Os05g30580	2.00E-133
PR-7	LOC_Os03g40830	1.00E-131
PR-7	LOC_Os08g35090	2.00E-128
PR-7	LOC_Os09g26920	2.00E-128
PR-7	LOC_Os03g13930	5.00E-126
PR-7	LOC_Os02g53860	3.00E-125
PR-7	LOC_Os04g48416	8.00E-123
PR-7	LOC_Os03g31630	3.00E-122
PR-7	LOC_Os05g36010	6.00E-122
PR-7	LOC_Os09g30250	4.00E-120
PR-7	LOC_Os04g10360	2.00E-118
PR-7	LOC_Os06g40700	5.00E-118
PR-7	LOC_Os04g35140	2.00E-114
PR-7	LOC_Os07g39020	3.00E-114
PR-7	LOC_Os10g25450	8.00E-114
PR-7	LOC_Os02g10520	4.00E-113
PR-7	LOC_Os02g53970	1.00E-112

PR-7	LOC_Os01g64860	8.00E-111
PR-7	LOC_Os08g23740	1.00E-109
PR-7	LOC_Os01g52750	4.00E-107
PR-7	LOC_Os01g64850	4.00E-106
PR-7	LOC_Os06g41880	1.00E-105
PR-7	LOC_Os09g36110	1.00E-105
PR-7	LOC_Os02g53910	1.00E-104
PR-7	LOC_Os01g58240	7.00E-95
PR-7	LOC_Os09g30458	8.00E-94
PR-7	LOC_Os04g03050	1.00E-92
PR-7	LOC_Os03g06290	1.00E-89
PR-7	LOC_Os04g03100	2.00E-88
PR-7	LOC_Os02g53850	3.00E-87
PR-7	LOC_Os04g02980	5.00E-86
PR-7	LOC_Os01g58290	8.00E-85
PR-7	LOC_Os02g16940	2.00E-84
PR-7	LOC_Os01g58270	2.00E-82
PR-7	LOC_Os02g17090	4.00E-81
PR-7	LOC_Os04g03796	1.00E-78
PR-7	LOC_Os02g17150	4.00E-78
PR-7	LOC_Os01g56320	2.00E-74
PR-7	LOC_Os04g03810	1.00E-73
PR-7	LOC_Os01g58280	2.00E-72
PR-7	LOC_Os06g48650	1.00E-71
PR-7	LOC_Os11g15520	1.00E-68
PR-7	LOC_Os04g45960	1.00E-67
PR-7	LOC_Os02g17080	7.00E-65
PR-7	LOC_Os02g17060	8.00E-58
PR-7	LOC_Os01g50680	1.00E-49
PR-7	LOC_Os02g17000	2.00E-47
PR-7	LOC_Os04g03850	3.00E-36
PR-7	LOC_Os10g41880	9.00E-13
PR-8	LOC_Os01g64110	4.00E-87
PR-8	LOC_Os07g19040	3.00E-86
PR-8	LOC_Os01g64100	3.00E-80
PR-8	LOC_Os01g49320	6.00E-77
PR-8	LOC_Os01g47070	1.00E-75
PR-8	LOC_Os01g19750	1.00E-74
PR-8	LOC_Os04g27980	2.00E-54
PR-8	LOC_Os07g23850	1.00E-49
PR-8	LOC_Os11g47600	3.00E-39

PR-8	LOC_Os11g47610	2.00E-37
PR-8	LOC_Os11g47550	2.00E-36
PR-8	LOC_Os11g47580	4.00E-36
PR-8	LOC_Os11g47590	5.00E-36
PR-8	LOC_Os11g47530	1.00E-34
PR-8	LOC_Os11g47500	4.00E-33
PR-8	LOC_Os11g47520	6.00E-33
PR-8	LOC_Os11g47510	8.00E-33
PR-8	LOC_Os05g15850	6.00E-32
PR-8	LOC_Os11g47560	1.00E-31
PR-8	LOC_Os11g47570	1.00E-31
PR-8	LOC_Os07g43820	3.00E-31
PR-8	LOC_Os05g15770	1.00E-29
PR-8	LOC_Os05g15920	2.00E-28
PR-8	LOC_Os08g40680	2.00E-28
PR-8	LOC_Os08g40740	7.00E-27
PR-8	LOC_Os05g15880	4.00E-26
PR-8	LOC_Os08g40690	3.00E-23
PR-8	LOC_Os06g25010	3.00E-20
PR-8	LOC_Os07g01770	5.00E-16
PR-9	LOC_Os03g13200	2.00E-89
PR-9	LOC_Os03g13180	8.00E-88
PR-9	LOC_Os03g32050	2.00E-84
PR-9	LOC_Os03g13210	7.00E-84
PR-9	LOC_Os01g36240	4.00E-82
PR-9	LOC_Os10g02070	5.00E-80
PR-9	LOC_Os04g34630	3.00E-79
PR-9	LOC_Os01g15830	1.00E-78
PR-9	LOC_Os07g48030	6.00E-78
PR-9	LOC_Os11g02130	6.00E-78
PR-9	LOC_Os06g35520	1.00E-77
PR-9	LOC_Os12g02080	7.00E-77
PR-9	LOC_Os06g35480	1.00E-75
PR-9	LOC_Os10g02040	5.00E-75
PR-9	LOC_Os07g48020	2.00E-74
PR-9	LOC_Os02g14180	4.00E-74
PR-9	LOC_Os06g35490	1.00E-73
PR-9	LOC_Os04g55740	5.00E-73
PR-9	LOC_Os01g10850	3.00E-72
PR-9	LOC_Os07g48050	5.00E-72
PR-9	LOC_Os07g47990	1.00E-71
PR-9	LOC_Os06g33080	2.00E-71
PR-9	LOC_Os01g15810	3.00E-71
PR-9	LOC_Os02g14160	4.00E-69
PR-9	LOC_Os06g33100	3.00E-68
PR-9	LOC_Os03g22010	5.00E-68
PR-9	LOC_Os02g14460	6.00E-68
PR-9	LOC_Os07g48010	1.00E-67
PR-9	LOC_Os02g14430	2E-67
PR-9	LOC_Os08g20730	7.00E-67
PR-9	LOC_Os02g14440	8.00E-67
PR-9	LOC_Os06g16350	2.00E-66
PR-9	LOC_Os06g32960	3.00E-66
PR-9	LOC_Os09g29490	4.00E-66
PR-9	LOC_Os03g22020	7.00E-65
PR-9	LOC_Os02g14170	3.00E-64
PR-9	LOC_Os06g32990	5.00E-64
PR-9	LOC_Os07g48040	5.00E-64
PR-9	LOC_Os06g32980	6.00E-64
PR-9	LOC_Os05g06970	1.00E-63
PR-9	LOC_Os12g02060	7.00E-62
PR-9	LOC_Os03g02939	1.00E-61
PR-9	LOC_Os08g42030	4.00E-61
PR-9	LOC_Os01g73220	5.00E-61
PR-9	LOC_Os05g41990	1.00E-60
PR-9	LOC_Os03g02920	2.00E-60
PR-9	LOC_Os07g01420	2.00E-60
PR-9	LOC_Os07g48060	6.00E-60
PR-9	LOC_Os06g46799	8.00E-60
PR-9	LOC_Os11g43980	1.00E-59
PR-9	LOC_Os01g07770	3.00E-59
PR-9	LOC_Os06g29470	3.00E-59
PR-9	LOC_Os06g20150	9.00E-59
PR-9	LOC_Os03g55410	1.00E-58
PR-9	LOC_Os04g53640	1.00E-58
PR-9	LOC_Os04g56180	2.00E-58
PR-9	LOC_Os07g01370	2.00E-58
PR-9	LOC_Os10g39170	2.00E-58
PR-9	LOC_Os09g32964	6.00E-58
PR-9	LOC_Os01g16450	1.00E-57
PR-9	LOC_Os01g73200	2.00E-57
PR-9	LOC_Os02g58720	3.00E-57

PR-9	LOC_Os07g01400	3.00E-57
PR-9	LOC_Os11g02100	1.00E-56
PR-9	LOC_Os08g02110	2.00E-56
PR-9	LOC_Os02g50770	9.00E-56
PR-9	LOC_Os07g34710	1.00E-55
PR-9	LOC_Os01g22336	2.00E-55
PR-9	LOC_Os05g04490	2.00E-55
PR-9	LOC_Os01g57730	1.00E-54
PR-9	LOC_Os04g59150	3.00E-54
PR-9	LOC_Os01g73170	5.00E-54
PR-9	LOC_Os01g22370	6.00E-54
PR-9	LOC_Os01g22249	3.00E-53
PR-9	LOC_Os07g31610	1.00E-52
PR-9	LOC_Os03g36560	2.00E-52
PR-9	LOC_Os07g01410	3.00E-52
PR-9	LOC_Os01g28030	5.00E-52
PR-9	LOC_Os06g48030	5.00E-52
PR-9	LOC_Os07g02440	7.00E-52
PR-9	LOC_Os10g41720	4.00E-51
PR-9	LOC_Os10g39160	5.00E-51
PR-9	LOC_Os12g08920	7.00E-51
PR-9	LOC_Os04g01550	1.00E-50
PR-9	LOC_Os04g39100	1.00E-50
PR-9	LOC_Os11g10460	1.00E-50
PR-9	LOC_Os07g34670	2.00E-50
PR-9	LOC_Os06g33090	3.00E-50
PR-9	LOC_Os03g55420	4E-50
PR-9	LOC_Os05g04470	5.00E-50
PR-9	LOC_Os06g13050	6.00E-50
PR-9	LOC_Os05g04450	7.00E-50
PR-9	LOC_Os03g25360	8.00E-50
PR-9	LOC_Os03g25330	9.00E-50
PR-9	LOC_Os12g34524	1.00E-49
PR-9	LOC_Os02g06630	3.00E-49
PR-9	LOC_Os01g51550	4.00E-49
PR-9	LOC_Os01g22230	1.00E-48
PR-9	LOC_Os03g05770	1.00E-48
PR-9	LOC_Os07g44590	2.00E-48
PR-9	LOC_Os03g25370	3.00E-48
PR-9	LOC_Os05g04500	7.00E-48
PR-9	LOC_Os05g42000	2.00E-47

PR-9	LOC_Os06g48010	2.00E-47
PR-9	LOC_Os01g22352	5.00E-47
PR-9	LOC_Os09g15510	1.00E-46
PR-9	LOC_Os06g48000	3.00E-46
PR-9	LOC_Os09g15500	3.00E-46
PR-9	LOC_Os03g25320	5.00E-46
PR-9	LOC_Os04g59260	1.00E-45
PR-9	LOC_Os01g73190	4.00E-45
PR-9	LOC_Os04g59160	4.00E-45
PR-9	LOC_Os03g25280	5.00E-45
PR-9	LOC_Os03g25300	5.00E-45
PR-9	LOC_Os07g06300	7.00E-45
PR-9	LOC_Os07g06190	8.00E-45
PR-9	LOC_Os07g44550	1.00E-44
PR-9	LOC_Os04g59200	2.00E-44
PR-9	LOC_Os06g27850	2.00E-44
PR-9	LOC_Os04g59210	3.00E-44
PR-9	LOC_Os03g25340	4.00E-44
PR-9	LOC_Os01g19020	4.00E-43
PR-9	LOC_Os06g48020	1.00E-42
PR-9	LOC_Os07g44460	1.00E-42
PR-9	LOC_Os05g04380	6.00E-42
PR-9	LOC_Os07g44480	2.00E-41
PR-9	LOC_Os07g44499	6.00E-41
PR-9	LOC_Os01g18930	1.00E-40
PR-9	LOC_Os04g59190	4.00E-40
PR-9	LOC_Os07g49360	5.00E-40
PR-9	LOC_Os01g18970	5.00E-39
PR-9	LOC_Os07g06175	6.00E-39
PR-9	LOC_Os05g04440	8.00E-36
PR-9	LOC_Os01g18950	3.00E-35
PR-9	LOC_Os01g18910	4.00E-35
PR-9	LOC_Os05g14260	6.00E-33
PR-9	LOC_Os05g04410	9.00E-31
PR-9	LOC_Os10g01760	2.00E-30
PR-9	LOC_Os07g06380	2.00E-29
PR-9	LOC_Os04g04750	2.00E-26
PR-9	LOC_Os01g18890	3.00E-26
PR-9	LOC_Os04g06860	1.00E-14
PR-9	LOC_Os07g01380	3.00E-14
PR-9	LOC_Os12g07830	1.00E-13

PR-9	LOC_Os12g07820	4.00E-13
PR-9	LOC_Os02g34810	7.00E-13
PR-9	LOC_Os04g35520	3.00E-12
PR-9	LOC_Os08g41090	7.00E-12
PR-9	LOC_Os12g09460	2.00E-11
PR-9	LOC_Os08g43560	3.00E-09
PR-9	LOC_Os01g18900	2.00E-07
PR-10	LOC_Os03g18850	1.00E-18
PR-10	LOC_Os04g50700	3.00E-07
PR-10	LOC_Os12g36880	3.00E-06
PR-10	LOC_Os04g50710	3.00E-06
PR-11	LOC_Os11g27400	2.00E-52
PR-11	LOC_Os04g30770	4.00E-49
PR-13	LOC_Os06g31890	7.00E-14
PR-13	LOC_Os06g32160	7.00E-14
PR-13	LOC_Os06g32240	7.00E-14
PR-13	LOC_Os06g31800	1.00E-13
PR-13	LOC_Os06g31930	2.00E-13
PR-13	LOC_Os06g31960	2.00E-13
PR-13	LOC_Os06g32020	2.00E-13
PR-13	LOC_Os06g31280	9.00E-13
PR-13	LOC_Os06g32350	1.00E-12
PR-13	LOC_Os06g32550	6.00E-12
PR-13	LOC_Os06g32600	2.00E-11
PR-14	LOC_Os11g24070	2.00E-22
PR-14	LOC_Os11g02369	8.00E-21
PR-14	LOC_Os12g02310	8.00E-21
PR-14	LOC_Os11g02389	1.00E-20
PR-14	LOC_Os12g02320	2.00E-20
PR-14	LOC_Os11g02400	2.00E-17
PR-14	LOC_Os12g02330	2.00E-16
PR-14	LOC_Os12g02340	3.00E-16
PR-14	LOC_Os06g34840	8.00E-14
PR-14	LOC_Os11g02350	1.00E-13
PR-14	LOC_Os12g02300	1.00E-13
PR-14	LOC_Os05g40010	4.00E-13
PR-14	LOC_Os01g60740	2.00E-11
PR-14	LOC_Os08g03690	4.00E-11
PR-14	LOC_Os02g24720	1.00E-08
PR-14	LOC_Os01g12020	2.00E-07
PR-14	LOC_Os03g59380	3.00E-07

PR-15/16	LOC_Os03g48780	5.00E-98
PR-15/16	LOC_Os03g48760	6.00E-97
PR-15/16	LOC_Os03g48770	1.00E-96
PR-15/16	LOC_Os03g48750	1.00E-93
PR-15/16	LOC_Os02g29020	7.00E-51
PR-15/16	LOC_Os02g29000	1.00E-49
PR-15/16	LOC_Os08g13440	2.00E-49
PR-15/16	LOC_Os05g19670	3.00E-49
PR-15/16	LOC_Os08g08980	3.00E-49
PR-15/16	LOC_Os08g08960	4.00E-49
PR-15/16	LOC_Os08g08970	2.00E-48
PR-15/16	LOC_Os03g44880	3.00E-48
PR-15/16	LOC_Os08g08920	2.00E-47
PR-15/16	LOC_Os03g58980	3.00E-47
PR-15/16	LOC_Os01g18170	3.00E-46
PR-15/16	LOC_Os04g52720	4.00E-46
PR-15/16	LOC_Os01g50900	5.00E-46
PR-15/16	LOC_Os08g09080	7.00E-46
PR-15/16	LOC_Os01g14670	1.00E-45
PR-15/16	LOC_Os08g08990	1.00E-45
PR-15/16	LOC_Os03g59010	2.00E-45
PR-15/16	LOC_Os12g05860	3.00E-45
PR-15/16	LOC_Os12g05840	5.00E-45
PR-15/16	LOC_Os12g05870	1.00E-44
PR-15/16	LOC_Os08g09000	9.00E-44
PR-15/16	LOC_Os08g09060	2.00E-43
PR-15/16	LOC_Os08g09020	5.00E-43
PR-15/16	LOC_Os08g09010	9.00E-43
PR-15/16	LOC_Os08g09040	3.00E-42
PR-15/16	LOC_Os01g72300	1.00E-41
PR-15/16	LOC_Os12g05880	8.00E-37
PR-15/16	LOC_Os02g32980	2.00E-35
PR-15/16	LOC_Os08g35750	2.00E-33
PR-15/16	LOC_Os03g08150	5.00E-32
PR-15/16	LOC_Os08g35760	1.00E-30
PR-15/16	LOC_Os09g39530	4.00E-25
PR-15/16	LOC_Os11g33110	9.00E-25
PR-15/16	LOC_Os09g39520	3.00E-24
PR-15/16	LOC_Os09g39510	5.00E-24
PR-15/16	LOC_Os03g58990	2.00E-20
PR-15/16	LOC_Os03g21790	3.00E-06
