# Supplementary material for: Theobroma cacao L. pathogenesis-related gene tandem array members show diverse expression dynamics in response to pathogen colonization
Source: BMC Genomics. 2016 May 17;17:363. doi: 10.1186/s12864-016-2693-3 (PMC4869279; doi:10.1186/s12864-016-2693-3)
Supplement: Additional file 7: Table S7. — Gene IDs and BLASTp E-values for Populus trichocarpa PR loci. (PDF 4169 kb) [file 12864_2016_2693_MOESM7_ESM.pdf]

Supplemental Table S7 - Gene IDs and BLASTp E-value for *Populus trichocarpa* PR genes

PR Gene Family	Gene ID	E-value
PR-1	Potri.009G083300.1	1.00E-66
PR-1	Potri.009G083100.1	2.00E-66
PR-1	Potri.009G083600.1	4.00E-64
PR-1	Potri.009G083000.1	5.00E-63
PR-1	Potri.T131500.1	8.00E-60
PR-1	Potri.009G082900.1	4.00E-57
PR-1	Potri.001G288600.1	2.00E-56
PR-1	Potri.009G082800.1	9.00E-53
PR-1	Potri.T131400.1	2.00E-52
PR-1	Potri.001G288400.1	2.00E-52
PR-1	Potri.T093500.1	3.00E-48
PR-1	Potri.006G171300.1	5.00E-46
PR-1	Potri.T093600.1	8.00E-43
PR-1	Potri.018G007000.1	9.00E-41
PR-1	Potri.016G082000.1	2.00E-30
PR-1	Potri.006G215600.1	8.00E-30
PR-1	Potri.005G130100.1	1.00E-29
PR-1	Potri.007G033200.1	4.00E-28
PR-1	Potri.009G083400.1	8.00E-14
PR-2	Potri.001G255100.1	1.00E-125
PR-2	Potri.010G142800.1	4.00E-113
PR-2	Potri.T167100.1	1.00E-110
PR-2	Potri.016G057400.1	2.00E-104
PR-2	Potri.009G050300.1	3.00E-103
PR-2	Potri.016G057600.1	8.00E-100
PR-2	Potri.006G046100.1	2.00E-99
PR-2	Potri.006G048100.1	3.00E-98
PR-2	Potri.002G089200.1	2.00E-70
PR-2	Potri.009G050400.1	2.00E-69
PR-2	Potri.005G172000.1	1.00E-68
PR-2	Potri.014G158400.1	1.00E-67
PR-2	Potri.018G068600.1	2.00E-67
PR-2	Potri.006G067500.1	3.00E-66
PR-2	Potri.004G202400.1	1.00E-65
PR-2	Potri.003G032600.1	1.00E-64
PR-2	Potri.011G084900.1	3.00E-64
PR-2	Potri.018G129500.1	4.00E-64
PR-2	Potri.002G224600.1	5.00E-63
PR-2	Potri.004G153800.1	8.00E-63
PR-2	Potri.004G097400.1	8.00E-63
PR-2	Potri.004G132700.1	2.00E-62
PR-2	Potri.001G192200.1	2.00E-62
PR-2	Potri.011G094400.1	4.00E-62
PR-2	Potri.014G182800.1	6.00E-62
PR-2	Potri.014G185100.1	1.00E-61
PR-2	Potri.002G261800.1	2.00E-61
PR-2	Potri.014G184300.1	2.00E-61
PR-2	Potri.009G163700.1	3.00E-61
PR-2	Potri.014G183800.1	4.00E-61
PR-2	Potri.011G152400.1	7.00E-61
PR-2	Potri.014G184100.1	7.00E-61
PR-2	Potri.001G449100.1	1.00E-60
PR-2	Potri.014G182500.1	2.00E-60
PR-2	Potri.012G017800.1	9.00E-60
PR-2	Potri.009G115400.1	1.00E-59
PR-2	Potri.010G108500.1	9.00E-59
PR-2	Potri.004G086400.1	1.00E-58
PR-2	Potri.014G184900.1	7.00E-58
PR-2	Potri.014G182000.1	3.00E-57
PR-2	Potri.010G203800.1	2.00E-56
PR-2	Potri.017G130200.1	2.00E-56
PR-2	Potri.008G055900.1	5.00E-56
PR-2	Potri.002G007300.1	1.00E-55
PR-2	Potri.008G056000.1	1.00E-55
PR-2	Potri.008G133200.1	1.00E-55
PR-2	Potri.001G006500.1	1.00E-55
PR-2	Potri.014G184600.1	2.00E-55
PR-2	Potri.015G010100.1	3.00E-55
PR-2	Potri.004G010500.1	2.00E-54

PR-2	Potri.006G080600.1	4.00E-54
PR-2	Potri.001G240000.1	6.00E-54
PR-3	Potri.004G182000.1	5.00E-95
PR-3	Potri.009G141700.1	5.00E-92
PR-3	Potri.T175200.1	8.00E-80
PR-3	Potri.009G142300.1	2.00E-74
PR-3	Potri.009G142000.1	1.00E-71
PR-3	Potri.T175300.1	8.00E-71
PR-3	Potri.009G142200.1	1.00E-70
PR-3	Potri.009G142100.1	8.00E-70
PR-3	Potri.009G141800.1	3.00E-69
PR-3	Potri.004G182100.1	1.00E-66
PR-3	Potri.014G111800.1	4.00E-65
PR-3	Potri.002G186500.1	2.00E-64
PR-3	Potri.010G141600.1	1.00E-33
PR-3	Potri.019G094000.1	6.00E-33
PR-3	Potri.019G093800.1	7.00E-33
PR-3	Potri.019G094100.1	1.00E-31
PR-3	Potri.013G125000.1	3.00E-31
PR-3	Potri.013G125100.1	3.00E-31
PR-3	Potri.019G093900.1	2.00E-30
PR-3	Potri.014G146600.1	7.00E-30
PR-3	Potri.019G093700.1	7.00E-29
PR-4	Potri.013G041600.1	2.00E-57
PR-4	Potri.013G041700.1	7.00E-56
PR-4	Potri.005G054000.1	9.00E-56
PR-4	Potri.013G041900.1	7.00E-55
PR-5	Potri.012G047800.1	8.00E-89
PR-5	Potri.004G173200.1	8.00E-88
PR-5	Potri.005G112600.1	1.00E-87
PR-5	Potri.015G039200.1	8.00E-87
PR-5	Potri.009G132500.1	9.00E-87
PR-5	Potri.001G221400.1	6.00E-85
PR-5	Potri.014G040700.1	9.00E-84
PR-5	Potri.001G221700.1	7.00E-83
PR-5	Potri.001G222100.1	7.00E-83
PR-5	Potri.001G221100.1	1.00E-82
PR-5	Potri.001G221900.1	1.00E-82
PR-5	Potri.001G221800.1	2.00E-81
PR-5	Potri.001G221500.1	2.00E-81
PR-5	Potri.002G020500.1	1.00E-80
PR-5	Potri.005G240900.1	1.00E-80
PR-5	Potri.001G221200.1	2.00E-80
PR-5	Potri.005G241000.1	5.00E-79
PR-5	Potri.005G112700.1	2.00E-78
PR-5	Potri.002G020400.1	4.00E-78
PR-5	Potri.001G220900.1	2.00E-76
PR-5	Potri.015G000800.1	2.00E-75
PR-5	Potri.005G173900.1	1.00E-73
PR-5	Potri.002G087100.1	6.00E-73
PR-5	Potri.012G004800.1	6.00E-73
PR-5	Potri.006G088100.1	9.00E-73
PR-5	Potri.001G210400.1	3.00E-69
PR-5	Potri.010G200800.1	3.00E-66
PR-5	Potri.003G020100.1	8.00E-66
PR-5	Potri.009G132200.1	2.00E-65
PR-5	Potri.017G075500.1	1.00E-63
PR-5	Potri.004G014700.1	2.00E-62
PR-5	Potri.T091200.1	1.00E-59
PR-5	Potri.011G003900.1	1.00E-56
PR-5	Potri.004G014400.1	3.00E-56
PR-5	Potri.009G028800.1	1.00E-52
PR-5	Potri.001G107600.1	2.00E-52
PR-5	Potri.T091600.1	2.00E-51
PR-5	Potri.T094100.1	4.00E-50
PR-5	Potri.001G102400.1	4.00E-50
PR-5	Potri.001G237600.1	5.00E-49
PR-5	Potri.T091300.1	1.00E-46
PR-5	Potri.001G221300.1	8.00E-42
PR-5	Potri.001G107800.1	4.00E-41
PR-5	Potri.002G133200.1	4.00E-37
PR-5	Potri.004G014200.1	9.00E-32
PR-5	Potri.001G222000.1	1.00E-30
PR-5	Potri.T094200.1	8.00E-24
PR-5	Potri.001G221600.1	1.00E-23
PR-5	Potri.011G004000.1	4.00E-19
PR-5	Potri.001G107700.1	2.00E-17
PR-6	Potri.010G075400.1	2.00E-13
PR-6	Potri.011G110100.1	6.00E-13
PR-6	Potri.010G075600.1	7.00E-13
PR-6	Potri.011G110400.1	7.00E-13
PR-6	Potri.016G078900.1	4.00E-12

PR-6	Potri.010G075800.1	6.00E-12
PR-6	Potri.005G221000.1	3.00E-11
PR-6	Potri.010G075200.1	5.00E-11
PR-6	Potri.016G079100.1	7.00E-11
PR-6	Potri.009G028300.1	1.00E-10
PR-6	Potri.016G079000.1	1.00E-10
PR-6	Potri.006G212200.1	2.00E-10
PR-6	Potri.010G075500.1	4.00E-10
PR-6	Potri.010G075300.1	4.00E-10
PR-6	Potri.010G075700.1	5.00E-10
PR-6	Potri.006G212000.1	6.00E-10
PR-6	Potri.016G078800.1	9.00E-10
PR-6	Potri.002G042300.1	3.00E-07
PR-6	Potri.T083400.1	2.00E-06
PR-6	Potri.006G088700.1	3.00E-06
PR-6	Potri.T082800.1	4.00E-06
PR-6	Potri.006G088500.1	4.00E-06
PR-7	Potri.T004700.1	0
PR-7	Potri.003G118800.1	0
PR-7	Potri.003G118700.1	0
PR-7	Potri.003G118500.1	0
PR-7	Potri.001G113700.1	0
PR-7	Potri.014G074600.1	2.00E-173
PR-7	Potri.009G133400.1	4.00E-166
PR-7	Potri.004G173900.1	7.00E-165
PR-7	Potri.002G256300.1	3.00E-161
PR-7	Potri.003G067000.1	9.00E-157
PR-7	Potri.005G145300.1	3.00E-156
PR-7	Potri.001G167300.1	3.00E-153
PR-7	Potri.014G018900.1	6.00E-148
PR-7	Potri.002G120400.1	1.00E-147
PR-7	Potri.014G171600.1	2.00E-147
PR-7	Potri.011G165900.1	3.00E-146
PR-7	Potri.012G131500.1	1.00E-144
PR-7	Potri.001G163600.1	2.00E-143
PR-7	Potri.001G469000.1	3.00E-141
PR-7	Potri.001G468800.1	7.00E-139
PR-7	Potri.015G133800.1	2.00E-136
PR-7	Potri.007G102100.1	1.00E-135
PR-7	Potri.005G067200.1	2.00E-133
PR-7	Potri.011G050000.1	1.00E-132
PR-7	Potri.007G102200.1	3.00E-132
PR-7	Potri.007G102300.1	9.00E-132
PR-7	Potri.001G455800.1	1.00E-131
PR-7	Potri.001G450600.1	6.00E-131
PR-7	Potri.010G196700.1	4.00E-130
PR-7	Potri.001G468900.1	1.00E-129
PR-7	Potri.007G045100.1	7.00E-129
PR-7	Potri.011G146300.1	5.00E-127
PR-7	Potri.011G076700.1	6.00E-127
PR-7	Potri.011G050100.1	1.00E-126
PR-7	Potri.011G150900.1	5.00E-126
PR-7	Potri.010G196800.1	3.00E-125
PR-7	Potri.006G001600.1	3.00E-125
PR-7	Potri.004G161400.1	5.00E-125
PR-7	Potri.009G100500.1	1.00E-124
PR-7	Potri.006G114500.1	1.00E-124
PR-7	Potri.011G151200.1	2.00E-122
PR-7	Potri.002G124500.1	2.00E-122
PR-7	Potri.011G050300.1	3.00E-122
PR-7	Potri.005G243000.1	2.00E-120
PR-7	Potri.014G026600.1	2.00E-120
PR-7	Potri.011G050200.1	3.00E-120
PR-7	Potri.002G018600.1	2.00E-119
PR-7	Potri.014G026700.1	3.00E-119
PR-7	Potri.009G038000.1	1.00E-117
PR-7	Potri.010G196600.1	2.00E-116
PR-7	Potri.012G133200.1	3.00E-116
PR-7	Potri.010G196900.1	1.00E-115
PR-7	Potri.014G026500.1	3.00E-115
PR-7	Potri.003G120100.1	2.00E-114
PR-7	Potri.014G074500.1	2.00E-113
PR-7	Potri.018G094400.1	2.00E-111
PR-7	Potri.003G189200.1	7.00E-111
PR-7	Potri.009G037900.1	1.00E-105
PR-7	Potri.002G151900.1	1.00E-104
PR-7	Potri.006G141200.1	3.00E-100
PR-7	Potri.009G144500.1	3.00E-99
PR-7	Potri.001G002200.1	1.00E-85
PR-7	Potri.006G076200.1	5.00E-78
PR-7	Potri.004G184600.1	1.00E-77
PR-7	Potri.001G440300.1	6.00E-77

PR-7	Potri.001G151100.1	2.00E-76
PR-7	Potri.011G155400.1	6.00E-75
PR-7	Potri.014G193200.1	1.00E-74
PR-7	Potri.018G143400.1	4.00E-74
PR-7	Potri.002G152000.1	4.00E-65
PR-8	Potri.015G024100.1	1.00E-131
PR-8	Potri.015G024000.1	3.00E-131
PR-8	Potri.015G023900.1	2.00E-128
PR-8	Potri.002G165700.1	5.00E-110
PR-8	Potri.014G091700.1	1.00E-109
PR-8	Potri.015G024200.1	1.00E-105
PR-8	Potri.002G242000.1	3.00E-105
PR-8	Potri.012G033900.1	7.00E-103
PR-8	Potri.014G091600.1	8.00E-103
PR-8	Potri.014G092800.1	2.00E-88
PR-8	Potri.014G092900.1	1.00E-82
PR-8	Potri.014G093000.1	2.00E-22
PR-9	Potri.001G011500.1	9.00E-142
PR-9	Potri.003G214800.1	4.00E-139
PR-9	Potri.003G214700.1	5.00E-139
PR-9	Potri.016G058200.1	4.00E-130
PR-9	Potri.001G011200.1	1.00E-122
PR-9	Potri.001G011300.1	7.00E-122
PR-9	Potri.003G214900.1	1.00E-121
PR-9	Potri.001G013000.1	1.00E-121
PR-9	Potri.001G011000.1	1.00E-121
PR-9	Potri.003G214500.1	2.00E-107
PR-9	Potri.003G215000.1	8.00E-102
PR-9	Potri.007G019300.1	6.00E-100
PR-9	Potri.005G118700.1	7.00E-99
PR-9	Potri.013G083600.1	3.00E-97
PR-9	Potri.013G154400.1	1.00E-96
PR-9	Potri.014G143200.1	2.00E-96
PR-9	Potri.T160000.1	2.00E-96
PR-9	Potri.008G022700.1	4.00E-96
PR-9	Potri.008G022600.1	2.00E-95
PR-9	Potri.T160100.1	4.00E-95
PR-9	Potri.T163200.1	7.00E-95
PR-9	Potri.001G458900.1	4.00E-92
PR-9	Potri.016G132700.1	1.00E-91
PR-9	Potri.010G236900.1	6.00E-91

PR-9	Potri.001G458700.1	1.00E-90
PR-9	Potri.T163000.1	3.00E-90
PR-9	Potri.005G135300.1	3.00E-90
PR-9	Potri.002G031200.1	4.00E-90
PR-9	Potri.T163100.1	8.00E-89
PR-9	Potri.013G156800.1	2.00E-88
PR-9	Potri.008G103200.1	1.00E-87
PR-9	Potri.013G156400.1	1.00E-85
PR-9	Potri.011G027300.1	3.00E-85
PR-9	Potri.004G144600.1	1.00E-84
PR-9	Potri.004G023200.1	1.00E-84
PR-9	Potri.013G156500.1	3.00E-84
PR-9	Potri.009G106400.1	4.00E-84
PR-9	Potri.010G134500.1	1.00E-83
PR-9	Potri.T099300.1	1.00E-83
PR-9	Potri.016G132900.1	2.00E-83
PR-9	Potri.019G063200.1	3.00E-83
PR-9	Potri.008G110600.1	4.00E-83
PR-9	Potri.006G069600.1	8.00E-83
PR-9	Potri.004G023100.1	2.00E-82
PR-9	Potri.016G132800.1	2.00E-82
PR-9	Potri.018G131600.1	4.00E-81
PR-9	Potri.001G145800.1	6.00E-81
PR-9	Potri.006G107000.1	2.00E-80
PR-9	Potri.005G072800.1	2.00E-80
PR-9	Potri.017G038100.1	4.00E-80
PR-9	Potri.005G108900.1	1.00E-79
PR-9	Potri.007G122100.1	9.00E-79
PR-9	Potri.002G065300.1	1.00E-78
PR-9	Potri.007G096200.1	2.00E-78
PR-9	Potri.017G037900.1	3.00E-77
PR-9	Potri.005G195600.1	4.00E-76
PR-9	Potri.012G042800.1	4.00E-76
PR-9	Potri.016G058800.1	6.00E-76
PR-9	Potri.016G125000.1	2.00E-73
PR-9	Potri.010G175100.1	4.00E-73
PR-9	Potri.005G195700.1	2.00E-72
PR-9	Potri.001G182400.1	2.00E-72
PR-9	Potri.004G134800.1	7.00E-72
PR-9	Potri.003G156100.1	8.00E-71
PR-9	Potri.002G018000.1	9.00E-71

PR-9	Potri.007G122200.1	1.00E-70
PR-9	Potri.017G064100.1	4.00E-70
PR-9	Potri.007G122300.1	7.00E-69
PR-9	Potri.018G089900.1	2.00E-68
PR-9	Potri.007G074700.1	7.00E-68
PR-9	Potri.010G036100.1	1.00E-67
PR-9	Potri.T045500.1	3.00E-67
PR-9	Potri.015G003500.1	3.00E-67
PR-9	Potri.015G003600.1	3.00E-66
PR-9	Potri.007G053400.1	2.00E-65
PR-9	Potri.004G052100.1	4.00E-65
PR-9	Potri.011G062300.1	5.00E-65
PR-9	Potri.001G329200.1	6.00E-65
PR-9	Potri.001G351000.1	1.00E-64
PR-9	Potri.007G132800.1	1.00E-63
PR-9	Potri.015G110200.1	1.00E-61
PR-9	Potri.T089600.1	1.00E-61
PR-9	Potri.018G136900.1	1.00E-60
PR-9	Potri.018G015500.1	2.00E-60
PR-9	Potri.012G076500.1	3.00E-60
PR-9	Potri.013G066800.1	5.00E-60
PR-9	Potri.006G267400.1	2.00E-59
PR-9	Potri.007G067200.1	3.00E-58
PR-9	Potri.001G218600.1	1.00E-55
PR-9	Potri.001G218500.1	2.00E-55
PR-9	Potri.006G129900.1	8.00E-54
PR-10	Potri.010G000600.1	6.00E-45
PR-10	Potri.011G026000.1	9.00E-44
PR-10	Potri.008G212100.1	3.00E-43
PR-10	Potri.011G025900.1	9.00E-43
PR-10	Potri.011G026100.1	1.00E-42
PR-10	Potri.011G026200.1	5.00E-42
PR-10	Potri.008G212500.1	1.00E-41
PR-10	Potri.014G152800.1	2.00E-41
PR-10	Potri.008G212400.1	8.00E-41
PR-10	Potri.004G021100.1	9.00E-41
PR-10	Potri.010G000400.1	1.00E-40
PR-10	Potri.T111200.1	2.00E-40
PR-10	Potri.010G000200.1	2.00E-39
PR-10	Potri.016G046500.1	3.00E-39
PR-10	Potri.008G213100.1	7.00E-39

PR-10	Potri.008G212700.1	8.00E-38
PR-10	Potri.008G212600.1	4.00E-35
PR-10	Potri.010G000500.1	4.00E-32
PR-10	Potri.008G212300.1	2.00E-29
PR-10	Potri.008G212800.1	2.00E-26
PR-10	Potri.004G032900.1	4.00E-20
PR-10	Potri.004G033000.1	1.00E-19
PR-11	Potri.006G188300.1	1.00E-146
PR-11	Potri.006G188400.1	4.00E-138
PR-11	Potri.018G111600.1	1.00E-98
PR-11	Potri.018G111900.1	8.00E-95
PR-11	Potri.018G112000.1	3.00E-90
PR-11	Potri.018G112100.1	1.00E-88
PR-11	Potri.018G111700.1	9.00E-86
PR-11	Potri.006G261800.1	1.00E-85
PR-11	Potri.018G111800.1	1.00E-58
PR-14	Potri.001G232900.1	1.00E-21
PR-14	Potri.001G232700.1	4.00E-21
PR-14	Potri.004G086600.1	8.00E-20
PR-14	Potri.004G086500.1	8.00E-19
PR-14	Potri.009G025200.1	6.00E-17
PR-14	Potri.T132400.1	6.00E-17
PR-14	Potri.016G136000.1	6.00E-17
PR-14	Potri.016G135400.1	9.00E-17
PR-14	Potri.016G135800.1	5.00E-16
PR-14	Potri.014G046500.1	5.00E-15
PR-14	Potri.016G135700.1	2.00E-14
PR-14	Potri.006G108100.1	6.00E-14
PR-14	Potri.016G135500.1	2.00E-13
PR-14	Potri.014G098000.1	8.00E-11
PR-14	Potri.011G021900.1	6.00E-09
PR-16	Potri.015G068200.1	8.00E-65
PR-16	Potri.005G001300.1	1.00E-59
PR-16	Potri.011G163300.1	9.00E-59
PR-16	Potri.013G052300.1	2.00E-58
PR-16	Potri.T151800.1	3.00E-58
PR-16	Potri.013G052100.1	6.00E-58
PR-16	Potri.013G052000.1	7.00E-58
PR-16	Potri.013G051700.1	2.00E-57
PR-16	Potri.011G163800.1	3.00E-57
PR-16	Potri.013G051600.1	4.00E-57

PR-16	Potri.011G163200.1	8.00E-57
PR-16	Potri.001G465100.1	9.00E-57
PR-16	Potri.011G162200.1	1.00E-56
PR-16	Potri.014G110400.1	2.00E-55
PR-16	Potri.013G064100.1	1.00E-54
PR-16	Potri.013G000500.1	1.00E-54
PR-16	Potri.002G184900.1	1.00E-54
PR-16	Potri.013G063000.1	2.00E-54
PR-16	Potri.013G063800.1	2.00E-54
PR-16	Potri.013G063600.1	2.00E-54
PR-16	Potri.013G063700.1	2.00E-54
PR-16	Potri.013G063900.1	2.00E-54
PR-16	Potri.013G063500.1	2.00E-54
PR-16	Potri.013G064000.1	2.00E-54
PR-16	Potri.013G063200.1	2.00E-54
PR-16	Potri.013G063100.1	6.00E-54
PR-16	Potri.T151900.1	9.00E-54
PR-16	Potri.019G026400.1	3.00E-53
PR-16	Potri.013G063300.1	6.00E-53
PR-16	Potri.019G026700.1	1.00E-52
PR-16	Potri.019G026000.1	1.00E-52
PR-16	Potri.019G025800.1	1.00E-52
PR-16	Potri.019G025900.1	1.00E-52
PR-16	Potri.T056300.1	2.00E-52
PR-16	Potri.019G026200.1	3.00E-52
PR-16	Potri.019G026500.1	4.00E-52
PR-16	Potri.013G051900.1	3.00E-51
PR-16	Potri.001G464000.1	4.00E-51
PR-16	Potri.001G464100.1	8.00E-51
PR-16	Potri.011G162600.1	1.00E-49
PR-16	Potri.013G051800.1	1.00E-48
PR-16	Potri.009G140400.1	2.00E-48
PR-16	Potri.004G180100.1	2.00E-48
PR-16	Potri.004G179800.1	2.00E-48
PR-16	Potri.004G179900.1	2.00E-48
PR-16	Potri.013G063400.1	1.00E-47

PR-16	Potri.004G180000.1	2.00E-46
PR-16	Potri.004G180200.1	2.00E-46
PR-16	Potri.001G464500.1	2.00E-46
PR-16	Potri.008G020800.1	5.00E-44
PR-16	Potri.010G038200.1	9.00E-40
PR-16	Potri.013G141900.1	4.00E-35
PR-16	Potri.003G065300.1	4.00E-35
PR-16	Potri.012G111500.1	1.00E-34
PR-16	Potri.010G238100.1	2.00E-34
PR-16	Potri.010G238200.1	2.00E-34
PR-16	Potri.001G169000.1	2.00E-34
PR-16	Potri.006G142600.1	3.00E-33
PR-16	Potri.015G109600.1	7.00E-33
PR-16	Potri.019G026100.1	9.00E-31
PR-16	Potri.T055000.1	4.00E-29
PR-16	Potri.019G029300.1	2.00E-28
PR-16	Potri.008G016700.1	8.00E-28
PR-16	Potri.004G194600.1	1.00E-26
PR-16	Potri.010G240700.1	4.00E-26
PR-16	Potri.008G084300.1	6.00E-26
PR-16	Potri.010G240500.1	2.00E-24
PR-16	Potri.009G157100.1	2.00E-24
PR-16	Potri.010G240600.1	5.00E-24
PR-16	Potri.019G026800.1	2.00E-23
PR-16	Potri.008G016500.1	6.00E-14
PR-16	Potri.013G116500.1	2.00E-12
PR-16	Potri.008G016600.1	7.00E-07
PR-16	Potri.001G464600.1	1.00E-05
PR-17	Potri.009G094600.1	3.00E-111
PR-17	Potri.001G299500.1	3.00E-111
PR-17	Potri.001G299400.1	3.00E-107
PR-17	Potri.001G299600.1	1.00E-103
PR-17	Potri.009G094500.1	2.00E-93
PR-17	Potri.009G094700.1	7.00E-87
PR-17	Potri.005G202300.1	8.00E-09
