# Supplementary material for: Theobroma cacao L. pathogenesis-related gene tandem array members show diverse expression dynamics in response to pathogen colonization
Source: BMC Genomics. 2016 May 17;17:363. doi: 10.1186/s12864-016-2693-3 (PMC4869279; doi:10.1186/s12864-016-2693-3)
Supplement: Additional file 8: Table S8. — Gene IDs and BLASTp E-values for Vitis vinifera PR loci. (PDF 4169 kb) [file 12864_2016_2693_MOESM8_ESM.pdf]

**Supplemental Table S8 - Gene IDs and BLASTp E-value for *Vitis vinifera* PR genes**		
**PR Gene Class**	**Gene ID**	**E-value**
PR-1	GSVIVT01037000001	4.00E-53
PR-1	GSVIVT01037005001	1.00E-51
PR-1	GSVIVT01036997001	1.00E-49
PR-1	GSVIVT01037008001	1.00E-49
PR-1	GSVIVT01038540001	2.00E-49
PR-1	GSVIVT01037015001	1.00E-43
PR-1	GSVIVT01036993001	1.00E-40
PR-1	GSVIVT01037011001	1.00E-40
PR-1	GSVIVT01029124001	6.00E-40
PR-1	GSVIVT01025577001	8.00E-40
PR-1	GSVIVT01003762001	3.00E-28
PR-1	GSVIVT01037014001	6.00E-19
PR-1	GSVIVT01003765001	2.00E-06
PR-2	GSVIVT01031542001	1.00E-77
PR-2	GSVIVT01033545001	5.00E-76
PR-2	GSVIVT01033542001	6.00E-75
PR-2	GSVIVT01013131001	6.00E-67
PR-2	GSVIVT01015282001	2.00E-61
PR-2	GSVIVT01033536001	4.00E-60
PR-2	GSVIVT01017331001	1.00E-55
PR-2	GSVIVT01037711001	4.00E-54
PR-2	GSVIVT01031959001	7.00E-54
PR-2	GSVIVT01021499001	8.00E-54
PR-2	GSVIVT01035013001	5.00E-53
PR-2	GSVIVT01033538001	7.00E-53
PR-2	GSVIVT01038583001	1.00E-52
PR-2	GSVIVT01011682001	3.00E-52
PR-2	GSVIVT01030716001	9.00E-52
PR-2	GSVIVT01036124001	1.00E-51
PR-2	GSVIVT01009700001	1.00E-50
PR-2	GSVIVT01024956001	5.00E-50
PR-2	GSVIVT01012711001	3.00E-48
PR-2	GSVIVT01022253001	4.00E-48
PR-2	GSVIVT01013401001	1.00E-46
PR-2	GSVIVT01031698001	1.00E-46
PR-2	GSVIVT01034245001	3.00E-46
PR-2	GSVIVT01033384001	4.00E-44
PR-2	GSVIVT01015563001	1.00E-42
PR-2	GSVIVT01031544001	2.00E-41
PR-2	GSVIVT01030073001	3.00E-40
PR-2	GSVIVT01016553001	6.00E-40
PR-2	GSVIVT01019678001	4.00E-39
PR-2	GSVIVT01016379001	9.00E-39
PR-2	GSVIVT01033540001	2.00E-36
PR-2	GSVIVT01015435001	7.00E-36
PR-2	GSVIVT01014995001	4.00E-35
PR-2	GSVIVT01031543001	3.00E-30
PR-2	GSVIVT01030503001	6.00E-30
PR-2	GSVIVT01002584001	8.00E-30
PR-2	GSVIVT01033543001	6.00E-27
PR-2	GSVIVT01031619001	3.00E-25
PR-2	GSVIVT01015616001	1.00E-11
PR-2	GSVIVT01025431001	3.00E-11
PR-2	GSVIVT01000128001	3.00E-08
PR-2	GSVIVT01017294001	3.00E-06
PR-3	GSVIVT01007190001	1.00E-69
PR-3	GSVIVT01038126001	8.00E-30
PR-3	GSVIVT01038114001	1.00E-29
PR-3	GSVIVT01028243001	3.00E-28
PR-3	GSVIVT01038125001	8.00E-28
PR-3	GSVIVT01038113001	1.00E-27
PR-3	GSVIVT01038117001	1.00E-27
PR-3	GSVIVT01038111001	2.00E-27
PR-3	GSVIVT01038116001	4.00E-27
PR-3	GSVIVT01038120001	1.00E-25
PR-3	GSVIVT01038108001	6.00E-25

PR-3	GSVIVT01035029001	2.00E-23
PR-3	GSVIVT01031685001	1.00E-22
PR-4	GSVIVT01036278001	1.00E-48
PR-4	GSVIVT01036279001	7.00E-48
PR-4	GSVIVT01036281001	7.00E-16
PR-5	GSVIVT01008423001	7.00E-73
PR-5	GSVIVT01032051001	4.00E-70
PR-5	GSVIVT01018767001	1.00E-69
PR-5	GSVIVT01009930001	1.00E-67
PR-5	GSVIVT01024050001	2.00E-67
PR-5	GSVIVT01027712001	1.00E-65
PR-5	GSVIVT01018769001	2.00E-65
PR-5	GSVIVT01009928001	2.00E-64
PR-5	GSVIVT01038679001	6.00E-64
PR-5	GSVIVT01024052001	1.00E-61
PR-5	GSVIVT01033694001	4.00E-60
PR-5	GSVIVT01016504001	5.00E-58
PR-5	GSVIVT01034131001	2.00E-56
PR-5	GSVIVT01008918001	4.00E-55
PR-5	GSVIVT01022993001	3.00E-54
PR-5	GSVIVT01009646001	2.00E-38
PR-5	GSVIVT01032560001	1.00E-34
PR-5	GSVIVT01019840001	4.00E-27
PR-5	GSVIVT01019849001	7.00E-21
PR-5	GSVIVT01019835001	4.00E-14
PR-5	GSVIVT01019848001	8.00E-10
PR-5	GSVIVT01019838001	2.00E-06
PR-5	GSVIVT01019836001	7.00E-06
PR-6	GSVIVT01018137001	3.00E-13
PR-6	GSVIVT01018139001	8.00E-13
PR-6	GSVIVT01018142001	2.00E-10
PR-6	GSVIVT01018141001	4.00E-09
PR-6	GSVIVT01032743001	1.00E-08
PR-7	GSVIVT01028435001	7.00E-138
PR-7	GSVIVT01024042001	2.00E-134
PR-7	GSVIVT01028051001	2.00E-126
PR-7	GSVIVT01006970001	3.00E-122
PR-7	GSVIVT01030138001	4.00E-122
PR-7	GSVIVT01014788001	2.00E-119
PR-7	GSVIVT01019901001	3.00E-119
PR-7	GSVIVT01015069001	4.00E-118
PR-7	GSVIVT01006972001	2.00E-117
PR-7	GSVIVT01016448001	1.00E-114
PR-7	GSVIVT01019687001	2.00E-114
PR-7	GSVIVT01009471001	3.00E-114
PR-7	GSVIVT01006968001	4.00E-114
PR-7	GSVIVT01009968001	3.00E-112
PR-7	GSVIVT01016449001	5.00E-112
PR-7	GSVIVT01026420001	9.00E-112
PR-7	GSVIVT01016446001	1.00E-111
PR-7	GSVIVT01016452001	1.00E-111
PR-7	GSVIVT01016456001	7.00E-111
PR-7	GSVIVT01016447001	4.00E-110
PR-7	GSVIVT01016439001	3.00E-109
PR-7	GSVIVT01027586001	4.00E-109
PR-7	GSVIVT01019877001	7.00E-109
PR-7	GSVIVT01019919001	3.00E-108
PR-7	GSVIVT01016455001	4.00E-107
PR-7	GSVIVT01027368001	1.00E-106
PR-7	GSVIVT01033810001	5.00E-106
PR-7	GSVIVT01016451001	7.00E-106
PR-7	GSVIVT01027583001	3.00E-105
PR-7	GSVIVT01038642001	7.00E-105
PR-7	GSVIVT01038641001	1.00E-104
PR-7	GSVIVT01016443001	5.00E-104
PR-7	GSVIVT01010670001	1.00E-103
PR-7	GSVIVT01024856001	3.00E-103
PR-7	GSVIVT01038620001	2.00E-102
PR-7	GSVIVT01010668001	3.00E-102
PR-7	GSVIVT01036167001	9.00E-102
PR-7	GSVIVT01018437001	1.00E-101
PR-7	GSVIVT01016682001	1.00E-100
PR-7	GSVIVT01021320001	1.00E-100
PR-7	GSVIVT01018442001	2.00E-100
PR-7	GSVIVT01024195001	4.00E-100
PR-7	GSVIVT01019686001	6.00E-99
PR-7	GSVIVT01024859001	2.00E-98
PR-7	GSVIVT01037483001	3.00E-98
PR-7	GSVIVT01024948001	3.00E-96
PR-7	GSVIVT01024857001	6.00E-96
PR-7	GSVIVT01018438001	4.00E-93
PR-7	GSVIVT01016735001	6.00E-93

PR-7	GSVIVT01025493001	6.00E-93
PR-7	GSVIVT01016445001	4.00E-92
PR-7	GSVIVT01037485001	1.00E-90
PR-7	GSVIVT01002783001	3.00E-89
PR-7	GSVIVT01016442001	2.00E-88
PR-7	GSVIVT01031724001	1.00E-87
PR-7	GSVIVT01031725001	1.00E-83
PR-7	GSVIVT01006973001	1.00E-81
PR-7	GSVIVT01019899001	3.00E-81
PR-7	GSVIVT01000156001	6.00E-81
PR-7	GSVIVT01031723001	2.00E-76
PR-7	GSVIVT01010871001	3.00E-73
PR-7	GSVIVT01037948001	3.00E-70
PR-7	GSVIVT01009472001	4.00E-70
PR-7	GSVIVT01029534001	1.00E-67
PR-7	GSVIVT01004808001	9.00E-65
PR-7	GSVIVT01006971001	1.00E-50
PR-7	GSVIVT01024858001	1.00E-26
PR-8	GSVIVT01027027001	1.00E-85
PR-8	GSVIVT01032411001	5.00E-81
PR-8	GSVIVT01027012001	9.00E-77
PR-8	GSVIVT01028750001	8.00E-73
PR-8	GSVIVT01028752001	7.00E-71
PR-8	GSVIVT01027026001	1.00E-67
PR-8	GSVIVT01027014001	2.00E-64
PR-8	GSVIVT01027013001	5.00E-63
PR-8	GSVIVT01027022001	3.00E-49
PR-8	GSVIVT01027016001	3.00E-35
PR-8	GSVIVT01028753001	6.00E-31
PR-9	GSVIVT01025373001	9.00E-114
PR-9	GSVIVT01033484001	4.00E-105
PR-9	GSVIVT01020737001	5.00E-79
PR-9	GSVIVT01024596001	1.00E-75
PR-9	GSVIVT01010271001	3.00E-75
PR-9	GSVIVT01031801001	5.00E-74
PR-9	GSVIVT01030221001	8.00E-74
PR-9	GSVIVT01010269001	4.00E-72
PR-9	GSVIVT01024600001	5.00E-71
PR-9	GSVIVT01009106001	2.00E-70
PR-9	GSVIVT01013238001	4.00E-70
PR-9	GSVIVT01015533001	6.00E-70

PR-9	GSVIVT01010168001	1.00E-69
PR-9	GSVIVT01010272001	5.00E-67
PR-9	GSVIVT01010267001	2.00E-66
PR-9	GSVIVT01003417001	3.00E-66
PR-9	GSVIVT01010270001	3.00E-66
PR-9	GSVIVT01034967001	3.00E-66
PR-9	GSVIVT01015537001	4.00E-66
PR-9	GSVIVT01030219001	2.00E-65
PR-9	GSVIVT01009107001	7.00E-65
PR-9	GSVIVT01010268001	3.00E-64
PR-9	GSVIVT01010664001	3.00E-64
PR-9	GSVIVT01018436001	3.00E-64
PR-9	GSVIVT01011017001	1.00E-63
PR-9	GSVIVT01026134001	1.00E-63
PR-9	GSVIVT01000248001	2.00E-61
PR-9	GSVIVT01017830001	7.00E-61
PR-9	GSVIVT01008763001	1.00E-60
PR-9	GSVIVT01031311001	1.00E-60
PR-9	GSVIVT01021152001	2.00E-60
PR-9	GSVIVT01030615001	5.00E-60
PR-9	GSVIVT01030616001	8.00E-60
PR-9	GSVIVT01010080001	1.00E-59
PR-9	GSVIVT01031312001	1.00E-59
PR-9	GSVIVT01004097001	3.00E-59
PR-9	GSVIVT01029748001	7.00E-58
PR-9	GSVIVT01009109001	1.00E-57
PR-9	GSVIVT01004088001	7.00E-57
PR-9	GSVIVT01005386001	8.00E-57
PR-9	GSVIVT01029241001	2.00E-56
PR-9	GSVIVT01007448001	4.00E-55
PR-9	GSVIVT01017084001	4.00E-55
PR-9	GSVIVT01028197001	3.00E-54
PR-9	GSVIVT01010266001	4.00E-54
PR-9	GSVIVT01018865001	3.00E-53
PR-9	GSVIVT01010156001	2.00E-52
PR-9	GSVIVT01009108001	5.00E-52
PR-9	GSVIVT01012624001	1.00E-51
PR-9	GSVIVT01038659001	2.00E-51
PR-9	GSVIVT01029771001	2.00E-50
PR-9	GSVIVT01036100001	8.00E-49
PR-9	GSVIVT01025365001	1.00E-48

PR-9	GSVIVT01033081001	4.00E-48
PR-9	GSVIVT01034984001	1.00E-46
PR-9	GSVIVT01025650001	9.00E-45
PR-9	GSVIVT01032715001	1.00E-44
PR-9	GSVIVT01024599001	4.00E-43
PR-9	GSVIVT01025374001	4.00E-41
PR-9	GSVIVT01009777001	6.00E-39
PR-9	GSVIVT01029774001	3.00E-36
PR-9	GSVIVT01020738001	9.00E-36
PR-9	GSVIVT01029776001	9.00E-36
PR-9	GSVIVT01036009001	1.00E-35
PR-9	GSVIVT01000144001	3.00E-35
PR-9	GSVIVT01029773001	3.00E-35
PR-9	GSVIVT01029775001	3.00E-35
PR-9	GSVIVT01010261001	1.00E-32
PR-9	GSVIVT01007225001	5.00E-32
PR-9	GSVIVT01034574001	2.00E-31
PR-9	GSVIVT01017829001	4.00E-28
PR-9	GSVIVT01012727001	1.00E-26
PR-9	GSVIVT01029778001	4.00E-21
PR-9	GSVIVT01015536001	9.00E-17
PR-9	GSVIVT01035858001	5.00E-14
PR-9	GSVIVT01033080001	4.00E-12
PR-9	GSVIVT01008846001	4.00E-10
PR-9	GSVIVT01025104001	4.00E-10
PR-9	GSVIVT01024035001	4.00E-09
PR-10	GSVIVT01035071001	1.00E-44
PR-10	GSVIVT01035072001	2.00E-42
PR-10	GSVIVT01035062001	8.00E-35
PR-10	GSVIVT01035055001	3.00E-34
PR-10	GSVIVT01035074001	3.00E-34
PR-10	GSVIVT01035076001	1.00E-33
PR-10	GSVIVT01035061001	2.00E-33
PR-10	GSVIVT01035060001	5.00E-33
PR-10	GSVIVT01035054001	6.00E-33
PR-10	GSVIVT01035059001	2.00E-32
PR-10	GSVIVT01035066001	3.00E-28
PR-10	GSVIVT01035069001	3.00E-28
PR-10	GSVIVT01035075001	9.00E-23
PR-10	GSVIVT01028061001	7.00E-22
PR-10	GSVIVT01028060001	4.00E-14

PR-10	GSVIVT01035068001	2.00E-12
PR-11	GSVIVT01003718001	1.00E-119
PR-11	GSVIVT01001068001	3.00E-118
PR-11	GSVIVT01001074001	1.00E-101
PR-11	GSVIVT01001059001	3.00E-81
PR-11	GSVIVT01001064001	5.00E-81
PR-11	GSVIVT01007371001	3.00E-78
PR-11	GSVIVT01001058001	3.00E-76
PR-11	GSVIVT01001061001	1.00E-42
PR-11	GSVIVT01007373001	6.00E-23
PR-14	GSVIVT01030190001	9.00E-15
PR-14	GSVIVT01024563001	3.00E-14
PR-14	GSVIVT01006001001	1.00E-10
PR-14	GSVIVT01032682001	9.00E-10
PR-14	GSVIVT01021772001	4.00E-09
PR-14	GSVIVT01027088001	7.00E-06
PR-16	GSVIVT01030965001	5.00E-68
PR-16	GSVIVT01030968001	1.00E-67
PR-16	GSVIVT01030967001	3.00E-67
PR-16	GSVIVT01031079001	3.00E-67
PR-16	GSVIVT01000101001	4.00E-67
PR-16	GSVIVT01000102001	1.00E-66
PR-16	GSVIVT01000095001	2.00E-66
PR-16	GSVIVT01000098001	3.00E-66
PR-16	GSVIVT01000103001	2.00E-65
PR-16	GSVIVT01000097001	5.00E-65
PR-16	GSVIVT01000100001	5.00E-65
PR-16	GSVIVT01021705001	9.00E-65
PR-16	GSVIVT01013822001	1.00E-63
PR-16	GSVIVT01031080001	1.00E-63
PR-16	GSVIVT01031082001	3.00E-63
PR-16	GSVIVT01031085001	3.00E-63
PR-16	GSVIVT01000067001	8.00E-62
PR-16	GSVIVT01000070001	9.00E-62
PR-16	GSVIVT01000071001	9.00E-62
PR-16	GSVIVT01000063001	2.00E-61
PR-16	GSVIVT01021699001	2.00E-61
PR-16	GSVIVT01000068001	1.00E-60
PR-16	GSVIVT01000058001	1.00E-58
PR-16	GSVIVT01000060001	5.00E-57
PR-16	GSVIVT01000066001	2.00E-55

PR-16	GSVIVT01021700001	5.00E-55
PR-16	GSVIVT01000062001	7.00E-55
PR-16	GSVIVT01031351001	3.00E-46
PR-16	GSVIVT01028222001	4.00E-44
PR-16	GSVIVT01028220001	1.00E-40
PR-16	GSVIVT01020142001	4.00E-35
PR-16	GSVIVT01008094001	3.00E-34
PR-16	GSVIVT01000069001	8.00E-31
PR-16	GSVIVT01016761001	1.00E-25
PR-16	GSVIVT01036097001	1.00E-25

PR-16	GSVIVT01038545001	1.00E-25
PR-16	GSVIVT01000065001	6.00E-25
PR-16	GSVIVT01028588001	3.00E-23
PR-16	GSVIVT01028594001	3.00E-23
PR-16	GSVIVT01036098001	1.00E-22
PR-16	GSVIVT01025139001	7.00E-06
PR-17	GSVIVT01037910001	2.00E-89
PR-17	GSVIVT01037911001	8.00E-49
PR-17	GSVIVT01020876001	2.00E-09
