# Supplementary material for: Theobroma cacao L. pathogenesis-related gene tandem array members show diverse expression dynamics in response to pathogen colonization
Source: BMC Genomics. 2016 May 17;17:363. doi: 10.1186/s12864-016-2693-3 (PMC4869279; doi:10.1186/s12864-016-2693-3)
Supplement: Additional file 9: Table S9. — Percentage of PR genes in tandem arrays in the six analyzed plant species. (PDF 4169 kb) [file 12864_2016_2693_MOESM9_ESM.pdf]

Supplemental Table S9 - Percentage of PR family members in tandem arrays.						
	T. cacao	*B. distachyon*	*O. Sativa*	*A. thaliana*	*V. vinifera*	*P. trichocarpa*
**PR-1**	57.1	73.3	92.3	54.5	61.5	47.4
**PR-2**	11.6	23.7	28.8	14.0	21.4	28.8
**PR-3**	54.5	46.2	56.3	42.9	69.2	71.4
**PR-4**	75.0	100.0	100.0	0**	100.0	75.0
**PR-5**	50.0	57.6	51.4	29.2	52.2	56.0
**PR-6**	87.5	60.0	0.0	80.0	100.0	77.3
**PR-7**	50.0	28.3	39.7	56.0	53.7	42.9
**PR-8**	71.4	71.4	69.0	0**	90.9	75.0
**PR-9**	40.7	67.8	68.2	35.1	50.6	37.4
**PR-10**	82.6	66.7	50.0	0**	100.0	81.8
**PR-11**	81.8	0**	0.0	100.0	66.7	88.9
**PR-12**	66.7	NA*	NA*	57.1	NA*	NA*
**PR-13**	NA*	100.0	90.9	0*	NA*	NA*
**PR-14**	62.5	44.4	52.9	53.8	0.0	60.0
**PR-15/16**	52.6	56.8***	70.7***	66.7	80.5	70.3
**PR-17**	100.0	0**	NA*	100.0	66.7	85.7
* No members of this family were identified in this species.						
** There is only one member of the family for this species, so no tandem arrays can exist						
*** Monocots have both PR-15s and PR-16s, which are distantly phylogenetically related. This percentage groups the two families together.						
