# Supplementary material for: Theobroma cacao L. pathogenesis-related gene tandem array members show diverse expression dynamics in response to pathogen colonization
Source: BMC Genomics. 2016 May 17;17:363. doi: 10.1186/s12864-016-2693-3 (PMC4869279; doi:10.1186/s12864-016-2693-3)
Supplement: Additional file 10: Figure S2. — Maximum-likelihood phylogeny of Criollo and Arabidopsis PR-1 family members. (PDF 4169 kb) [file 12864_2016_2693_MOESM10_ESM.pdf]

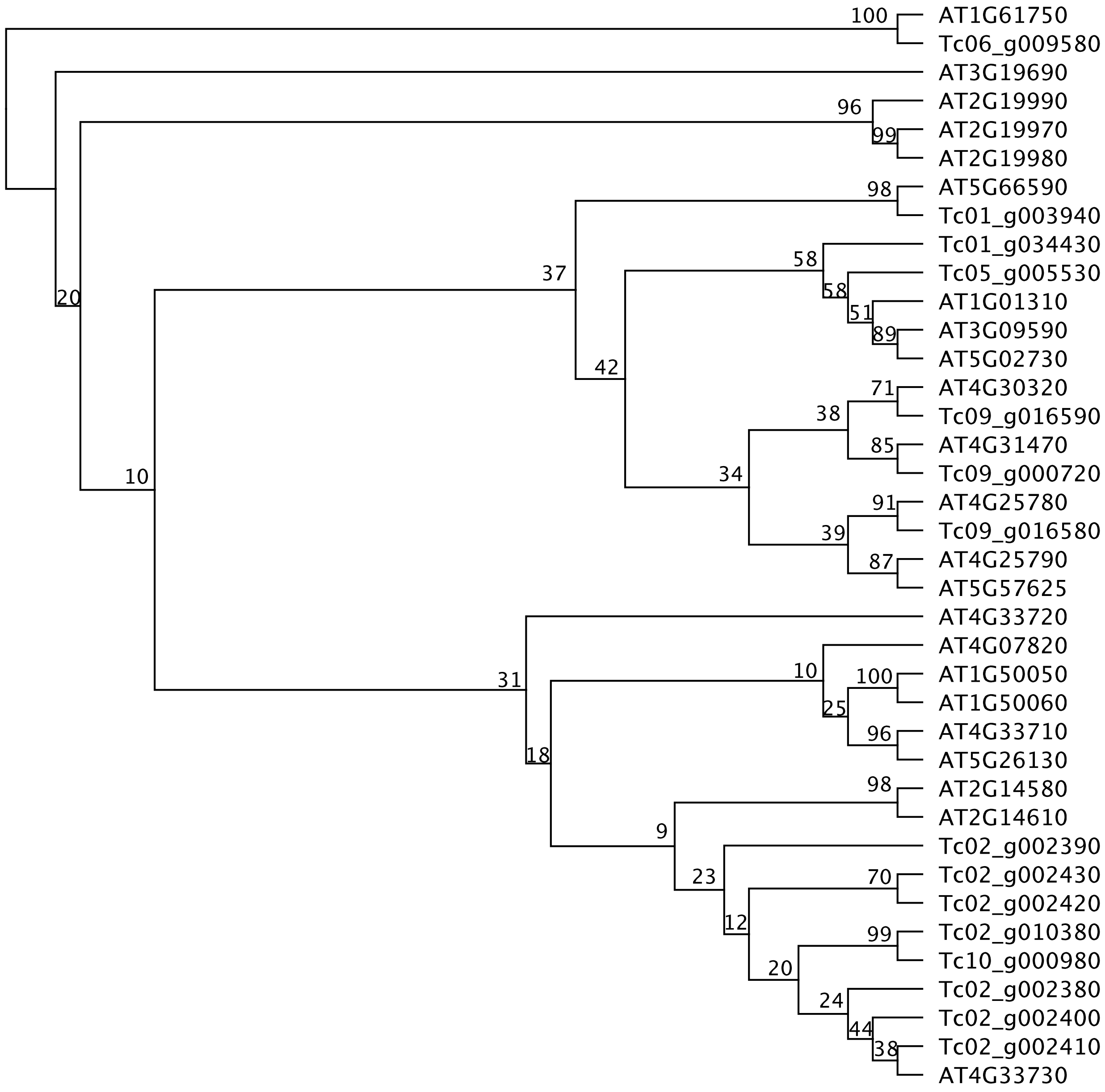

Figure S2. Maximum-likelihood phylogeny of Criollo and Arabidopsis PR-1 family members. Node labels represent bootstrap support from 100 replicates. Branch lengths represent genetic distance in substitutions per site. At1G61750 and Tc06_g009580, two cysteine-rich secretory peptides and members of the SCP superfamily, were included as an outgroup.
