# Supplementary material for: Theobroma cacao L. pathogenesis-related gene tandem array members show diverse expression dynamics in response to pathogen colonization
Source: BMC Genomics. 2016 May 17;17:363. doi: 10.1186/s12864-016-2693-3 (PMC4869279; doi:10.1186/s12864-016-2693-3)
Supplement: Additional file 11: Figure S3. — Maximum-likelihood phylogeny of Criollo and Arabidopsis PR-4 family members. (PDF 4169 kb) [file 12864_2016_2693_MOESM11_ESM.pdf]

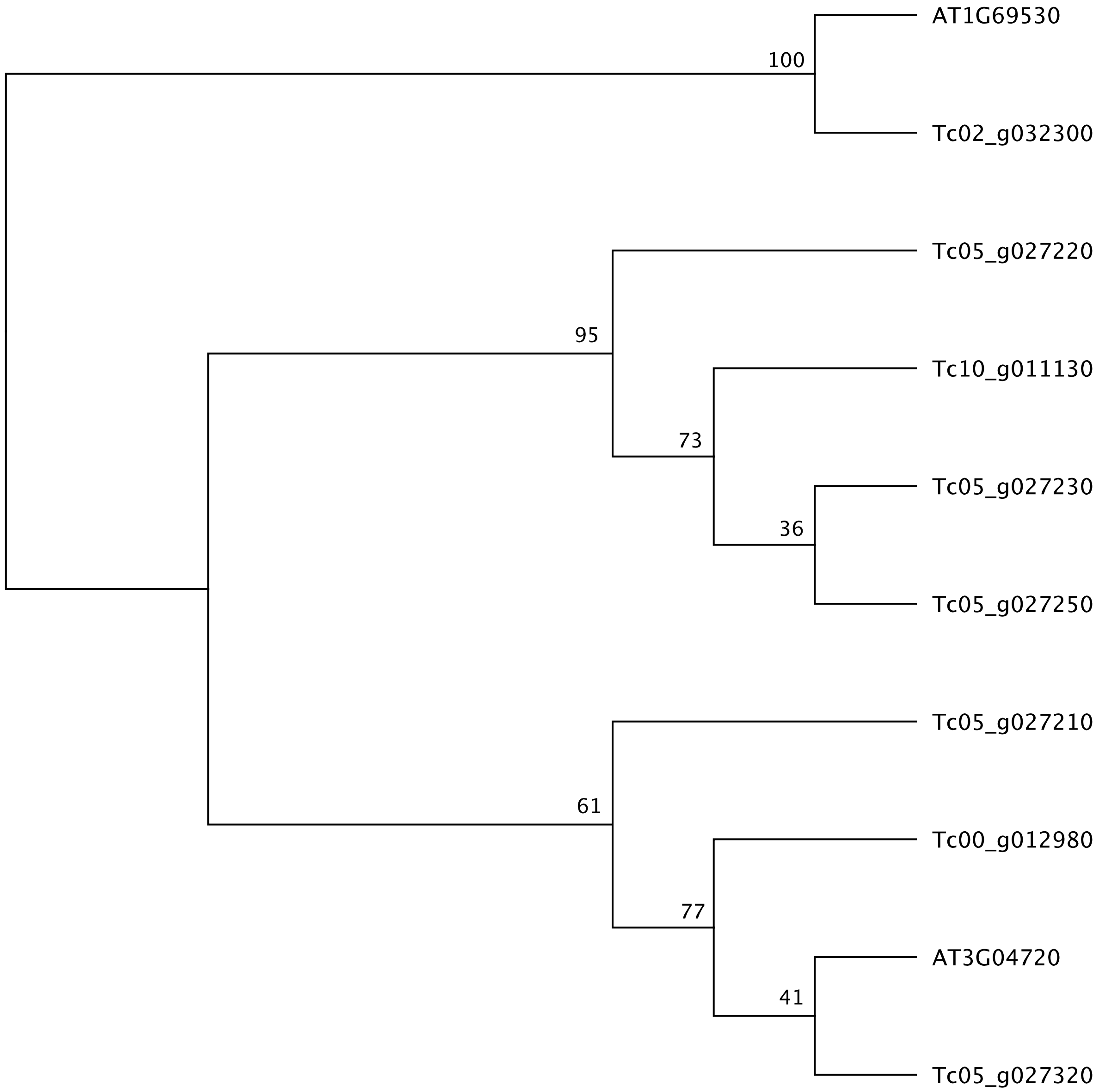

Figure S3. Maximum-likehood phylogeny of Criollo and Arabidopsis PR-4 family members. Node labels represent bootstrap supportf rom 100 replicates. Branch lengths represent genetic distance in substitutions per site. AT5G05460 and Tc02_g032300, two α-expansins that are part members oft he PR-4 superfamily, were included as an outgroup.
