# Supplementary material for: Theobroma cacao L. pathogenesis-related gene tandem array members show diverse expression dynamics in response to pathogen colonization
Source: BMC Genomics. 2016 May 17;17:363. doi: 10.1186/s12864-016-2693-3 (PMC4869279; doi:10.1186/s12864-016-2693-3)
Supplement: Additional file 12: Table S10. — Percent identities for Criollo PR-1 genes, color-coded to show tandem array members. (PDF 4169 kb) [file 12864_2016_2693_MOESM12_ESM.pdf]

Supplemental Table 10 - Percent identify of PR-1 family members. Highlighting in the same color indicates that the genes are grouped in a tandem array.

	Tc02_g002380	Tc02_g002410	Tc02_g002400	Tc02_g002420	Tc02_g010380	Tc02_g002390	Tc01_g034430	Tc02_g002430	Tc05_g005530	Tc09_g016590	Tc09_g016580	Tc09_g000720	Tc01_g003940	Tc10_g000980
Tc02_g002380		70.625	69.167	60.208	58.958	63.502	52.795	57.764	51.235	55.144	48.283	45.342	46.138	43.558
Tc02_g002410	70.625		88.889	62.526	63.395	71.097	51.016	60.772	51.919	50.101	44.643	47.967	44.711	48.394
Tc02_g002400	69.167	88.889		63.561	61.728	67.089	51.329	59.714	51.423	50.407	45.509	48.671	46.185	47.475
Tc02_g002420	60.208	62.526	63.561		55.28	62.42	47.737	67.695	48.262	51.329	45.582	46.296	40.404	44.919
Tc02_g010380	58.958	63.395	61.728	55.28		60.549	41.24	52.929	43.882	47.904	43.367	44.982	43.82	64.815
Tc02_g002390	63.502	71.097	67.089	62.42	60.549		48.428	59.119	48.958	51.667	45.808	49.057	42.593	46.377
Tc01_g034430	52.795	51.016	51.329	47.737	41.24	48.428		51.205	50.08	50.1	43.027	48.566	42.697	38.856
Tc02_g002430	57.764	60.772	59.714	67.695	52.929	59.119	51.205		49.301	48.104	45.098	45.783	41.815	44.98
Tc05_g005530	51.235	51.919	51.423	48.262	43.882	48.958	50.08	49.301		49.701	46.024	44.92	41.341	37.733
Tc09_g016590	55.144	50.101	50.407	51.329	47.904	51.667	50.1	48.104	49.701		52.242	49.701	42.353	36.727
Tc09_g016580	48.283	44.643	45.509	45.582	43.367	45.808	43.027	45.098	46.024	52.242		44.561	46.369	34.893
Tc09_g000720	45.342	47.967	48.671	46.296	44.982	49.057	48.566	45.783	44.92	49.701	44.561		44.007	34.531
Tc01_g003940	46.138	44.711	46.185	40.404	43.82	42.593	42.697	41.815	41.341	42.353	46.369	44.007		33.725
Tc10_g000980	43.558	48.394	47.475	44.919	64.815	46.377	38.856	44.98	37.733	36.727	34.893	34.531	33.725	
