# Supplementary material for: Theobroma cacao L. pathogenesis-related gene tandem array members show diverse expression dynamics in response to pathogen colonization
Source: BMC Genomics. 2016 May 17;17:363. doi: 10.1186/s12864-016-2693-3 (PMC4869279; doi:10.1186/s12864-016-2693-3)
Supplement: Additional file 13: Table S11. — Percent identities for Criollo PR-3 genes, color-coded to show tandem array members (PDF 4169 kb) [file 12864_2016_2693_MOESM13_ESM.pdf]

Supplemental Table S11 - Percent identify of PR-3 family members. Highlighting in the same color indicates that the genes are grouped in a tandem array.											
	Tc01_g000770	Tc02_g003890	Tc01_g000800	Tc01_g032950	Tc01_g010350	Tc04_g018160	Tc06_g000490	Tc04_g029180	Tc04_g018100	Tc04_g018090	Tc04_g018110
Tc01_g000770		63.291	65.657	52.415	60.792	42.749	44.374	42.316	40.684	43.388	39.266
Tc02_g003890	63.291		64.015	53.623	53.061	44.408	42.029	43.565	41.635	43.169	42.345
Tc01_g000800	65.657	64.015		57.987	54.966	42.765	46.424	46.303	42.342	43.372	43.629
Tc01_g032950	52.415	53.623	57.987		49.933	40.247	43.31	44.014	44.613	43.951	41.605
Tc01_g010350	60.792	53.061	54.966	49.933		35.139	43.307	41.47	36.929	39.142	36.653
Tc04_g018160	42.749	44.408	42.765	40.247	35.139		34.093	35.216	58.918	61.728	59.03
Tc06_g000490	44.374	42.029	46.424	43.31	43.307	34.093		71.097	38.235	36.156	33.876
Tc04_g029180	42.316	43.565	46.303	44.014	41.47	35.216	71.097		36.52	36.323	34.773
Tc04_g018100	40.684	41.635	42.342	44.613	36.929	58.918	38.235	36.52		77.047	67.544
Tc04_g018090	43.388	43.169	43.372	43.951	39.142	61.728	36.156	36.323	77.047		68.272
Tc04_g018110	39.266	42.345	43.629	41.605	36.653	59.03	33.876	34.773	67.544	68.272	
