# Supplementary material for: Theobroma cacao L. pathogenesis-related gene tandem array members show diverse expression dynamics in response to pathogen colonization
Source: BMC Genomics. 2016 May 17;17:363. doi: 10.1186/s12864-016-2693-3 (PMC4869279; doi:10.1186/s12864-016-2693-3)
Supplement: Additional file 14: Table S12. — Percent identities for Criollo PR-4 genes, color-coded to show tandem array members. (PDF 4169 kb) [file 12864_2016_2693_MOESM14_ESM.pdf]

Supplemental Table S14 - Percent identify of PR-4 family members. Highlighting in the same color indicates that the genes are grouped in a tandem array.

	Tc05_g027210	Tc00_g012980	Tc05_g027320	Tc05_g027220	Tc05_g027230	Tc10_g011130	Tc05_g027250
Tc05_g027210		68.065	62.005	69.213	66.204	67.361	67.593
Tc00_g012980	68.065		66.323	55.787	49.432	55.324	55.556
Tc05_g027320	62.005	66.323		50.105	45.724	49.686	49.895
Tc05_g027220	69.213	55.787	50.105		90.754	91.727	88.129
Tc05_g027230	66.204	49.432	45.724	90.754		96.215	91.607
Tc10_g011130	67.361	55.324	49.686	91.727	96.215		92.686
Tc05_g027250	67.593	55.556	49.895	88.129	91.607	92.686	
