# Supplementary material for: Theobroma cacao L. pathogenesis-related gene tandem array members show diverse expression dynamics in response to pathogen colonization
Source: BMC Genomics. 2016 May 17;17:363. doi: 10.1186/s12864-016-2693-3 (PMC4869279; doi:10.1186/s12864-016-2693-3)
Supplement: Additional file 15: Table S13. — Log2 normalized expression values for all PR genes on microarray, with values averaged across five biological replicates. (PDF 4168 kb) [file 12864_2016_2693_MOESM15_ESM.pdf]

Supplemental Table S13 - Normalized expression values of probes for PR genes, averaged across replicates					
TcID	Class	Probe	*P. palmivora*-treated mean log_2 normalized expression	*C. theobromicola*-treated mean log_2 normalized expression	Water-treated mean log_2 normalized expression
TC02G002380	PR-1	TC02G002380:1-480	5.53	6.13	5.32
TC02G002400	PR-1	TC02G002400:1-486	6.24	6.08	5.46
TC02G002410	PR-1	TC02G002410_3UTR0:1-186	14.35	13.12	6.75
TC02G002410	PR-1	TC02G002410:1-489	14.86	13.75	8.52
TC02G002420	PR-1	TC02G002420:1-480	5.75	5.54	5.85
TC02G010380	PR-1	TC02G010380:1-1872	5.99	6.21	6.03
TC02G002390	PR-1	TC02G002390:1-471	7.41	6.70	6.10
TC01G034430	PR-1	TC01G034430_3UTR0:1-1150	5.90	5.87	5.75
TC01G034430	PR-1	TC01G034430:1-645	5.31	5.79	5.20
TC02G002430	PR-1	TC02G002430_3UTR1:1-263	10.43	8.47	8.29
TC02G002430	PR-1	TC02G002430:1-495	11.50	9.36	9.22
TC05G005530	PR-1	TC05G005530:1-711	5.58	5.42	5.56
TC09G016590	PR-1	TC09G016590_3UTR0:1-599	5.86	6.35	6.24
TC09G016590	PR-1	TC09G016590:1-501	9.28	9.50	9.59
TC09G016580	PR-1	TC09G016580_3UTR0:1-613	12.23	11.57	12.05
TC09G016580	PR-1	TC09G016580:1-588	12.54	11.87	12.39
TC09G000720	PR-1	TC09G000720:1-558	5.30	5.34	5.36
TC01G003940	PR-1	TC01G003940_3UTR0:1-213	9.68	9.62	10.27
TC01G003940	PR-1	TC01G003940:1-534	7.94	8.28	8.76

TC10G000980	PR-1	TC10G000980 :1-1716	6.97	7.30	6.40
TC09G024130	PR-2	TC09G024130 _3UTR0:1-180	14.45	14.82	11.32
TC09G024130	PR-2	TC09G024130 :1-1038	12.80	13.30	9.75
TC04G029300	PR-2	TC04G029300 _3UTR0:1-151	13.11	11.14	6.31
TC04G029300	PR-2	TC04G029300 :1-1113	14.09	12.23	6.82
TC09G024150	PR-2	TC09G024150 :1-1044	5.79	5.56	5.61
TC09G024140	PR-2	TC09G024140 :1-969	6.19	5.76	5.77
TC09G023540	PR-2	TC09G023540 :1-1119	6.01	6.05	5.80
TC00G083950	PR-2	TC00G083950 :1-1044	12.63	12.42	7.15
TC05G016070	PR-2	TC05G016070 _3UTR0:1-231	9.58	9.93	5.83
TC05G016070	PR-2	TC05G016070 :1-1035	9.45	9.85	6.26
TC03G029620	PR-2	TC03G029620 :1-1026	5.47	5.70	5.28
TC02G023780	PR-2	TC02G023780 :1-999	5.46	5.64	5.30
TC09G008700	PR-2	TC09G008700 _3UTR0:1-469	11.31	11.11	11.52
TC09G008700	PR-2	TC09G008700 _3UTR1:1-287	5.76	6.12	6.34
TC09G008700	PR-2	TC09G008700 :1-1518	10.46	10.36	11.05
TC09G011610	PR-2	TC09G011610 _3UTR0:1-203	12.03	12.21	11.71
TC09G011610	PR-2	TC09G011610 :1-1173	11.52	11.58	11.35
TC08G009900	PR-2	TC08G009900 :1-1014	5.07	5.51	5.40
TC06G001730	PR-2	TC06G001730 :1-1251	6.44	6.72	6.64
TC00G054290	PR-2	TC00G054290 _3UTR0:1-88	12.18	12.49	12.10
TC00G054290	PR-2	TC00G054290 _3UTR1:1-328	12.32	12.56	11.97
TC00G054290	PR-2	TC00G054290 :1-1104	11.85	12.10	11.79
TC04G012520	PR-2	TC04G012520	9.12	9.58	10.10

		_3UTR0:1-283			
TC04G012520	PR-2	TC04G012520 :1-1497	8.73	9.23	9.74
TC01G017650	PR-2	TC01G017650 _3UTR0:1-499	6.12	6.66	6.48
TC01G017650	PR-2	TC01G017650 :1-1422	9.80	9.21	9.54
TC02G007080	PR-2	TC02G007080 _3UTR0:1-336	13.34	13.46	12.92
TC02G007080	PR-2	TC02G007080 :1-1380	14.34	14.38	13.98
TC08G001980	PR-2	TC08G001980 _3UTR0:1-390	11.93	11.95	12.17
TC08G001980	PR-2	TC08G001980 :1-1395	11.54	11.43	11.82
TC03G022650	PR-2	TC03G022650 _3UTR0:1-174	7.65	7.73	8.49
TC03G022650	PR-2	TC03G022650 :1-1227	13.43	13.55	13.78
TC03G021200	PR-2	TC03G021200 _3UTR0:1-287	7.40	6.96	7.26
TC03G021200	PR-2	TC03G021200 :1-1455	7.08	6.87	7.16
TC09G031660	PR-2	TC09G031660 _3UTR0:1-338	9.55	9.32	8.54
TC09G031660	PR-2	TC09G031660 :1-1494	11.11	10.90	10.18
TC07G002650	PR-2	TC07G002650 _3UTR0:1-261	10.18	10.40	10.33
TC07G002650	PR-2	TC07G002650 :1-1401	10.83	10.95	10.79
TC06G012580	PR-2	TC06G012580 :1-1209	6.61	6.49	6.63
TC02G028070	PR-2	TC02G028070 _3UTR0:1-359	7.37	7.57	8.38
TC02G028070	PR-2	TC02G028070 :1-1566	7.76	7.36	8.22
TC06G019320	PR-2	TC06G019320 _3UTR0:1-385	8.63	8.29	8.30
TC06G019320	PR-2	TC06G019320 :1-1575	11.77	11.87	11.62
TC01G021070	PR-2	TC01G021070 _3UTR0:1-418	13.56	13.29	13.08
TC01G021070	PR-2	TC01G021070 :1-1386	12.95	12.64	12.51
TC09G013490	PR-2	TC09G013490 _3UTR0:1-	12.40	12.47	12.44

		2851			
TC09G013490	PR-2	TC09G013490 :1-1341	12.23	12.15	12.23
TC10G003270	PR-2	TC10G003270 :1-1371	6.41	5.59	5.75
TC08G005240	PR-2	TC08G005240 _3UTR0:1-498	6.04	5.54	6.24
TC08G005240	PR-2	TC08G005240 :1-1386	7.15	6.56	7.40
TC01G010310	PR-2	TC01G010310 :1-1425	5.75	6.04	5.53
TC00G034720	PR-2	TC00G034720 _3UTR0:1-208	11.42	11.46	11.77
TC00G034720	PR-2	TC00G034720 :1-1479	9.71	10.03	10.08
TC04G020310	PR-2	TC04G020310 _3UTR0:1-220	6.17	6.29	6.16
TC04G020310	PR-2	TC04G020310 :1-1473	6.70	6.48	6.64
TC05G028370	PR-2	TC05G028370 :1-1440	11.83	11.94	11.77
TC09G021600	PR-2	TC09G021600 _3UTR0:1-364	10.00	10.10	9.69
TC09G021600	PR-2	TC09G021600 :1-1491	8.29	8.58	8.58
TC09G010240	PR-2	TC09G010240 :1-1569	5.80	5.84	5.91
TC04G023620	PR-2	TC04G023620 _3UTR0:1-131	5.54	6.00	5.65
TC04G023620	PR-2	TC04G023620 :1-3063	8.03	7.62	8.11
TC09G034460	PR-2	TC09G034460 :1-1545	6.59	6.64	6.98
TC01G014030	PR-2	TC01G014030 _3UTR0:1-215	11.30	11.41	11.19
TC01G014030	PR-2	TC01G014030 :1-1470	12.53	12.43	12.12
TC01G010320	PR-2	TC01G010320 :1-1614	5.41	5.46	5.50
TC09G000150	PR-2	TC09G000150 _3UTR0:1-94	10.37	10.52	11.00
TC09G000150	PR-2	TC09G000150 :1-1461	11.50	11.63	11.82
TC09G006080	PR-2	TC09G006080 _3UTR0:1-124	7.86	7.81	6.98
TC09G006080	PR-2	TC09G006080 :1-1473	6.83	7.03	6.49

TC10G002190	PR-2	TC10G002190:1-1254	5.61	5.47	5.29
TC01G006420	PR-2	TC01G006420_3UTR0:1-247	6.15	6.94	6.81
TC01G006420	PR-2	TC01G006420:1-1293	6.17	5.88	6.27
TC01G000770	PR-3	TC01G000770:1-936	13.08	12.25	8.02
TC02G003890	PR-3	TC02G003890_3UTR0:1-144	12.70	12.46	7.70
TC02G003890	PR-3	TC02G003890:1-957	12.80	12.66	8.28
TC01G000800	PR-3	TC01G000800_3UTR0:1-236	9.12	5.71	5.60
TC01G000800	PR-3	TC01G000800:1-792	10.39	7.33	5.49
TC01G032950	PR-3	TC01G032950_3UTR0:1-160	8.69	8.25	6.91
TC01G032950	PR-3	TC01G032950:1-825	8.12	7.46	6.57
TC01G010350	PR-3	TC01G010350:1-717	5.75	5.84	5.48
TC04G018160	PR-3	TC04G018160_3UTR0:1-251	13.81	14.02	7.88
TC04G018160	PR-3	TC04G018160:1-819	12.85	13.05	6.81
TC06G000490	PR-3	TC06G000490_3UTR0:1-341	15.28	15.35	15.14
TC06G000490	PR-3	TC06G000490:1-969	14.94	15.14	14.82
TC04G029180	PR-3	TC04G029180_3UTR0:1-286	14.36	14.19	13.26
TC04G029180	PR-3	TC04G029180:1-948	15.16	14.80	14.02
TC04G018090	PR-3	TC04G018090:1-807	10.73	10.24	8.48
TC04G018100	PR-3	TC04G018100_3UTR0:1-113	7.07	6.29	6.36
TC04G018100	PR-3	TC04G018100:1-675	11.13	9.70	6.64
TC04G018110	PR-3	TC04G018110:1-816	13.34	12.00	8.48
TC05G027210	PR-4	TC05G027210_3UTR0:1-254	9.95	8.16	6.15
TC05G027210	PR-4	TC05G027210:1-429	13.41	11.85	7.94
TC00G012980	PR-4	TC00G012980	5.41	5.64	5.21

		_3UTR0:1-2087			
TC00G012980	PR-4	TC00G012980:1-582	9.11	8.42	6.89
TC05G027320	PR-4	TC05G027320_3UTR0:1-110	11.68	10.05	5.84
TC05G027320	PR-4	TC05G027320:1-624	11.51	9.96	5.87
TC05G027220	PR-4	TC05G027220_3UTR0:1-247	12.92	12.11	9.37
TC05G027220	PR-4	TC05G027220:1-816	15.23	14.41	11.84
TC10G011130	PR-4	TC10G011130_3UTR0:1-422	8.05	7.50	7.33
TC10G011130	PR-4	TC10G011130:1-819	12.94	12.09	10.92
TC05G027230	PR-4	TC05G027230:1-915	12.60	11.63	10.48
TC05G027250	PR-4	TC05G027250_3UTR0:1-1004	6.20	5.77	6.20
TC05G027250	PR-4	TC05G027250:1-834	15.55	15.03	13.60
TC05G027340	PR-4	TC05G027340:1-3459	5.84	5.61	5.85
TC03G005540	PR-5	TC03G005540_3UTR0:1-213	10.36	9.98	9.83
TC03G005540	PR-5	TC03G005540:1-750	10.03	9.49	9.58
TC01G001580	PR-5	TC01G001580:1-954	12.32	12.21	12.77
TC08G003730	PR-5	TC08G003730_3UTR0:1-1467	6.07	6.13	5.98
TC08G003730	PR-5	TC08G003730:1-963	14.12	14.12	13.90
TC02G005190	PR-5	TC02G005190_3UTR0:1-418	10.27	10.03	10.21
TC02G005190	PR-5	TC02G005190:1-1086	11.51	11.04	11.26
TC08G001670	PR-5	TC08G001670_3UTR0:1-185	9.30	9.06	8.46
TC08G001670	PR-5	TC08G001670:1-744	9.23	8.94	8.54
TC01G001590	PR-5	TC01G001590_3UTR0:1-596	9.29	8.79	8.92
TC01G001590	PR-5	TC01G001590	9.13	8.25	8.30

		:1-870			
TC00G056050	PR-5	TC00G056050_3UTR0:1-2172	5.49	5.46	5.36
TC00G056050	PR-5	TC00G056050:1-726	6.04	6.73	6.26
TC00G056070	PR-5	TC00G056070_3UTR0:1-338	10.31	5.97	6.00
TC00G056070	PR-5	TC00G056070:1-732	13.10	9.21	8.51
TC08G010190	PR-5	TC08G010190:1-852	6.33	6.08	6.35
TC00G056060	PR-5	TC00G056060_3UTR0:1-605	5.68	5.37	5.67
TC00G056060	PR-5	TC00G056060:1-729	5.30	5.33	5.38
TC04G020870	PR-5	TC04G020870_3UTR0:1-317	5.78	6.25	6.33
TC04G020870	PR-5	TC04G020870:1-882	6.08	6.22	5.81
TC02G005200	PR-5	TC02G005200_3UTR0:1-689	6.74	6.17	6.50
TC02G005200	PR-5	TC02G005200:1-945	10.75	10.49	10.82
TC05G004240	PR-5	TC05G004240_3UTR0:1-292	7.88	7.42	7.60
TC05G004240	PR-5	TC05G004240:1-954	6.03	5.82	5.91
TC08G003740	PR-5	TC08G003740:1-942	10.20	9.48	9.00
TC10G002890	PR-5	TC10G002890_3UTR0:1-248	9.31	7.80	9.17
TC10G002890	PR-5	TC10G002890:1-747	8.05	7.69	8.23
TC02G003020	PR-5	TC02G003020:1-1017	7.83	7.72	7.90
TC04G008530	PR-5	TC04G008530_3UTR0:1-572	8.18	7.97	7.40
TC04G008530	PR-5	TC04G008530:1-765	10.33	10.12	9.25
TC03G027030	PR-5	TC03G027030_3UTR0:1-305	14.70	14.86	11.79
TC03G027030	PR-5	TC03G027030:1-723	13.86	13.85	10.67
TC09G031980	PR-5	TC09G031980_3UTR0:1-557	6.97	7.39	6.98
TC09G031980	PR-5	TC09G031980	11.51	11.15	10.72

		:1-762			
TC03G026960	PR-5	TC03G026960 _3UTR0:1-133	11.32	11.97	11.46
TC03G026960	PR-5	TC03G026960 :1-678	10.65	11.19	10.81
TC00G060970	PR-5	TC00G060970 :1-675	9.40	10.45	6.22
TC03G027010	PR-5	TC03G027010 _3UTR0:1-241	11.21	9.58	5.47
TC03G027010	PR-5	TC03G027010 :1-675	12.99	11.43	6.08
TC03G026990	PR-5	TC03G026990 _3UTR0:1-173	8.55	7.61	5.37
TC03G026990	PR-5	TC03G026990 :1-675	10.51	9.94	5.86
TC00G056110	PR-5	TC00G056110 :1-756	5.98	6.28	5.66
TC03G027000	PR-5	TC03G027000 _3UTR0:1-134	6.32	7.32	6.14
TC03G027000	PR-5	TC03G027000 :1-585	8.04	8.40	5.85
TC03G026980	PR-5	TC03G026980 :1-528	5.93	5.88	5.97
TC09G016370	PR-5	TC09G016370 :1-456	5.67	5.66	5.81
TC04G014480	PR-5	TC04G014480 :1-375	5.95	5.88	5.85
TC03G027020	PR-5	TC03G027020 _3UTR0:1-291	5.53	5.50	5.49
TC03G027020	PR-5	TC03G027020 :1-327	5.38	5.84	5.76
TC10G005920	PR-6	TC10G005920 _3UTR0:1-182	12.31	10.29	6.33
TC10G005920	PR-6	TC10G005920 :1-594	12.32	10.71	6.99
TC10G005840	PR-6	TC10G005840 _3UTR0:1-975	7.99	6.05	5.27
TC10G005840	PR-6	TC10G005840 :1-216	12.09	10.07	7.15
TC10G005870	PR-6	TC10G005870 _3UTR0:1-179	9.00	7.14	5.42
TC10G005870	PR-6	TC10G005870 :1-216	13.04	11.39	7.49
TC10G005890	PR-6	TC10G005890 :1-216	9.70	7.81	6.02
TC05G022770	PR-6	TC05G022770 _3UTR0:1-398	11.20	11.38	9.76

TC05G022770	PR-6	TC05G022770<br>:1-483	15.23	15.32	14.05
TC05G022780	PR-6	TC05G022780<br>_3UTR0:1-641	15.07	15.19	14.56
TC05G022780	PR-6	TC05G022780<br>:1-483	15.22	15.27	14.77
TC03G026330	PR-7	TC03G026330<br>_3UTR0:1-609	5.77	5.81	5.97
TC03G026330	PR-7	TC03G026330<br>:1-2232	5.40	5.40	5.37
TC03G026340	PR-7	TC03G026340<br>:1-1887	5.48	5.53	5.77
TC01G037030	PR-7	TC01G037030<br>:1-2307	12.64	12.29	10.31
TC01G026170	PR-7	TC01G026170<br>_3UTR1:1-332	5.72	5.93	5.94
TC01G026170	PR-7	TC01G026170<br>:1-2298	5.48	5.90	5.54
TC01G037020	PR-7	TC01G037020<br>_3UTR0:1-134	11.10	11.17	9.07
TC01G037020	PR-7	TC01G037020<br>:1-2418	8.83	9.13	7.27
TC02G005130	PR-7	TC02G005130<br>_3UTR0:1-432	11.49	11.38	11.46
TC02G005130	PR-7	TC02G005130<br>:1-2325	13.66	13.76	13.89
TC01G006160	PR-7	TC01G006160<br>_3UTR0:1-274	13.25	13.33	13.13
TC01G006160	PR-7	TC01G006160<br>:1-2283	13.31	13.24	13.24
TC01G037010	PR-7	TC01G037010<br>:1-4701	11.55	11.89	11.28
TC09G006830	PR-7	TC09G006830<br>_3UTR0:1-454	8.34	8.05	7.79
TC09G006830	PR-7	TC09G006830<br>:1-2280	8.35	8.28	7.87
TC00G013470	PR-7	TC00G013470<br>_3UTR0:1-282	11.96	11.92	11.84
TC00G013470	PR-7	TC00G013470<br>:1-2304	10.67	10.75	10.70
TC08G007740	PR-7	TC08G007740<br>_3UTR0:1-266	5.73	5.49	5.77
TC08G007740	PR-7	TC08G007740<br>:1-2631	11.91	11.44	11.50
TC03G018930	PR-7	TC03G018930<br>_3UTR0:1-238	12.91	13.16	13.05
TC03G018930	PR-7	TC03G018930	12.24	12.58	12.59

		:1-2337			
TC06G010630	PR-7	TC06G010630 _3UTR0:1-351	12.17	12.04	12.10
TC06G010630	PR-7	TC06G010630 :1-2319	11.19	11.04	11.22
TC08G000270	PR-7	TC08G000270 _3UTR0:1-188	12.77	12.45	12.15
TC08G000270	PR-7	TC08G000270 :1-2283	10.28	9.99	9.97
TC07G000490	PR-7	TC07G000490 _3UTR0:1-134	6.54	6.27	7.49
TC07G000490	PR-7	TC07G000490 :1-2313	6.79	6.50	7.30
TC03G022570	PR-7	TC03G022570 _3UTR0:1-130	10.26	10.33	9.98
TC03G022570	PR-7	TC03G022570 :1-2331	10.77	10.88	10.83
TC06G013520	PR-7	TC06G013520 :1-2415	9.28	9.74	9.83
TC00G032610	PR-7	TC00G032610 :1-2469	12.05	12.47	12.69
TC10G002320	PR-7	TC10G002320 :1-6357	5.44	5.80	5.59
TC08G000240	PR-7	TC08G000240 :1-2205	5.30	6.00	5.52
TC08G000230	PR-7	TC08G000230 :1-2298	5.76	6.59	7.10
TC01G000090	PR-7	TC01G000090 :1-2286	7.93	8.15	8.56
TC10G002300	PR-7	TC10G002300 :1-2304	5.56	5.48	5.59
TC05G013470	PR-7	TC05G013470 _3UTR0:1-2305	5.27	5.35	5.25
TC05G013470	PR-7	TC05G013470 :1-2301	5.48	5.21	5.42
TC03G026560	PR-7	TC03G026560 :1-2325	5.59	5.60	5.47
TC01G005210	PR-7	TC01G005210 _3UTR0:1-96	5.76	5.56	5.71
TC01G005210	PR-7	TC01G005210 _3UTR1:1-173	7.42	6.42	6.62
TC01G005210	PR-7	TC01G005210 :1-2400	5.56	5.53	5.31
TC08G004000	PR-7	TC08G004000 :1-2343	7.16	7.52	7.13
TC03G026570	PR-7	TC03G026570	5.52	5.92	5.50

		:1-2328			
TC08G000260	PR-7	TC08G000260 :1-2382	11.09	10.85	11.09
TC03G022590	PR-7	TC03G022590 :1-2358	7.25	7.74	6.15
TC01G005220	PR-7	TC01G005220 _3UTR0:1-72	6.33	6.86	7.12
TC01G005220	PR-7	TC01G005220 :1-2268	5.73	5.53	5.84
TC10G002310	PR-7	TC10G002310 _3UTR0:1-80	5.96	6.13	5.97
TC10G002310	PR-7	TC10G002310 :1-2136	5.81	6.24	5.72
TC09G028480	PR-7	TC09G028480 :1-2679	5.35	5.11	5.41
TC03G021760	PR-7	TC03G021760 :1-2358	5.87	5.67	5.80
TC09G032690	PR-7	TC09G032690 :1-2178	5.66	5.65	5.64
TC09G032720	PR-7	TC09G032720 :1-2139	5.40	5.46	5.57
TC00G001690	PR-7	TC00G001690 :1-2262	8.39	8.38	8.52
TC09G032710	PR-7	TC09G032710 :1-2235	5.25	5.54	5.64
TC01G037040	PR-7	TC01G037040 _3UTR0:1-176	7.59	7.62	7.95
TC01G037040	PR-7	TC01G037040 :1-2316	8.47	8.37	8.41
TC05G010230	PR-7	TC05G010230 :1-2331	5.30	5.54	5.89
TC00G007300	PR-7	TC00G007300 _3UTR0:1-340	6.48	6.54	6.01
TC00G007300	PR-7	TC00G007300 :1-2352	6.88	6.56	6.51
TC02G007300	PR-7	TC02G007300 _3UTR0:1-199	7.17	7.61	7.16
TC02G007300	PR-7	TC02G007300 :1-2406	6.97	6.94	6.78
TC03G022580	PR-7	TC03G022580 :1-2367	9.80	8.73	6.03
TC10G002340	PR-7	TC10G002340 :1-2625	9.08	9.19	8.56
TC08G000250	PR-7	TC08G000250 :1-2172	5.33	5.26	6.24
TC00G038560	PR-7	TC00G038560 _3UTR0:1-285	9.85	9.39	9.58

TC00G038560	PR-7	TC00G038560<br>:1-2457	11.95	11.60	11.67
TC06G000810	PR-7	TC06G000810<br>_3UTR0:1-213	10.13	10.20	10.78
TC06G000810	PR-7	TC06G000810<br>:1-2568	10.92	11.07	11.61
TC00G017310	PR-7	TC00G017310<br>:1-1416	5.39	5.50	5.33
TC03G026320	PR-7	TC03G026320<br>:1-2226	6.09	5.99	6.27
TC09G028050	PR-7	TC09G028050<br>:1-1155	5.69	5.48	5.49
TC06G019800	PR-7	TC06G019800<br>:1-2439	5.39	5.74	5.30
TC03G024170	PR-7	TC03G024170<br>:1-7845	5.26	5.70	5.60
TC06G021130	PR-7	TC06G021130<br>:1-1620	5.43	5.72	5.53
TC01G035050	PR-8	TC01G035050<br>_3UTR0:1-227	10.61	11.11	7.03
TC01G035050	PR-8	TC01G035050<br>:1-891	10.65	10.93	7.29
TC01G035150	PR-8	TC01G035150<br>:1-897	5.59	5.82	5.57
TC00G024510	PR-8	TC00G024510<br>:1-897	5.41	5.46	5.61
TC03G017760	PR-8	TC03G017760<br>:1-903	11.58	11.74	6.55
TC01G035160	PR-8	TC01G035160<br>:1-903	5.31	5.11	5.61
TC03G017780	PR-8	TC03G017780<br>_3UTR0:1-198	6.16	5.25	5.67
TC03G017780	PR-8	TC03G017780<br>:1-897	6.01	5.56	5.75
TC10G015260	PR-8	TC10G015260<br>:1-930	5.83	5.79	5.98
TC10G015330	PR-8	TC10G015330<br>_3UTR0:1-307	6.78	6.85	6.18
TC10G015330	PR-8	TC10G015330<br>:1-918	7.37	7.48	6.19
TC01G032120	PR-8	TC01G032120<br>:1-918	8.73	8.19	7.95
TC03G017790	PR-8	TC03G017790<br>:1-894	5.71	5.90	5.73
TC01G035140	PR-8	TC01G035140<br>:1-846	8.10	9.18	8.73
TC04G001620	PR-8	TC04G001620	5.30	5.45	5.57

		:1-828			
TC01G035060	PR-8	TC01G035060 :1-918	5.63	5.38	6.17
TC01G032260	PR-8	TC01G032260 _3UTR0:1-737	5.38	5.32	5.44
TC01G032260	PR-8	TC01G032260 :1-324	5.52	5.64	5.65
TC00G045940	PR-9	TC00G045940 _3UTR0:1-233	10.36	10.50	9.92
TC00G045940	PR-9	TC00G045940 :1-996	10.62	10.39	10.17
TC00G045400	PR-9	TC00G045400 _3UTR0:1-164	10.30	8.55	5.62
TC00G045400	PR-9	TC00G045400 :1-960	12.43	10.86	6.15
TC00G045440	PR-9	TC00G045440 :1-969	5.52	6.49	5.49
TC00G014230	PR-9	TC00G014230 _3UTR0:1-464	15.63	15.32	14.69
TC00G014230	PR-9	TC00G014230 :1-1041	14.86	14.28	13.49
TC00G045610	PR-9	TC00G045610 _3UTR0:1-117	10.44	10.49	6.57
TC00G045610	PR-9	TC00G045610 :1-969	12.35	12.26	8.54
TC00G045360	PR-9	TC00G045360 :1-1077	5.02	5.63	5.10
TC01G002490	PR-9	TC01G002490 _3UTR0:1-247	5.90	5.46	5.68
TC01G002490	PR-9	TC01G002490 :1-1002	8.12	7.44	7.12
TC02G008380	PR-9	TC02G008380 :1-1005	6.11	5.76	5.56
TC10G016040	PR-9	TC10G016040 _3UTR0:1-276	11.43	9.87	10.70
TC10G016040	PR-9	TC10G016040 :1-954	12.67	11.26	11.39
TC00G040640	PR-9	TC00G040640 :1-963	7.27	7.38	5.60
TC09G034910	PR-9	TC09G034910 :1-963	7.08	5.59	5.45
TC01G004590	PR-9	TC01G004590 _3UTR0:1-109	11.58	11.74	9.72
TC01G004590	PR-9	TC01G004590 :1-984	11.49	11.67	9.69
TC10G016080	PR-9	TC10G016080 _3UTR0:1-179	13.61	13.58	12.22

TC10G016080	PR-9	TC10G016080 :1-1002	14.85	14.67	13.50
TC09G034930	PR-9	TC09G034930 _3UTR0:1-307	14.21	12.76	11.04
TC09G034930	PR-9	TC09G034930 :1-981	13.97	12.73	10.97
TC10G015970	PR-9	TC10G015970 _3UTR0:1-230	13.24	13.14	11.24
TC10G015970	PR-9	TC10G015970 :1-966	12.49	12.41	10.72
TC02G029020	PR-9	TC02G029020 :1-990	5.25	6.28	7.42
TC08G002680	PR-9	TC08G002680 _3UTR0:1-133	5.29	5.74	5.45
TC08G002680	PR-9	TC08G002680 :1-1047	6.00	5.99	5.72
TC02G029090	PR-9	TC02G029090 :1-984	6.69	5.35	5.23
TC04G029820	PR-9	TC04G029820 :1-999	6.10	6.02	6.28
TC09G034950	PR-9	TC09G034950 _3UTR0:1-621	5.92	5.73	5.82
TC09G034950	PR-9	TC09G034950 :1-978	6.60	6.08	6.53
TC02G029120	PR-9	TC02G029120 :1-993	5.36	5.44	5.74
TC02G029110	PR-9	TC02G029110 :1-993	5.18	5.04	5.23
TC03G023600	PR-9	TC03G023600 _3UTR0:1-1225	5.51	5.40	5.41
TC03G023600	PR-9	TC03G023600 :1-996	5.73	5.59	5.76
TC00G045630	PR-9	TC00G045630 :1-954	8.10	5.77	5.88
TC04G016340	PR-9	TC04G016340 :1-951	7.64	6.00	6.19
TC04G016740	PR-9	TC04G016740 :1-951	7.97	5.44	5.77
TC04G027220	PR-9	TC04G027220 _3UTR0:1-159	6.73	6.58	6.06
TC04G027220	PR-9	TC04G027220 :1-1005	5.31	5.40	5.51
TC02G030420	PR-9	TC02G030420 _3UTR1:1-271	10.75	10.26	9.04
TC02G030420	PR-9	TC02G030420 :1-981	9.76	9.48	8.32

TC08G013300	PR-9	TC08G013300_3UTR0:1-3841	5.54	5.52	5.68
TC08G013300	PR-9	TC08G013300_3UTR1:1-660	5.49	5.51	5.32
TC08G013300	PR-9	TC08G013300:1-1068	14.16	13.45	12.17
TC04G027230	PR-9	TC04G027230:1-984	5.48	5.34	5.37
TC04G016760	PR-9	TC04G016760:1-906	8.18	5.93	5.92
TC01G008530	PR-9	TC01G008530_3UTR0:1-219	7.78	7.42	7.82
TC01G008530	PR-9	TC01G008530:1-972	6.88	7.02	7.09
TC03G019180	PR-9	TC03G019180_3UTR0:1-85	6.41	6.05	5.94
TC03G019180	PR-9	TC03G019180:1-975	5.77	5.53	6.10
TC02G012000	PR-9	TC02G012000_3UTR0:1-380	11.60	10.89	9.11
TC02G012000	PR-9	TC02G012000:1-981	10.57	10.05	8.63
TC02G012020	PR-9	TC02G012020:1-978	6.96	7.60	5.69
TC02G011960	PR-9	TC02G011960_3UTR0:1-236	5.95	5.89	5.89
TC02G011960	PR-9	TC02G011960:1-981	6.35	6.28	6.40
TC02G030430	PR-9	TC02G030430_3UTR0:1-247	6.55	6.63	7.16
TC02G030430	PR-9	TC02G030430:1-987	6.99	7.06	7.95
TC01G013330	PR-9	TC01G013330_3UTR0:1-96	10.50	9.86	9.42
TC01G013330	PR-9	TC01G013330:1-951	8.30	7.66	7.38
TC08G004060	PR-9	TC08G004060_3UTR0:1-216	14.99	14.74	14.20
TC08G004060	PR-9	TC08G004060:1-951	14.94	14.49	13.71
TC01G001190	PR-9	TC01G001190_3UTR0:1-263	13.05	12.47	11.99
TC01G001190	PR-9	TC01G001190:1-951	12.46	11.76	11.47
TC02G011940	PR-9	TC02G011940:1-987	5.54	6.11	6.18

TC02G011950	PR-9	TC02G011950 _3UTR0:1-208	5.99	7.25	5.47
TC02G011950	PR-9	TC02G011950 :1-975	6.65	7.70	6.32
TC05G031640	PR-9	TC05G031640 _3UTR0:1-220	8.35	5.46	5.03
TC05G031640	PR-9	TC05G031640 :1-990	9.40	6.26	5.51
TC04G004360	PR-9	TC04G004360 _3UTR0:1-119	5.31	5.13	5.48
TC04G004360	PR-9	TC04G004360 :1-972	5.54	5.14	5.47
TC02G011920	PR-9	TC02G011920 _3UTR0:1-114	5.80	5.58	6.78
TC02G011920	PR-9	TC02G011920 :1-987	6.11	5.52	6.67
TC02G011930	PR-9	TC02G011930 _3UTR0:1-164	6.27	6.12	6.20
TC02G011930	PR-9	TC02G011930 _3UTR1:1-525	5.71	5.96	5.67
TC02G011930	PR-9	TC02G011930 :1-978	5.37	5.28	5.67
TC04G004370	PR-9	TC04G004370 _3UTR0:1-200	7.58	8.43	6.80
TC04G004370	PR-9	TC04G004370 :1-975	7.38	8.63	6.84
TC04G004350	PR-9	TC04G004350 :1-972	5.38	5.49	5.64
TC02G003150	PR-9	TC02G003150 _3UTR0:1-314	10.94	10.91	10.91
TC02G003150	PR-9	TC02G003150 :1-957	12.46	12.67	12.54
TC04G028060	PR-9	TC04G028060 :1-984	5.19	5.38	5.50
TC04G019840	PR-9	TC04G019840 :1-1098	5.68	5.38	5.57
TC00G045460	PR-9	TC00G045460 _3UTR0:1-280	14.93	14.50	13.63
TC00G045460	PR-9	TC00G045460 :1-861	9.89	9.14	8.33
TC00G054480	PR-9	TC00G054480 :1-1005	5.70	5.86	5.51
TC09G000620	PR-9	TC09G000620 :1-975	6.42	6.30	6.45
TC02G022830	PR-9	TC02G022830 :1-996	5.56	5.57	5.69
TC00G020190	PR-9	TC00G020190	8.54	9.44	9.00

		:1-978			
TC03G021880	PR-9	TC03G021880 _3UTR0:1-116	13.33	13.45	13.29
TC03G021880	PR-9	TC03G021880 :1-993	12.06	12.29	12.04
TC09G001160	PR-9	TC09G001160 _3UTR0:1-228	5.44	5.62	6.15
TC09G001160	PR-9	TC09G001160 :1-996	5.76	5.67	6.94
TC08G012700	PR-9	TC08G012700 _3UTR0:1-1188	5.53	5.45	5.59
TC08G012700	PR-9	TC08G012700 :1-1008	11.16	10.06	10.74
TC01G006280	PR-9	TC01G006280 _3UTR0:1-358	14.40	14.21	13.87
TC01G006280	PR-9	TC01G006280 :1-993	14.54	14.43	13.89
TC03G000530	PR-9	TC03G000530 _3UTR0:1-121	8.52	8.64	8.44
TC03G000530	PR-9	TC03G000530 :1-993	5.88	6.40	6.89
TC09G017500	PR-9	TC09G017500 :1-1446	5.47	5.26	5.35
TC06G008540	PR-9	TC06G008540 :1-1335	6.16	6.11	6.79
TC04G000340	PR-9	TC04G000340 :1-978	11.09	11.08	11.18
TC06G019650	PR-9	TC06G019650 :1-993	5.73	5.87	5.76
TC01G032570	PR-9	TC01G032570 :1-975	6.74	7.65	7.66
TC05G006120	PR-9	TC05G006120 _3UTR0:1-145	8.86	6.90	6.01
TC05G006120	PR-9	TC05G006120 :1-987	9.22	7.95	6.64
TC02G013360	PR-9	TC02G013360 :1-930	5.43	5.58	5.84
TC01G029350	PR-9	TC01G029350 :1-1158	5.29	5.45	5.33
TC04G029620	PR-9	TC04G029620 :1-972	5.52	5.62	5.82
TC09G011360	PR-9	TC09G011360 :1-885	6.34	5.78	6.91
TC06G019150	PR-9	TC06G019150 _3UTR0:1-704	9.86	9.91	10.05
TC06G019150	PR-9	TC06G019150	14.86	14.59	14.95

		:1-990			
TC00G044710	PR-9	TC00G044710 :1-2598	6.16	6.28	6.08
TC02G030980	PR-9	TC02G030980 :1-618	6.50	7.00	7.16
TC06G014950	PR-9	TC06G014950 _3UTR0:1-294	6.06	6.62	7.54
TC06G014950	PR-9	TC06G014950 :1-1323	5.62	5.99	6.35
TC04G016710	PR-9	TC04G016710 :1-2412	8.93	9.16	8.86
TC10G016070	PR-9	TC10G016070 :1-708	8.15	7.41	8.21
TC02G030970	PR-9	TC02G030970 _3UTR0:1-208	8.07	8.11	8.20
TC02G030970	PR-9	TC02G030970 :1-438	7.56	7.54	8.27
TC02G011990	PR-9	TC02G011990 :1-657	5.52	5.63	5.55
TC04G028780	PR-10	TC04G028780 _3UTR0:1-285	10.15	9.74	5.06
TC04G028780	PR-10	TC04G028780 :1-477	10.01	9.49	5.75
TC04G028860	PR-10	TC04G028860 _3UTR0:1-133	8.63	11.14	5.41
TC04G028860	PR-10	TC04G028860 :1-477	7.63	9.67	5.68
TC04G028790	PR-10	TC04G028790 :1-480	5.48	5.37	5.24
TC04G028880	PR-10	TC04G028880 :1-483	8.23	8.19	6.63
TC04G028900	PR-10	TC04G028900 _3UTR0:1-186	7.88	7.14	6.52
TC04G028900	PR-10	TC04G028900 :1-483	7.49	7.40	6.64
TC04G028760	PR-10	TC04G028760 :1-594	5.87	5.60	6.07
TC01G031100	PR-10	TC01G031100 _3UTR0:1-305	14.89	15.34	9.77
TC01G031100	PR-10	TC01G031100 :1-480	14.26	14.85	8.75
TC05G000380	PR-10	TC05G000380 :1-843	5.17	5.52	5.44
TC04G028740	PR-10	TC04G028740 _3UTR0:1-96	8.74	6.90	5.37
TC04G028740	PR-10	TC04G028740 :1-483	9.10	7.19	5.85

TC04G028750	PR-10	TC04G028750 _3UTR0:1-188	7.33	7.22	7.14
TC04G028750	PR-10	TC04G028750 :1-483	12.34	11.92	11.00
TC00G031750	PR-10	TC00G031750 _3UTR0:1-3533	5.09	5.15	5.23
TC00G031750	PR-10	TC00G031750 :1-360	7.94	9.15	5.33
TC10G014440	PR-10	TC10G014440 _3UTR1:1-96	9.91	10.54	6.88
TC10G014440	PR-10	TC10G014440 :1-366	10.28	11.45	6.04
TC04G028940	PR-10	TC04G028940 _3UTR0:1-71	6.57	6.95	5.66
TC04G028940	PR-10	TC04G028940 :1-351	7.88	9.86	6.25
TC09G003110	PR-11	TC09G003110 _3UTR0:1-158	9.48	8.51	6.35
TC09G003110	PR-11	TC09G003110 :1-1107	8.21	7.46	6.14
TC09G003140	PR-11	TC09G003140 :1-2145	9.28	9.18	7.67
TC09G003120	PR-11	TC09G003120 :1-2400	6.00	5.54	5.78
TC09G003150	PR-11	TC09G003150 :1-2313	8.57	8.46	8.40
TC09G003130	PR-11	TC09G003130 :1-1281	6.55	6.41	6.01
TC09G003180	PR-11	TC09G003180 :1-2250	5.45	5.88	5.79
TC09G003190	PR-11	TC09G003190 _3UTR0:1-122	9.87	9.22	9.64
TC09G003190	PR-11	TC09G003190 :1-1140	10.87	10.21	10.51
TC09G001640	PR-11	TC09G001640 :1-1389	9.06	8.37	6.57
TC09G003200	PR-11	TC09G003200 :1-1266	5.45	5.42	5.38
TC09G003160	PR-11	TC09G003160 :1-978	5.70	5.44	5.65
TC00G043900	PR-11	TC00G043900 _3UTR0:1-965	5.63	5.55	5.61
TC00G043900	PR-11	TC00G043900 :1-351	5.68	5.65	5.97
TC02G006630	PR-12	TC02G006630 :1-285	5.11	5.43	5.05

TC02G006660	PR-12	TC02G006660:1-171	5.48	5.26	5.61
TC08G002440	PR-12	TC08G002440:1-249	5.60	5.45	5.39
TC04G016400	PR-14	TC04G016400_3UTR0:1-139	15.31	15.22	15.42
TC04G016400	PR-14	TC04G016400_3UTR1:1-141	10.93	10.64	10.72
TC04G016400	PR-14	TC04G016400:1-420	15.05	14.95	14.96
TC04G016380	PR-14	TC04G016380_3UTR0:1-326	5.39	5.74	5.66
TC04G016380	PR-14	TC04G016380:1-390	6.61	6.62	7.34
TC04G016440	PR-14	TC04G016440_3UTR1:1-1271	5.65	5.68	5.92
TC04G016440	PR-14	TC04G016440:1-369	6.21	6.27	6.30
TC09G035160	PR-14	TC09G035160_3UTR0:1-269	13.67	11.45	6.80
TC09G035160	PR-14	TC09G035160:1-360	13.83	11.64	7.76
TC04G016450	PR-14	TC04G016450_3UTR0:1-4065	5.74	5.65	5.68
TC04G016450	PR-14	TC04G016450:1-459	10.85	11.20	10.95
TC09G035150	PR-14	TC09G035150_3UTR0:1-165	10.28	8.81	5.46
TC09G035150	PR-14	TC09G035150:1-351	11.39	9.51	6.35
TC06G001000	PR-14	TC06G001000_3UTR0:1-222	7.17	6.92	6.65
TC06G001000	PR-14	TC06G001000:1-348	5.67	5.14	5.21
TC01G034520	PR-14	TC01G034520_3UTR0:1-152	5.87	5.55	5.59
TC01G034520	PR-14	TC01G034520:1-393	5.46	5.33	5.50
TC01G039190	PR-14	TC01G039190:1-324	6.37	6.17	6.47
TC03G023690	PR-14	TC03G023690:1-360	12.05	12.33	12.31
TC04G013310	PR-14	TC04G013310_3UTR0:1-167	6.12	6.72	6.18
TC04G013310	PR-14	TC04G013310	5.30	5.20	5.38

		:1-369			
TC10G016320	PR-14	TC10G016320 _3UTR0:1-314	5.43	5.61	6.24
TC10G016320	PR-14	TC10G016320 :1-375	6.99	7.28	8.29
TC02G016580	PR-14	TC02G016580 :1-348	5.70	5.53	5.30
TC06G020010	PR-14	TC06G020010 _3UTR0:1-770	5.57	5.42	5.85
TC06G020010	PR-14	TC06G020010 :1-348	7.24	6.94	7.28
TC06G020030	PR-14	TC06G020030 :1-492	5.56	5.52	5.80
TC06G000990	PR-14	TC06G000990 _3UTR1:1-229	6.55	5.83	5.96
TC06G000990	PR-14	TC06G000990 :1-288	5.48	5.35	5.45
TC03G009350	PR-16	TC03G009350 _3UTR0:1-197	12.98	12.99	11.60
TC03G009350	PR-16	TC03G009350 :1-666	12.87	12.93	11.70
TC05G025530	PR-16	TC05G025530 :1-867	6.78	5.79	5.72
TC05G025440	PR-16	TC05G025440 _3UTR0:1-311	7.40	6.89	6.28
TC05G025440	PR-16	TC05G025440 :1-672	13.42	12.18	11.00
TC05G025420	PR-16	TC05G025420 _3UTR0:1-328	9.47	8.34	7.54
TC05G025420	PR-16	TC05G025420 :1-675	13.91	12.57	11.06
TC05G025400	PR-16	TC05G025400 _3UTR0:1-125	6.34	5.55	5.29
TC05G025400	PR-16	TC05G025400 :1-678	5.81	5.74	5.53
TC05G025410	PR-16	TC05G025410 _3UTR0:1-1027	5.63	5.94	5.49
TC05G025410	PR-16	TC05G025410 _3UTR1:1-537	5.12	5.81	6.36
TC05G025410	PR-16	TC05G025410 :1-678	9.06	7.95	7.38
TC05G025330	PR-16	TC05G025330 _3UTR0:1-127	5.23	5.31	5.29
TC05G025330	PR-16	TC05G025330 :1-666	4.97	5.31	5.35
TC05G031880	PR-16	TC05G031880	12.04	12.05	11.70

		_3UTR0:1-275			
TC05G031880	PR-16	TC05G031880 :1-654	12.91	12.87	12.49
TC05G024700	PR-16	TC05G024700 _3UTR1:1-131	5.35	5.41	5.21
TC05G024700	PR-16	TC05G024700 :1-663	6.01	6.02	5.92
TC05G025450	PR-16	TC05G025450 _3UTR0:1-167	7.43	6.91	6.35
TC05G025450	PR-16	TC05G025450 :1-681	9.50	8.08	6.88
TC05G025350	PR-16	TC05G025350 :1-681	5.75	6.27	5.92
TC05G025310	PR-16	TC05G025310 _3UTR0:1-175	7.53	7.72	7.37
TC05G025310	PR-16	TC05G025310 :1-678	7.39	7.76	7.40
TC05G025520	PR-16	TC05G025520 _3UTR0:1-2210	5.28	5.46	5.36
TC05G025520	PR-16	TC05G025520 :1-786	12.63	11.61	10.34
TC06G000370	PR-16	TC06G000370 :1-657	6.37	6.38	6.32
TC01G033170	PR-16	TC01G033170 :1-660	15.16	15.13	14.17
TC00G037180	PR-16	TC00G037180 :1-657	10.71	10.56	9.96
TC05G008860	PR-16	TC05G008860 :1-795	7.54	7.20	7.00
TC10G009710	PR-16	TC10G009710 _3UTR0:1-282	11.52	10.68	9.59
TC10G009710	PR-16	TC10G009710 :1-642	10.66	9.75	8.72
TC09G007080	PR-16	TC09G007080 _3UTR1:1-235	14.81	14.41	13.93
TC09G007080	PR-16	TC09G007080 :1-624	14.42	13.72	13.10
TC05G025480	PR-16	TC05G025480 :1-534	9.86	8.58	7.38
TC00G013300	PR-16	TC00G013300 :1-612	13.35	13.06	12.59
TC05G025360	PR-16	TC05G025360 :1-615	5.40	5.40	5.44
TC03G016120	PR-16	TC03G016120 _3UTR0:1-262	5.75	5.59	5.50
TC03G016120	PR-16	TC03G016120	5.72	6.09	6.09

		:1-2724			
TC05G025470	PR-16	TC05G025470 :1-480	6.22	6.07	5.87
TC06G009700	PR-16	TC06G009700 :1-879	5.38	5.67	5.70
TC05G025430	PR-16	TC05G025430 :1-573	5.69	6.38	6.28
TC00G054490	PR-16	TC00G054490 :1-2223	5.18	5.13	5.20
TC09G019920	PR-16	TC09G019920 :1-633	5.73	5.91	5.99
TC05G024940	PR-16	TC05G024940 :1-624	7.14	7.23	7.15
TC07G004510	PR-16	TC07G004510 :1-708	5.38	5.43	5.47
TC05G009390	PR-16	TC05G009390 _3UTR0:1-86	7.43	8.07	8.60
TC05G009390	PR-16	TC05G009390 :1-2814	6.07	6.31	6.71
TC09G019910	PR-16	TC09G019910 :1-618	5.93	5.58	5.73
TC00G076490	PR-16	TC00G076490 :1-516	5.57	5.84	5.67
TC06G000400	PR-16	TC06G000400 :1-342	5.56	5.37	5.37
TC00G054500	PR-16	TC00G054500 :1-450	5.22	5.22	5.26
TC07G004580	PR-16	TC07G004580 :1-411	5.32	5.29	5.34
TC07G004710	PR-16	TC07G004710 :1-468	5.25	5.20	5.22
TC02G009630	PR-17	TC02G009630 _3UTR0:1-153	6.02	5.57	5.62
TC02G009630	PR-17	TC02G009630 :1-681	6.57	6.09	5.53
TC02G009600	PR-17	TC02G009600 :1-765	12.08	11.81	5.91
TC02G009590	PR-17	TC02G009590 :1-678	7.94	9.06	5.82
TC02G009610	PR-17	TC02G009610 _3UTR0:1-440	9.80	9.19	5.46
TC02G009610	PR-17	TC02G009610 :1-705	10.26	9.62	5.58
TC02G009650	PR-17	TC02G009650 :1-624	6.61	5.70	5.47
