# Supplementary material for: Theobroma cacao L. pathogenesis-related gene tandem array members show diverse expression dynamics in response to pathogen colonization
Source: BMC Genomics. 2016 May 17;17:363. doi: 10.1186/s12864-016-2693-3 (PMC4869279; doi:10.1186/s12864-016-2693-3)
Supplement: Additional file 16: Figure S4. — Whole genome gene expression profiles in microarray-analyzed leaves. Scatterplots of log2 normalized expression values for all probes on the microarray, comparing pathogen treatments with water treatment. (PDF 4169 kb) [file 12864_2016_2693_MOESM16_ESM.pdf]

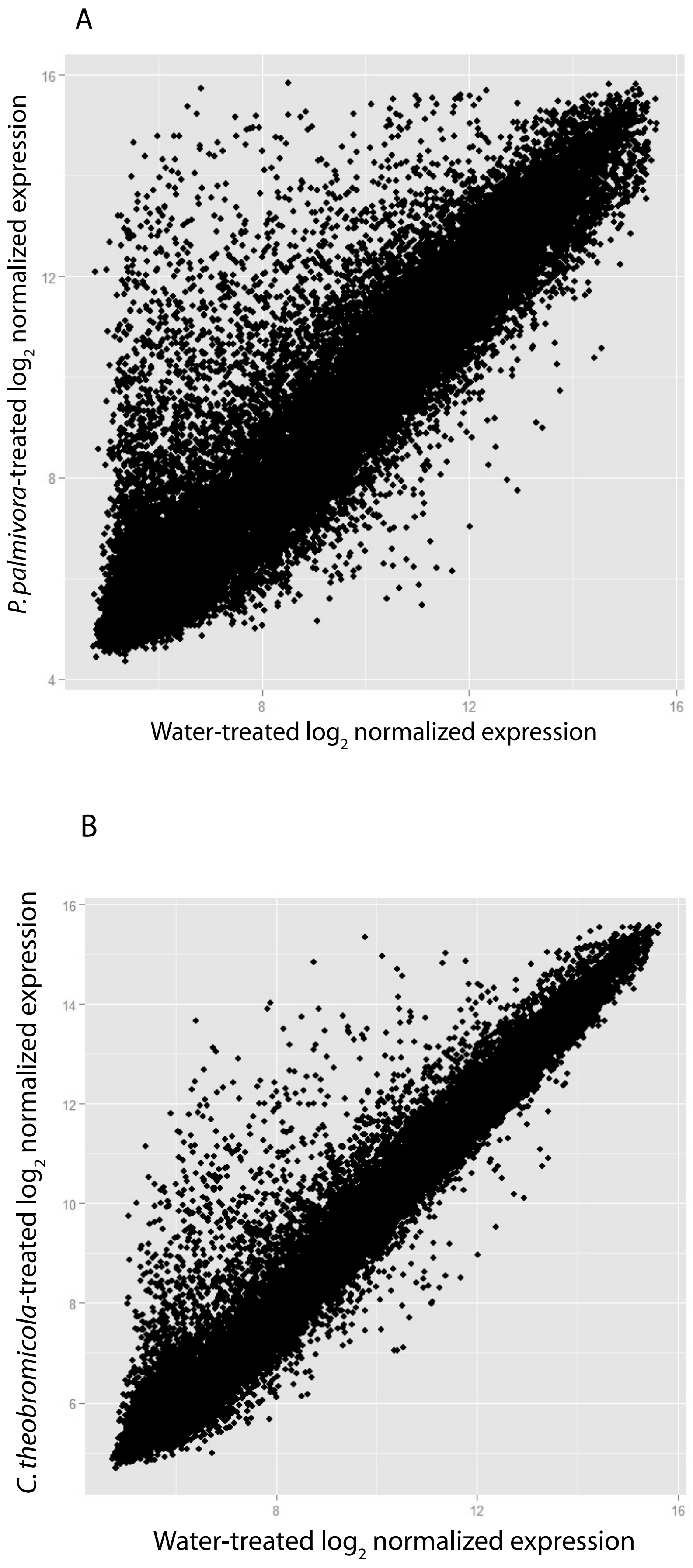

Figure S2. Whole genome expression profiles in pathogen infected leaves. Scatterplots of $\log_2$ normalized expression values for all probes on the microarray, comparing A) *Phytophthora palmivora* and water treatment and B) *Colletotrichum theobromicola* and water treatment.
