# Supplementary material for: Theobroma cacao L. pathogenesis-related gene tandem array members show diverse expression dynamics in response to pathogen colonization
Source: BMC Genomics. 2016 May 17;17:363. doi: 10.1186/s12864-016-2693-3 (PMC4869279; doi:10.1186/s12864-016-2693-3)
Supplement: Additional file 17: Table S14. — Log2 fold change for all significantly regulated (Benjamini-Hochberg p < 0.05) PR genes on microarray. (PDF 4169 kb) [file 12864_2016_2693_MOESM17_ESM.pdf]

**Supplemental Table S14 - Log$_2$ fold induction for all significantly regulated (Benjamini-Hochberg $p < 0.05$) PR genes on microarray.**			
**TcID**	**Class**	**Log$_2$ Fold Induction by *P. palmivora***	**Log$_2$ Fold Induction by *C. theobromicola***
TC02G002410	PR-1	6.969214921	5.803005844
TC00G083950	PR-2	5.481485868	5.274902853
TC02G028070	PR-2	-1.007284112	0
TC04G012520	PR-2	-1.004855831	0
TC04G029300	PR-2	7.038733873	5.124632397
TC05G016070	PR-2	3.471711585	3.852450373
TC09G024130	PR-2	3.091005297	3.525725486
TC09G031660	PR-2	0.931173332	0
TC01G000770	PR-3	5.062394087	4.232396812
TC01G000800	PR-3	4.204702138	0
TC01G032950	PR-3	1.664342235	0
TC02G003890	PR-3	4.762007095	4.575789895
TC04G018090	PR-3	2.245266721	0
TC04G018100	PR-3	4.486635225	3.058348476
TC04G018110	PR-3	4.868411348	3.524123658
TC04G018160	PR-3	5.987219197	6.191667463
TC05G027210	PR-4	4.635764914	3.904701358
TC05G027220	PR-4	3.465605498	2.74290496
TC05G027320	PR-4	5.73783208	4.153153375
TC00G056070	PR-5	4.451767113	0
TC00G060970	PR-5	3.180438493	4.230680177
TC03G026990	PR-5	3.914339712	4.075639354
TC03G027000	PR-5	2.187517329	2.553509804
TC03G027010	PR-5	6.323194993	4.724213803
TC03G027030	PR-5	3.052693754	3.127005464
TC05G022770	PR-6	1.183812493	0
TC10G005840	PR-6	3.835164484	0
TC10G005870	PR-6	4.563892059	3.899141195
TC10G005890	PR-6	3.680034051	0
TC10G005920	PR-6	5.657179105	3.836989675
TC00G032610	PR-7	-0.648659534	0
TC01G037020	PR-7	2.035243353	0
TC03G022580	PR-7	3.765800623	2.700390846

TC06G000810	PR-7	-0.690191189	0
TC08G000230	PR-7	-1.338701774	0
TC01G035050	PR-8	3.469055016	3.86143576
TC03G017760	PR-8	5.029322581	5.186825269
TC00G014230	PR-9	1.368199733	0
TC00G045400	PR-9	5.477616751	3.822067416
TC00G045460	PR-9	1.427169267	0
TC00G045610	PR-9	3.837201882	3.813211387
TC00G045630	PR-9	2.217918663	0
TC01G004590	PR-9	1.830104938	2.002740813
TC02G011950	PR-9	0	1.782274543
TC02G012020	PR-9	1.266651919	1.90791588
TC04G004370	PR-9	0	1.795617951
TC05G006120	PR-9	2.715351743	0
TC05G031640	PR-9	3.883928429	0
TC06G014950	PR-9	-1.484519935	0
TC08G013300	PR-9	1.988816031	0
TC09G034930	PR-9	3.087674485	0
TC10G015970	PR-9	1.88389408	1.792325945
TC00G031750	PR-10	2.611666618	3.82153607
TC01G031100	PR-10	5.316404504	5.84017564
TC04G028740	PR-10	3.307337415	0
TC04G028750	PR-10	1.342116344	0
TC04G028780	PR-10	4.673157106	4.677570649
TC04G028860	PR-10	2.588858057	5.7382445
TC04G028900	PR-10	1.354054231	0
TC04G028940	PR-10	0	3.610891829
TC10G014440	PR-10	3.63370518	4.528033057
TC09G001640	PR-11	2.498752807	1.799829394
TC09G003110	PR-11	2.604179538	2.161760349
TC09G003140	PR-11	1.611253048	1.512622369
TC09G035150	PR-14	4.925714025	3.253387745
TC09G035160	PR-14	6.471086738	4.26631556
TC03G009350	PR-16	1.274992795	1.221255725
TC05G009390	PR-16	-1.17371423	0
TC05G025410	PR-16	1.687828981	0
TC05G025420	PR-16	2.38964617	0
TC05G025440	PR-16	1.771075747	0
TC05G025450	PR-16	2.620742802	0
TC05G025480	PR-16	2.47117974	0
TC05G025520	PR-16	2.293160405	0

TC02G009590	PR-17	2.120548009	3.244115829
TC02G009600	PR-17	6.168643409	5.90180022
TC02G009610	PR-17	4.503543332	3.884228221
