# Supplementary material for: Theobroma cacao L. pathogenesis-related gene tandem array members show diverse expression dynamics in response to pathogen colonization
Source: BMC Genomics. 2016 May 17;17:363. doi: 10.1186/s12864-016-2693-3 (PMC4869279; doi:10.1186/s12864-016-2693-3)
Supplement: Additional file 18: Table S15. — Sequences of qRT-PCR primers for validation of PR-1, PR-3, and PR-4 family expression. (PDF 4169 kb) [file 12864_2016_2693_MOESM18_ESM.pdf]

Table S15. Sequences of qRT-PCR primers. Gene IDs are included in primer name.		
	**Sequence Name**	**Sequence**
**PR-1s**	Tc01_g003940F	GGCAGTGCTGCTGCCTAGTGC
	Tc01_g003940R	TGGTATCTCGCAAGCCGGCTCG
	Tc01_g034430F	TGCGGAGGATGCCGTCAGGTTA
	Tc01_g034430R	GACTCTGGCACATCCTAGTTTGGT
	Tc02_g002380F	CATCCTTTGCGTTGCCTTCCTTG
	Tc02_g002380R	TGCGCGTAGGCGGCCACTTG
	Tc02_g002390F	CTACGCCAACCAACGTAAGGGA
	Tc02_g002390R	AGGCCTTTCCGCCACCCACAAG
	Tc02_g002400F	CTGCAACCTCGAGCACTCCACT
	Tc02_g002400R	GTCTGTTGCACAAGTGTTAGTGTC
	Tc02_g002410F	GCAACCTCGTGCACTCCGGT
	Tc02_g002410R	CCTGGTGCACAAGTGTTAGAGGA
	Tc02_g002420F	CCTATTAGCTTCAACTCTAGTTCACGTT
	Tc02_g002420R	CGTAGGCCGCAACCTCGGG
	Tc02_g002430F	GACTTGGTGAACGCACACAATGCGAT
	Tc02_g002430R	GAATGCTCCAGGTTGCAGTCCCCAA
	Tc02_g010380F	CGATGAGAAAACCGATTATGACTTGA
	Tc02_g010380R	CAAACAACAAAAGTACCCCCATGACC
	Tc05_g005530F	TGCGAAGAAGATAAAGTCGGCTTCCTT
	Tc05_g005530R	TTAGCGCACTGTTTGGCGAAGTAG
	Tc09_g000720F	CAATATCTAACACCCCACAACATTCTAA
	Tc09_g000720R	CCCCAGAAGAGATTCTCCCCATAATTG
	Tc09_g016580F	AGCTTCAGCACTCTTTTCCAGAG
	Tc09_g016580R	ATTTTTCTTCCTCAGACCATGCAC
	Tc09_g016590F	TCATCGTTTTCCTACTGCTCATCTCAAC
	Tc09_g016590R	CTTTGGTTAGCATACCACTGAGCATATCG
	Tc10_g000980F	CTAGTGCGTGCAGTAGGCCCTACG
	Tc10_g000980R	CCATTATTTCTGCATCTAAATCCCTCGC
**PR-3s**	Tc01_g000770F	GTTGCCAGTATCGTGTCACCTGC
	Tc01_g000770R	GGCATGTGCAAAAAAAGCAGCAATCTC
	Tc02_g003890F	TTTCTCACGCACAGACAGTAGCGA
	Tc02_g003890F	TTGTAGCGAAGGCAGGGAAAGACT
	Tc04_g018100F	GGGACTACTGTGATGAGACCGTT
	Tc04_g018100R	GAGATAACAGGGTCTGTGGCGATA
	Tc04_g018110F	GGAGGTCCTTGTGTTAACAATGGTG
	Tc04_g018110R	CTCCTCAACTGTGCCAGTCCTTGCG
	Tc04_g018160F	GCACAACCGGTGACCTTACTACC

	Tc04_g018160R	CTGTTCCCTTATAAAGCAATATCCCCATG
	Tc06_g000440F	GATGACTCCTACAAGTATGTG
	Tc06_g000440R	GGAAGGCAAGAGTAGCATTCTGCT
**PR-4s**	Tc00_g012980F	AATGCTGTGAGTGCTTATTGCTCAACTTG
	Tc00_g012980R	CACAATTGCTTCAGCTCTTGTACCTC
	Tc05_g027210F	CTTTTGTGGTCCAGCTGGTCCTCGA
	Tc05_g027210R	GCAATGCCGTTCCCATTTGTGTCAAGTT
	Tc05_g027220F	TTTAAACCCATTGACACTGATGGAAAC
	Tc05_g027220R	CCCAGCTATTCTGTGAAGGTTG
	Tc05_g027230F	TAATCCCATTGACACTGATGGACGA
	Tc05_g027230R	CCATTCCAAGGGCTTATCACCAACCT
	Tc05_g027250F	GAATCGTTGACACATGCGGCATTGAC
	Tc05_g027250R	TCGTCGTCACAATCCACAACCTTG
	Tc05_g027320F	GTTTCGAGGTTGCTTGTCTTTTTGGTT
	Tc05_g027320R	CAGGCAAACAATAGGCATCGGTG
	Tc10_g011130F	TAAACCCATTGACACTGATGGAAAA
	Tc10_g011130R	CCATTCCAAGGGCTTATCACCATCCA
**PR-10s**	Tc01_g031100F	TTGAGGGTGATGTAATTGGTGACAAG
	Tc01_g031100R	CCTCCACGACTTTGTACATTCCGAC
	Tc04_g028780F	CATCCCCAAGATTGTTCCCAAGGCCA
	Tc04_g028780R	CAGTATGTGAAGTTGTCTTTGTCAAC
	Tc04_g028860F	GACCAAGAAAATTTCACCTGCTGCTTC
	Tc04_g028860R	GCCTTTATTTCCTCCTCCTTGATATTG
**Tubulin**	Tc06_g000360F	GGAGGAGTCTCTATAAGCTTGCAGTTGG
	Tc06_g000360R	ACATAAGCATAGCCAGCTAGAGCCAG
