# Supplementary material for: Theobroma cacao L. pathogenesis-related gene tandem array members show diverse expression dynamics in response to pathogen colonization
Source: BMC Genomics. 2016 May 17;17:363. doi: 10.1186/s12864-016-2693-3 (PMC4869279; doi:10.1186/s12864-016-2693-3)
Supplement: Additional file 19: Table S1. — PR gene gamily type members: GenBank accession numbers for PR type member amino acid sequences used as BLASTp queries (PDF 4169 kb) [file 12864_2016_2693_MOESM19_ESM.pdf]

Supplemental Table S1 - GenBank Accession Numbers for PR Family Type Members

PR Gene	Accession Number
PR-1	CAA31008.1
PR-2	AAA34103.1
PR-3	P17514.1
PR-4	CAA41437.1
PR-5	BAA74546.2
PR-6	AAA34183.1
PR-7	NP_001234257.1
PR-8	AAA33120.1
PR-9	AAA34108.1
PR-10	AAL09033.1
PR-11	CAA54373.1
PR-12	O24332.1
PR-13	AAC41678.1
PR-14	CAA91436.1
PR-15	CAA74595.1
PR-16	CAA63659.1
PR-17	BAA81904.1
