# Supplementary material for: Theobroma cacao L. pathogenesis-related gene tandem array members show diverse expression dynamics in response to pathogen colonization
Source: BMC Genomics. 2016 May 17;17:363. doi: 10.1186/s12864-016-2693-3 (PMC4869279; doi:10.1186/s12864-016-2693-3)
Supplement: Additional file 20: Figure S5. — Representative photographs showing leaves 72 h after A) H2O, B) C. theobromicola (with red lines indicating developing lesions), and C) P. palmivora treatment. Scale bars represent 1 cm. (PDF 4169 kb) [file 12864_2016_2693_MOESM20_ESM.pdf]

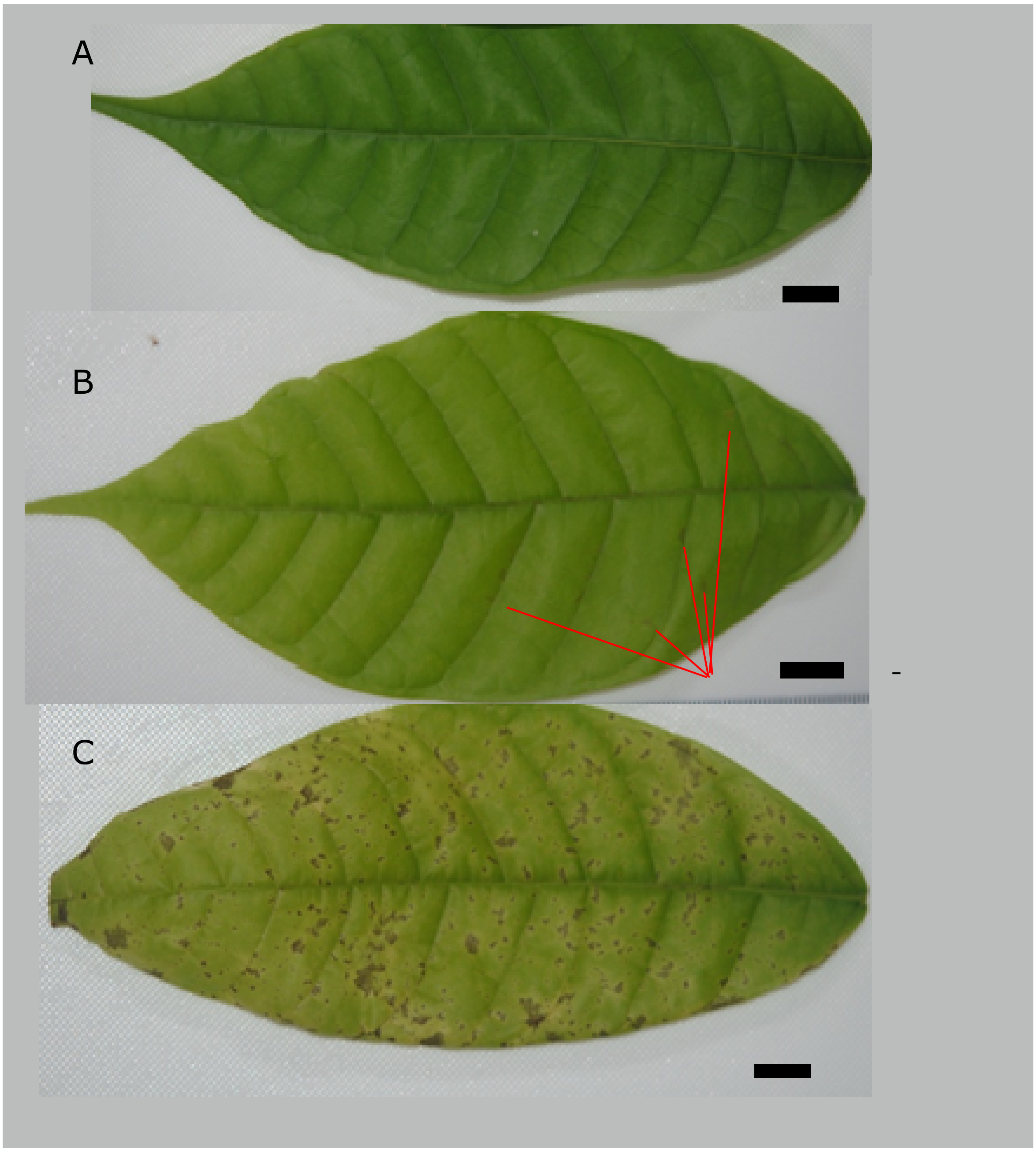

Figure S5. Representative images of leaves 72 h after treatment with A) distilled water, B) *Colletotrichum theobromicola*, and C) *Phytophthora palmivora*. Scale bars represent 1 cm. Red lines in B) indicate spots of tissue where lesions have begun to develop.
